# ClinPrior: an algorithm for diagnosis and novel gene discovery by network-based prioritization

**DOI:** 10.1186/s13073-023-01214-2

**Published:** 2023-09-07

**Authors:** Agatha Schlüter, Valentina Vélez-Santamaría, Edgard Verdura, Agustí Rodríguez-Palmero, Montserrat Ruiz, Stéphane Fourcade, Laura Planas-Serra, Nathalie Launay, Cristina Guilera, Juan José Martínez, Christian Homedes-Pedret, M. Antonia Albertí-Aguiló, Miren Zulaika, Itxaso Martí, Mónica Troncoso, Miguel Tomás-Vila, Gemma Bullich, M. Asunción García-Pérez, María-Jesús Sobrido-Gómez, Eduardo López-Laso, Carme Fons, Mireia Del Toro, Alfons Macaya, HSP/ataxia workgroup, Àngels García-Cazorla, Antonio José Ortiz-Martínez, Carlos Ignacio-Ortez, Cristina Cáceres-Marzal, Eduardo Martínez-Salcedo, Elisabet Mondragón, Estíbaliz Barredo, Ileana Antón Airaldi, Javier Ruiz Martínez, Joaquin A. Fernández Ramos, Juan Francisco Vázquez, Laura Díez-Porras, María Vázquez-Cancela, Mar O’Callaghan, Tamara Pablo Sánchez, Velina Nedkova, Ana Isabel Maraña Pérez, Sergi Beltran, Luis G. Gutiérrez-Solana, Luis A. Pérez-Jurado, Sergio Aguilera-Albesa, Adolfo López de Munain, Carlos Casasnovas, Aurora Pujol

**Affiliations:** 1 Neurometabolic Diseases Laboratory, Bellvitge Biomedical Research Institute (IDIBELL), Hospital Duran i Reynals, Gran Via 199, L’Hospitalet de Llobregat, Barcelona, 08908 Spain; 2 Centro de Investigación Biomédica en Red de Enfermedades Raras (CIBERER), ISCIII, Madrid, Spain; 3 Neurology Department, Neuromuscular Unit, Bellvitge University Hospital, Universitat de Barcelona, Barcelona, Spain; 4 Pediatric Neurology Unit, Pediatrics Department, Hospital Universitari Germans Trias i Pujol, Universitat Autònoma de Barcelona, Barcelona, Spain; 5 Neurology Department, Hospital Universitari General de Catalunya, Barcelona, Spain; 6 Neuromuscular Area, Group of Neurodegenerative Diseases, Biodonostia Health Research Institute (Biodonostia HRI), San Sebastian, Spain; 7 Network Center for Biomedical Research in Neurodegenerative Diseases (CIBERNED), ISCIII, Madrid, Spain; 8 Pediatric Neurology Department, Donostia University Hospital, University of the Basque Country (UPV-EHU), San Sebastian, Spain; 9 Pediatric Neurology Department, Central Campus, Hospital Clínico San Borja Arriarán, Universidad de Chile, Santiago, Chile; 10 Neuropediatrics Department, Hospital Universitari i Politècnic La Fe, Valencia, Spain; 11 Centro Nacional Análisis Genómico (CNAG) - Centre for Genomic Regulation (CRG), The Barcelona Institute of Science and Technology, Baldiri Reixac 4, Barcelona, Spain; 12 Pediatric Neurology Unit, Pediatrics Department, Hospital Universitario Fundación Alcorcón, Madrid, Spain; 13 Coruña Institute of Biomedical Research (INIBIC), A Coruña, Spain; 14 Hospital Clínico Universitario, A Coruña, Spain; 15 Pediatric Neurology Unit, Pediatrics Department, Reina Sofía University Hospital, Córdoba, Spain; 16 Maimonides Institute For Biomedical Research of Cordoba (IMIBIC), Córdoba, Spain; 17 Pediatric Neurology Department, Sant Joan de Déu University Hospital, Member of the ERN EpiCARE, Barcelona, Spain; 18 Sant Joan de Déu Research Institute, (IRSJD), Barcelona, Spain; 19 Pediatric Neurology Department, Vall d’Hebron University Hospital, Universitat Autònoma de Barcelona, Barcelona, Spain; 20 Pediatric Neurology Research Group, Vall d’Hebron Research Institute (VHIR), Universitat Autònoma de Barcelona, Barcelona, Spain; 21 Universitat Pompeu Fabra (UPF), Barcelona, Spain; 22 Departament de Genètica, Facultat de Biologia, Microbiologia i Estadística, Universitat de Barcelona (UB), Barcelona, 08028 Spain; 23 Pediatric Neurology Department, Children’s University Hospital Niño Jesús, Madrid, Spain; 24 Genetics Service, Hospital del Mar Research Institute (IMIM), Barcelona, Spain; 25 Department of Experimental and Health Sciences, Universitat Pompeu Fabra, Barcelona, Spain; 26 Pediatric Neurology Unit, Pediatrics Department, Navarra Health Service, Pamplona, Spain; 27 Navarrabiomed, Biomedical Research Center, Pamplona, Spain; 28 Neurology Department, Donostia University Hospital, San Sebastian, Spain; 29 Catalan Institution of Research and Advanced Studies (ICREA), Barcelona, Catalonia Spain

**Keywords:** Algorithm, WES/WGS, HPOs, Variant prioritization, Interactome, Hereditary spastic paraplegia, Cerebellar ataxia, Candidate gene

## Abstract

**Background:**

Whole-exome sequencing (WES) and whole-genome sequencing (WGS) have become indispensable tools to solve rare Mendelian genetic conditions. Nevertheless, there is still an urgent need for sensitive, fast algorithms to maximise WES/WGS diagnostic yield in rare disease patients. Most tools devoted to this aim take advantage of patient phenotype information for prioritization of genomic data, although are often limited by incomplete gene-phenotype knowledge stored in biomedical databases and a lack of proper benchmarking on real-world patient cohorts.

**Methods:**

We developed ClinPrior, a novel method for the analysis of WES/WGS data that ranks candidate causal variants based on the patient’s standardized phenotypic features (in Human Phenotype Ontology (HPO) terms). The algorithm propagates the data through an interactome network-based prioritization approach. This algorithm was thoroughly benchmarked using a synthetic patient cohort and was subsequently tested on a heterogeneous prospective, real-world series of 135 families affected by hereditary spastic paraplegia (HSP) and/or cerebellar ataxia (CA).

**Results:**

ClinPrior successfully identified causative variants achieving a final positive diagnostic yield of 70% in our real-world cohort. This includes 10 novel candidate genes not previously associated with disease, 7 of which were functionally validated within this project. We used the knowledge generated by ClinPrior to create a specific interactome for HSP/CA disorders thus enabling future diagnoses as well as the discovery of novel disease genes.

**Conclusions:**

ClinPrior is an algorithm that uses standardized phenotype information and interactome data to improve clinical genomic diagnosis. It helps in identifying atypical cases and efficiently predicts novel disease-causing genes. This leads to increasing diagnostic yield, shortening of the diagnostic Odysseys and advancing our understanding of human illnesses.

**Supplementary Information:**

The online version contains supplementary material available at 10.1186/s13073-023-01214-2.

## Background

In the past few years, the clinical application of next-generation sequencing (NGS) techniques (whole-exome sequencing (WES) and whole-genome sequencing (WGS)) has significantly increased both the diagnostic yield and our knowledge of hereditary diseases. In particular, WES has enabled a striking increase in the discovery of novel disease-causing genes and a broadening of disease phenotypes [1]. However, the overall diagnostic yield of WES in neurological diseases ranges from approximately 25% in heterogeneous cohorts to over 50% in enriched, curated cohorts (i.e., cohorts defined by well-defined phenotypes, positive family histories, or consanguinity) [2] (Additional file 1: Table S1). Despite continuous advances, the analysis of NGS data poses the challenges of variant selection and interpretation, which are especially relevant for cases in which the causal gene has not yet been associated with a disease.

Several computational methods have recently been developed to use patient phenotype information for disease-gene prioritization of genomic data [3, 4]. These methods generally compute the similarity between a patient’s phenotype and candidate diseases by leveraging gene-phenotype associations from databases such as OMIM [5] or DisGeNet [6]. Several of these methods are combined with network-based approaches that integrate different levels of biological organization, ranging from the genome to the transcriptome and the phenome, to enhance finding the most phenotypically similar matches [7–9]. Nevertheless, these methods are limited by their reliance on incomplete knowledge of diseases and associated genes and poor validation on large-scale clinical sequencing cohorts.

We present here ClinPrior, a new method that ranks candidate causal variants/genes of patients sequenced by NGS methods according to their phenotype (using standardized Human Phenotype Ontology -HPO- terms) by performing interactome network-based prioritization. First, we demonstrate the effectiveness of our method by evaluating its performance on comprehensive computational simulations in synthetic cohort scenarios. Second, we present ClinPrior’s diagnostic performance in a widely heterogeneous prospective, real-world series of 135 families affected by hereditary spastic paraplegia (HSP) and/or cerebellar ataxia (CA). ClinPrior was successfully used to prioritize pathogenic causative variants (mostly single-nucleotide variants (SNVs) or small insertion/deletion variants (INDELs)), strongly contributing to a final 70% positive diagnostic yield. Finally, we generated an HSP/CA-specific interactome using previously generated interactome knowledge, which will enable the discovery of novel disease genes in the future.

## Methods

### ClinPrior: Interactome-driven prioritization method

**Fig. 1:**
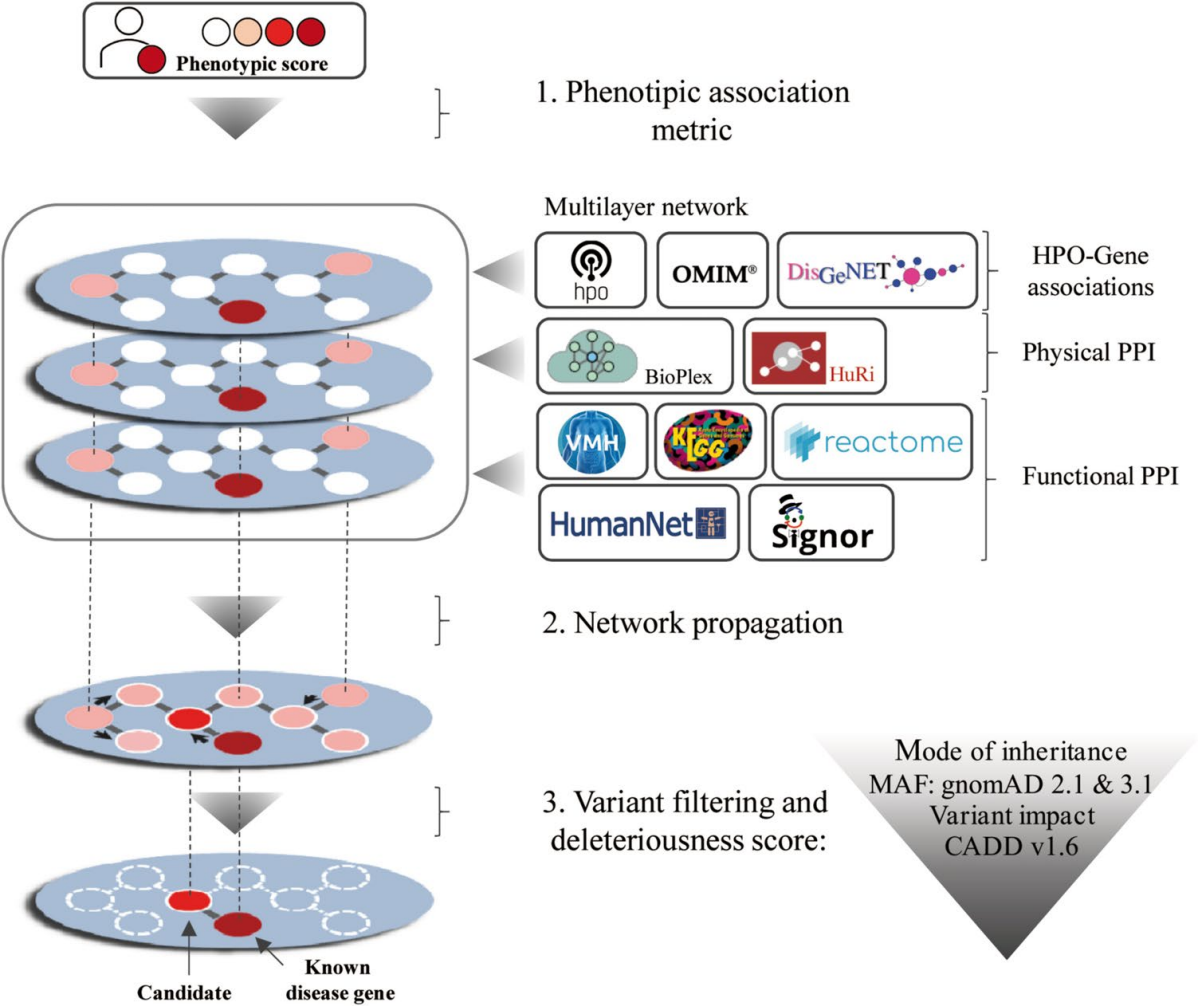
ClinPrior pipeline. First, the algorithm calculates the phenotypic association metric for each gene in the phenotypic layer based on the patient’s phenotype and known HPO-gene associations. The multilayer network is built from different data resources (see “ Methods”). The phenotypic layer reports HPO-gene associations, the physical layer reports physical protein‒protein interactions (PPIs) and the functional layer provides coexpression, signalling or metabolic pathway, and protein domain associations. The method propagates the phenotypic metric in adjacent nodes of the network so that higher scores indicate a better phenotypic fit with the patient. Variants resulting from patient genomic sequencing are filtered by frequency, variant impact and mode of inheritance. With this method, new candidate genes not previously associated with disease can also be identified thanks to the propagation of the phenotypic metric through neighbourhood connections

We developed a network-based prioritization algorithm structured by three main steps: (1) the calculation of a phenotype-matching metric by comparing patient phenotypes to data in existing human disease databases (referred as prior knowledge throughout this work); (2) iterative propagation of this phenotypic score within a gene‒gene network; and (3) variant filtering of VCF files and calculation of variant deleteriousness scores (Fig. 1).

#### Phenotypic score

ClinPrior compares patient clinical features with the phenotypic data associated with each node (gene) by calculating a phenotype association metric that measures the strength of association of each gene with the patient’s phenotype. To this aim, the patient’s clinical features are first translated into phenotypic ontology HPO terms (http://human-phenotype-ontology.github.io/), and compared with the 439,379 HPO-disease-gene associations of the phenotypic layer (OMIM, HPO site and DisGeNet databases [5, 6, 10]) based on a hypergeometric test that compares the number of patient HPO terms that associate with a specific gene. In addition, we considered the hierarchical structure of the HPO, as we also included the immediate HPO ancestor and descendant terms to perform the calculation (e.g. Abnormality of the hand > Abnormality of the finger (HPO present in patient) > Abnormality of the 5th finger). Then, we used the PRINCE logistic function to transform the phenotypic scores into a normalized value [0, 1] [7]).

#### Generation of a multilayer interactome network

ClinPrior propagates iteratively the phenotypic metric to adjacent nodes within a gene–gene interaction network with 23,509 genes and 699,854 different connections (physical and functional layers) after discarding nodes/genes with more than 1000 interactions. For the physical interactome layer, we integrated protein‒protein interactions (PPIs) from the BioPlex 2.0 Network [11] and the Human Reference Interactome (HuRI) [12], including the curated interactions from the scientific literature Lit-BM-13 dataset [13] and the high throughput Yeast-Two-Hybrid human proteome binary interactions HI-I-05 and HI-II-14 datasets [12–14]. For the functional interactome layer, we integrated functional interactions from the HumanNet-CF v.2 database (CX: coexpression, DB: common pathways in databases, DP: protein domain, GI: gene interaction, GN: gene neighbourhood, PG: phylogenetic profile and PI: protein‒protein interactions) [15], KEGG (genes connected by substrate-product reactions in metabolic pathways) [16], Recon3D metabolic database (discarding metabolites associated with more than 50 genes) [17] and Signor 2.0 database (signalling pathways) [18].

All gene‒gene interactions were initially assigned a value of 1, and afterwards, we built an adjacency matrix W, adjusted with node degree normalization that shows how specific is the association between each pair of genes in the interactome, both from the physical and functional layers.

#### Network propagation

Using as an input the phenotypic scores for all genes in the network (vector Y) and the gene‒gene interaction adjacency matrix (W), we calculated a network propagation score for the gene vector F using the Zhou et al. [19] equation: F_t+1_ = αF_t_ **W** + (1 − *α*)**Y**. We initialized F_0_ = Y and ran the analysis equation iteratively to convergence with *α* set to 0.2 (different values were tested, and we observed that the results were robust to the choice of *α*). This iterative propagation method is used in PRINCE [7] and applied in Novarino et al. [9].

#### Variant filtering and deleteriousness score

Patient variants extracted from a Variant Call Format (VCF) files from a WES/WGS experiment are analysed and filtered using Ensembl Variant Effect Predictor (VEP) and its filter tool [20]. For the real-world cohort, we applied the following criteria: (1) variants in the coding region; (2) an allele frequency lower than 1% in the autosomal recessive mode and lower than 0.005% in the autosomal dominant mode of inheritance in 1000 Genomes Project, NHLBI GO Exome Sequencing Project (ESP) and gnomAD (v2.1.1 for exomes and v3.1.2 for genomes) databases [21–23]; (3) genotype quality (GQ) in the VCF file higher than or equal to 20; (4) read depth (DP) in the VCF file higher than or equal to 10; (5) predicted deleterious effect on protein function, including frameshift insertions/deletions, nonsense and nonsynonymous amino acid substitutions and canonical splicing sites classified with high and moderate impact effects; and (5) noncanonical splicing sites, synonymous variants in splice regions and intronic variants less than 30 bp away from the splice site (in WES).

To score variant deleteriousness, ClinPrior considers (1) hypothesized mode of inheritance in homozygous, compound heterozygous, heterozygous or hemizygous variants; (2) variant impact classification on high, moderate and low prediction according to VEP annotation; (3) precalculated in silico CADD v1.6 predictor scores [24]; (3) splicing effect prediction for canonical and noncanonical variants using the MaxEntScan plugin in VEP [25]; (4) the gene-wide metrics pLI, pREC, *Z* scores for missense and loss of function from the gnomAD database [23]; and (5) variant constrained coding region scores for accurate identification of local intolerant missense variants with the assumption that the absence of variants in a given genomic position and its neighbourhoods is informative of change intolerance [26]. Finally, ClinPrior integrates the propagated phenotypic metric and the variant deleteriousness scores into a final ClinPrior score that determines the final order of the given variant in the ranked variant list for each case. We used the vcfR package to read VCF files in R [27].

The prioritization algorithm is implemented in the R library ClinPrior and is available at GitHub: https://github.com/aschluter/ClinPrior [28] and Zenodo repository: https://zenodo.org/record/7845939 [29].

### Patient enrolment and clinical recruitment

Study participants were identified at 19 child and adult neurology units from tertiary hospitals around Spain from April 2017 to December 2020. Informed consent was obtained from all patients. Patients were first classified as having (i) cerebellar ataxia, (ii) pure spastic paraplegia, or (iii) complex spastic paraplegia, including additional features such as cerebellar signs, sensorimotor neuropathy, white matter involvement or neurodevelopmental delay, among others. Extensive clinical evaluation to rule out acquired causes was performed at each centre of origin. A molecular diagnosis could not be established by the referring physicians despite the application of standard-of-care paraclinical studies (including mainly cranial and spinal magnetic resonance imaging (MRI), neurophysiological and genetic studies such as array comparative genomic hybridization (aCGH), targeted Sanger sequencing, MLPA or NGS gene panels), as well as metabolic workup when considered necessary. Polyglutamine expansions were excluded in all patients with suspected autosomal-dominant (*ATXN1*, *ATXN2*, *ATXN3*, *ATXN7*, *CACNA1A*, *TBP* and *ATN1*) or autosomal-recessive (*FXN* and *FMR1*) ataxias (*n* = 23), and negative results for the most frequently mutated genes causing dominant and recessive spastic paraplegia (*ATL1*, *SPAST*, *SPG7* and *SPG11*, among others) were obtained prior to WES in most patients with a predominant spastic phenotype (*n* = 43).

Clinical records were reviewed by two experienced neurologists and one paediatric neurologist at the Neurometabolic Disease Laboratory of Bellvitge Biomedical Research Institute (IDIBELL) and were translated into HPO terms. We annotated a mean of 24.4 HPOs per patient, with a median of 23. The minimum number of HPOs used in a patient was 5 and the maximum was 47. A novel phenotype was considered when a patient displayed striking clinical, radiological or biochemical features not previously described in the literature.

Further methods related to (1) benchmarking, (2) HSP/CA expanded network, (3) WES/WGS sequencing and (4) variant functional validation can be found in Additional file 1: Methods.

## Results

We have developed a variant prioritization module named ClinPrior, which identifies the most likely disease-causing variants in a VCF file associated with patient phenotypes using phenotype matching and, most importantly, a three-layer interactome. In this interactome, the nodes in each layer represent genes, whereas the links represent their respective relationship at a particular scale of biological organization, namely, direct physical, functional and phenotypic interactions between gene products, extracted from different open sources, as depicted in Fig. 1.

### Benchmarking of ClinPrior using a synthetic WES cohort

**Fig. 2:**
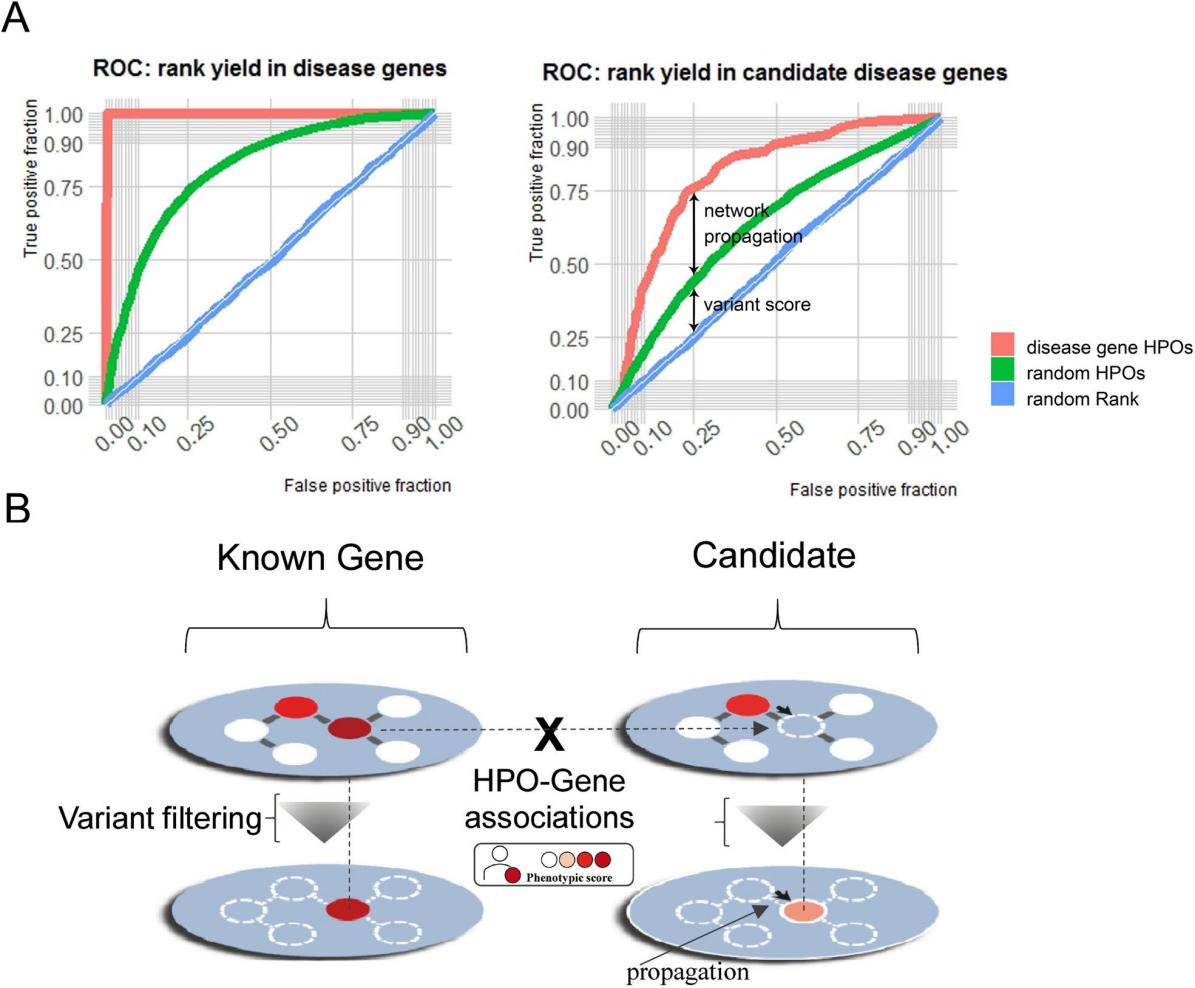
ClinPrior performance benchmarking in a synthetic cohort. **A** Variant prioritization performance through the area under the receiver operating characteristic curve (AUROC) in the identification of known disease genes and candidate disease genes (**A**). ROC curves computed using the patient HPO terms, random HPO terms and random final ClinPrior prioritization rank in the 66,800 synthetic WES analysed. **B** The method identifies the gene that best matches the patient’s phenotype based on known HPO-gene associations and the propagation of the phenotypic metrics in the multilayer interactome. When the identified gene is a novel candidate gene not previously linked to disease, there are no HPO-gene associations in the phenotypic layer. For benchmarking, we simulated a candidate gene by removing the HPO-Gene associations from each candidate

We first evaluated the performance of the algorithm by analysing the prioritization of 66,800 SNVs or small INDELs pathogenic variants present in 3356 different disease-associated genes obtained from the ClinVar database (December 2019) [30] (Fig. 2, VCF file in Zenodo [31]). For this purpose, we generated 66,800 synthetic exomes by inserting ClinVar pathogenic variants (one per exome) into a high-confidence gold standard exome VCF file published by the Genome in a Bottle (GIAB) consortium [32]. We used the HPOs linked to each gene containing variants, which are present in the HPO-gene associations in the phenotypic layer (OMIM, HPO and DisGeNet), to simulate the patient phenotypic features.

We assessed ClinPrior prediction performance through area under the receiver operating characteristic (AUROC) curve graphs in three different scenarios (Fig. 2A): (1) using all phenotypic information in HPOs associated with the genes of interest, (2) using the exact same number of HPOs associated with each gene, but now randomly chosen to simulate a situation where variant deleteriousness only drives prioritization, and (3) using no HPO information and assigning a random prioritization rank to variants in the VCF file. This third scenario is used to simulate a situation in which no variant deleteriousness or phenotypic information is available for prioritization. Its AUROC curve is represented as a straight line and is equivalent to obtaining the same value for the true and false-positive result fractions, AUROC value = 0.5. In the first scenario (all HPOs), the obtained AUROC curve value was 0.9994, and in the second scenario (random HPOs), the AUROC curve value was 0.8393, indicating the importance of phenotypic terms accurately matching patient clinical data to increase prioritization accuracy.

Given that genes associated with the same group of diseases are more connected in interactomes [7–9], we decided to examine whether our network had predictive power to discover new candidate genes not yet associated with disease. To assess this possibility, we removed the HPO-gene associations of candidate genes from the phenotypic layer and then ran ClinPrior again for the 66,800 synthetic exomes cited above (Fig. 2B). In these conditions, the algorithm prioritized variants worse because the specific phenotypic information of the candidate gene had been removed from the network, thus simulating unknown genes not associated with the phenotypes. In this scenario, the phenotypic score of the candidate gene is obtained only using the network score propagation from adjacent disease genes in the interactome, resulting in an AUROC value of 0.7824, or 0.6489 when using random HPOs (Fig. 2A).

### ClinPrior validation on a prospective, real-world discovery cohort of 135 HSP/CA families

#### Clinical data

**Fig. 3:**
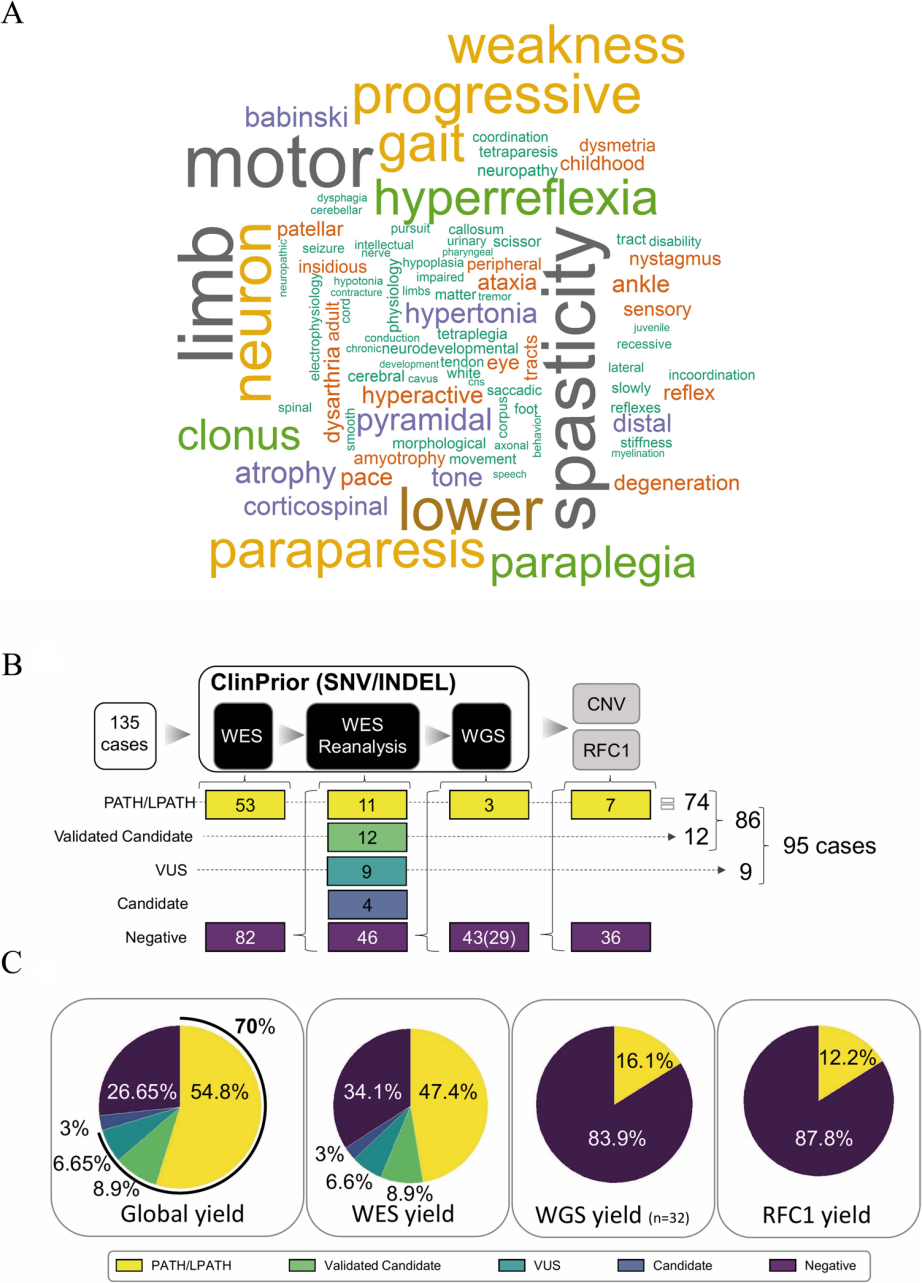
Diagnostic process diagram and diagnostic yield in a patient real-world cohort. **A** Word cloud showing the most representative phenotypes in the 135 patients. **B** Number of cases included in the study and diagnostic process with **C** the diagnostic yield in global, WES, WGS (including CNVs) and RFC1 analysis

We enrolled 135 families with undiagnosed HSP and/or CA (Fig. 3A) after targeted screening for the most common genetic causes, as described in the “ Methods.” The clinical characteristics, studies performed and WES-WGS results of every patient are summarized in Table 1 and Additional file 1: Table S2.

**Table 1:** Main clinical features

Characteristics	Index cases	(%)
Sex		
Female	50	37%
Male	85	63%
Age at onset		
Child onset	85	63%
Adult onset	50	37%
Familial history		
Sporadic	84	62%
Familial	51	38%
Consanguinity	16	12%
**Main clinical features**		
Pure spastic paraplegia	22	16%
Pure cerebellar ataxia	3	2%
Spastic paraplegia /ataxia spectrum	110	81%
Spasticity or ataxia plus other symptoms		
Neuropathy / lower motor neuron	38	29%
Extrapyramidal symptoms	18	14%
White matter involvement	29	21%
Seizures	14	11%
Cognitive impairment	58	43%
**Complementary exams**		
MRI	83	62%
Metabolic assessment	52	38%
Targeted genetic studies ^a^	85	63%
Karyotype / aCGH	15	11%
**TOTAL cases**	**135**	

Baseline characteristics and main clinical features of the ataxia / spastic paraplegia Cohort ^a^ Targeted sequencing or repeat expansion analysis for spinocerebellar ataxias, information available

#### Diagnostic yield of WES and WGS in the HSP/CA real-world discovery cohort

All patients underwent initial WES analysis and were analysed using ClinPrior. At first, the diagnostic rate counting only pathogenic and likely pathogenic variants was 53/135 (39%), which increased to 60/135 (44.4%) after subsequent reanalysis at 12 and 24 months, thus confirming the previously reported importance of case reanalysis [33, 34]. The reanalysis included novel interactomes, novel disease associations and an improved variant calling procedure. In addition, 4 VUS cases were functionally validated, which increased to 64/135 (47%). We also performed functional studies on candidate genes not previously associated with disease (validated candidates), thus achieving the diagnosis of 12 more families, increasing the diagnostic yield to 76/135 (56%). In 9 additional cases, we identified variants of unknown significance (VUS) in genes that were highly compatible with the clinical picture and segregation but were not amenable to experimental validation. We considered these cases to be solved by expert assessment, and the diagnostic yield increased to 85/135 (63%) (Fig. 3B, C).

Next, we performed singleton WGS in 32 of the remaining 46 negative cases, which we prioritized according to the availability of DNA from the proband and parents. We obtained a positive result for five additional families of which three harboured a SNV variant in the *SPG7* [35], *SPTBN2* or *SPTAN1* gene and two harboured a copy-number variant (CNV) in the *SPAST* gene (IDSPG132) of dominant inheritance (2p22.3(32337285–32350543) × 1) or in the *FARS2* gene (6p25.1(5172693–5459957) × 1) in compound heterozygosity with a missense variant (IDSPG116), reaching a final diagnostic yield of 90/135 (67%) (Fig. 3B, C; Additional file 1: Results, Table S3 and Table S4).

**Fig. 4:**
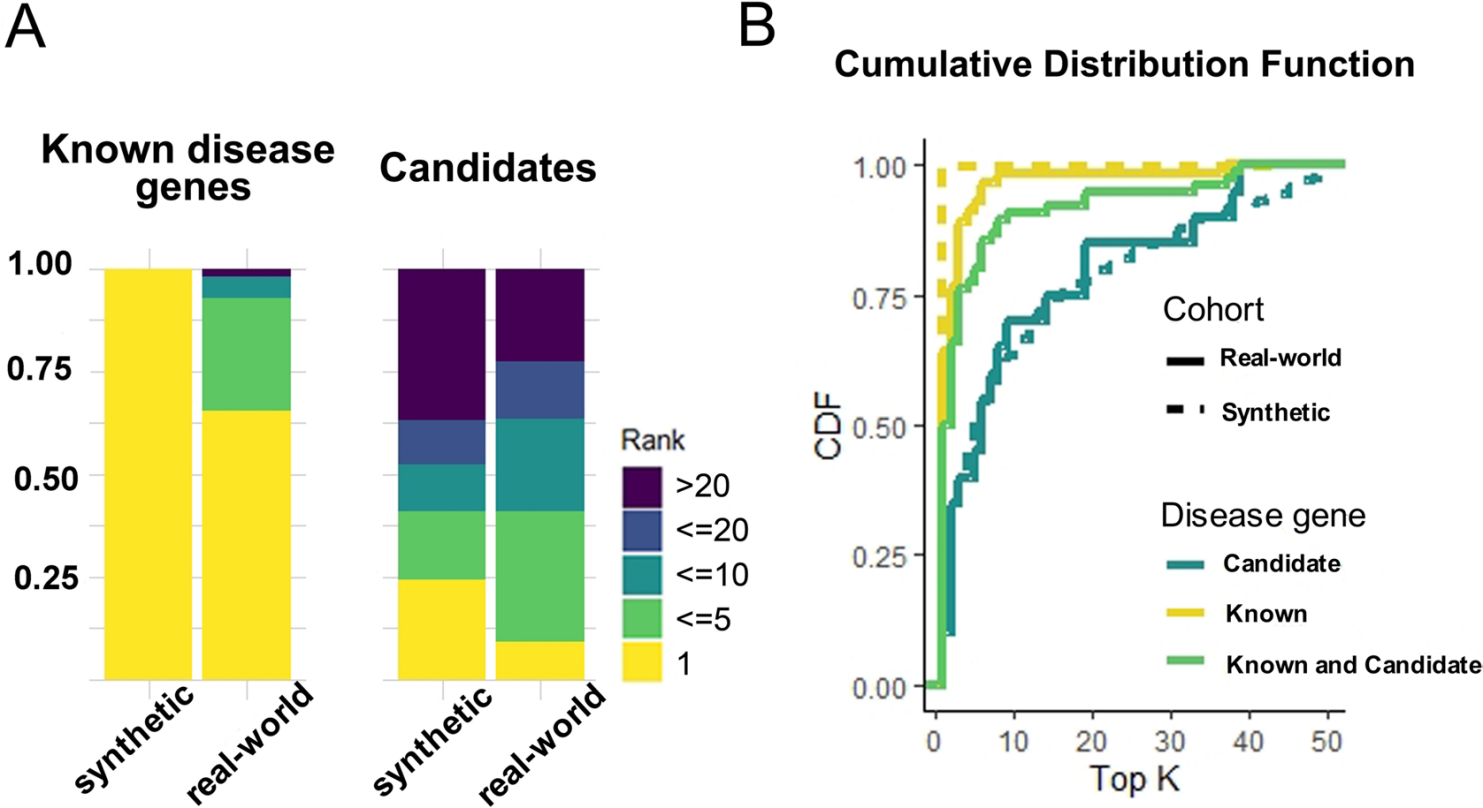
ClinPrior performance yield. ClinPrior performance yield in prioritizing 66,800 pathogenic variants and in a real-world patient cohort including 79 variants in known disease genes or candidates using bar plots (**A**) and CDF plots (**B**)

We examined the performance of ClinPrior in the 76 WES and 3 WGS cases where it was instrumental to provide a direct diagnosis (presence of pathogenic or likely pathogenic SNV/INDEL variants after current American College of Medical Genetics and Genomics (ACMG) criteria). ClinPrior identified 93% of pathogenic/likely pathogenic variants in known disease genes ranked in the top 5 (53/57), of which 65% of variants (37/57) ranked in the top 1. However, in cases with causative variants in strong candidate genes not previously associated with disease and in cases with new phenotypes associated with known genes (both groups computed together), the causative variants ranked in the top 10 in 64% of the cases (14/22) and ranked in the top 5 in 41% of the cases (9/22), as seen on the bar plots (Fig. 4A). In comparison, in the synthetic cohort of 66,800 cases, ClinPrior identified the causative variants as first ranked in 99.8% of cases with known disease genes, as expected. When the variant was found in candidate genes, ClinPrior ranked it in the top 5 in 41% of cases, similar to the real-world cohort (Fig. 4A).

To compare the performance of ClinPrior with other existing tools, we ran Exomiser v.2302/cli-13.2.0 [36] and evaluated the prioritization of the 79 diagnosed cases with pathogenic/likely pathogenic variants from the real-world cohort, yielding 60/79 (75.9%) for the top 5 in Exomiser and 62/79 (78.5%) for ClinPrior. However, it should be noted that in this analysis, Exomiser takes advantage of prior information that ClinPrior did not have at the start of this project. Some of the new phenotypes/candidate genes reported in this paper have been previously published by our group in international peer-reviewed journals, and Exomiser incorporates this information as it regularly updates the HPO disease gene entries. Therefore, Exomiser results are significantly higher than they should be. Nevertheless, ClinPrior’s results still outperform Exomiser, even when the HPO terms associated with each candidate gene/new phenotype-associated gene are removed. To make a fair comparison and given that we were unable to remove HPO disease gene entries from Exomiser, we reclassified the new phenotype and candidate genes previously published by our group (*GFAP* [37], *PI4KA* [38], *SHMT2* [39], *PCYT2* [40], *UBAP1* [41], *DLG4* [42], and *KCNA1* [43]) as known disease genes. Applying these changes, Exomiser prioritizes 56/65 variants (86.2%) in the top 5 for known genes, while ClinPrior prioritizes 57/65 (87.7%); for previously disease-unrelated genes and atypical phenotypes, Exomiser ranks 4/14 variants (28.6%) in the top 10, while ClinPrior gives 9/14 (64.3%). Therefore, while comparing ClinPrior and Exomiser in similar conditions, we conclude that their yield is similar for genes already associated to disease. However, ClinPrior clearly outperforms Exomiser when prioritizing variants in genes not yet associated to disease or with aa atypical phenotype.

We also compared the performance of ClinPrior in both the synthetic and real-world cohorts in the cumulative distribution function (CDF) (Fig. 4B). While the bar plot shows the relative proportion of cases with causal genes ranked within a discrete designated range, the CDF display illustrates the percentage of cases with causal variants ranked within the top K (range between 1 and 50) by each analysis in a continuous way [4]. As expected, we observed that ClinPrior better prioritizes the causal variants of the synthetic cohort compared with the real-world cohort. Within the real-world cohort, ClinPrior performs better when causative variants are found in known genes compared with the variants in candidate genes and novel phenotypes associated for the first time with described disease genes (Fig. 4B). These results provide evidence of the ability of ClinPrior to identify novel disease genes through the combination of a phenotype-driven propagation network and a variant deleteriousness score.

### Discovery of novel disease-causing genes through ClinPrior

This approach allowed us to identify 14 novel candidate genes, for which we gathered additional international cases with compatible phenotypes through the platform GeneMatcher [44]. We functionally validated and reported seven novel disease-causing genes (*SHMT2* [39], *UBAP1* [41], *PI4KA* [38], *PCYT2* [40], *SLC35B2* [45], *SVBP* (Launay et al., under review), and *DLG4* [42]) (Table 2), with three additional novel disease genes undergoing functional characterization, confirmed through three or more international additional families via GeneMatcher. Four more candidate genes are awaiting confirmation through the analysis of additional patients, while functional studies are ongoing.

**Table 2:** Novel genes

Gene	Gene name	ID	Inheritance	Nomenclature	Familial/ sporadic	Age at onset^a^	Spastic paraparesis	Ataxia		Additional features	Validation strategy
*SVBP*	Small vasohibin-binding protein	IDSPG8 IDSPG46	Homozygous Homozygous	p.(Leu49Pro) p.(Leu49Pro)	Familial Familial	162	YesNo	NoNo	Intellectual disability Axonal sensorimotor peripheral neuropathy		Transfection assay, Western blot and Immunofluorescence
*PI4KA*	Phosphatidylinositol 4-kinase, catalytic, alpha	IDSPG16 IDSPG149	Compound Heterozygous Compound Heterozygous	p.(Thr2053SerfsTer4)/ p.(Glu1820del) p.(Val1556Met)/ p.(Thr1720Ile)	Sporadic Sporadic	173	YesYes	NoNo	No Learning difficulty		Targeted lipidomics, Western blot and Immunofluorescence [38]
*SHMT2*	Serine hydroxymethyl transferase, mitochondrial	IDSPG26	Homozygous	p.(Pro499Ala)	Familial	1	Yes	No	Global developmental delay, hypoplastic corpus callosum		Targeted metabolomics and mitochondrial redox metabolism [39]
*PCYT2*	Phosphate cytidylyltransferase 2, ethanolamine	IDSPG27	Homozygous	p.(Lys319Asn)	Sporadic	19	Yes	Yes	Distal hereditary motor neuropathy		Targeted lipidomics and cDNA analysis [40]
*UBAP1*	Ubiquitin-associated protein 1	IDSPG76	Heterozygous	p.(Phe159Ter)	Familial	7	Yes	No	Attention deficit hyperactivity disorder		Truncating variant published with 4 additional families, 3 affected relatives [41]
*DLG4*	Discs large Maguk scaffold protein 4	IDSPG109	Heterozygous	c.1721-1G > A	Sporadic	1	Yes	Yes	Learning difficulty, bradykinesia, dystonia, myoclonus		Truncating variant published with 52 additional families [42]
*SLC35B2*	Solute carrier family 35 (3-prime-phosphoadenosine 5-prime-phosphosulfate transporter), Member B2	IDLNF68	Homozygous	c.1224_1225delAG	Sporadic	0	Yes	No	Intellectual disability		Truncating variant, validated through qRT-PCR, WB, transfection assay [45]

Validated candidate genes, clinical features of affected individuals

^a^ Age in years

**Fig. 5:**
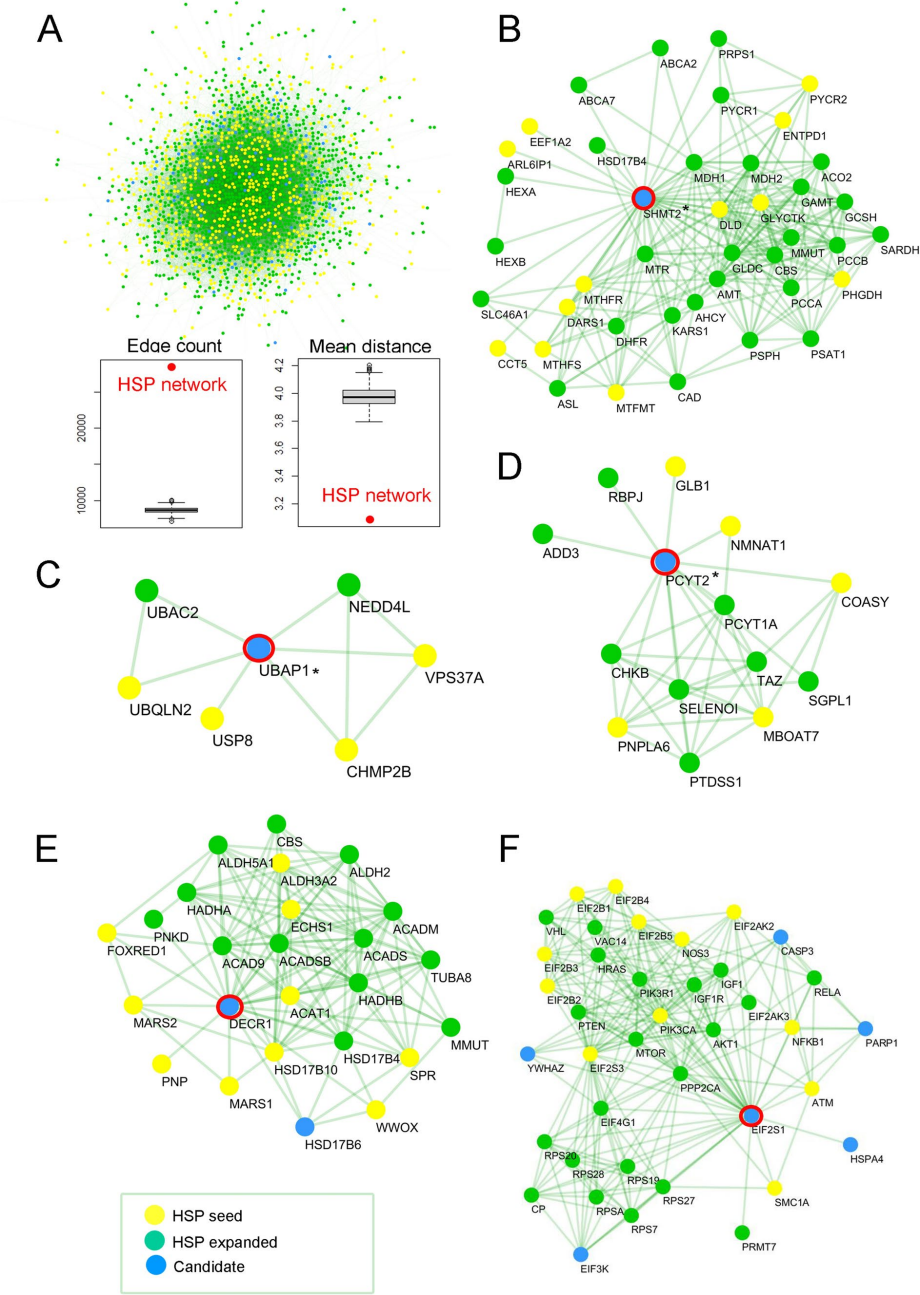
HSP/CA expanded interactome. **A** The HSP/CA seeds + expanded network was generated by the network prioritization tool, resulting in 2187 proteins. The seed genes known to be mutated in HSP/CA are shown in yellow circles, disease genes not previously associated with HSP/CA are shown in green, and new HSP/CA candidate genes are shown in blue. Comparison of the statistical connectivity strength of the HSP/CA expanded network with 1000 permutations of randomly selected proteins from the global human network. Red dots denote the value of the metric on the HSP/CA expanded network constituted by 2187 proteins. Box and whisker plots denote matched null distributions (i.e. 1000 permutations). (Left) Within-group edge count (i.e. number of edges between members of the query set). (Right) Mean distance is the average path length in the network obtained by calculating the shortest paths between all pairs of proteins. **B**–**F** Zoom-in on the network for specific putative candidates as illustrative examples of the potential of the HSP/CA expanded network: **B** serine hydroxymethyltransferase 2 (*SHMT2*); **C** ubiquitin-associated protein 1 (*UBAP1*); **D** phosphate cytidylyltransferase 2, ethanolamine (*PCYT2*); **E** p2,4-dienoyl-CoA reductase 1 (*DECR1*); and **F** eukaryotic translation initiation Factor 2 subunit alpha (*EIF2S1*). * Indicates recently associated with HSP/CA

A paradigmatic example of how our algorithm identifies novel candidate genes through interactome network connections is the recently described *SHMT2* gene, which we identified and functionally validated in 2020 [39]. The mutation in *SHMT2* in patient IDSPG26 was well prioritized in rank 6 in a variant call format (VCF) file with 1595 variants because this gene interacts functionally with several one-carbon metabolism pathway enzymes (*MTFMT*, *MTHFR* or *MTHFS*), which are associated with diseases overlapping phenotypically with our index *SHMT2*-mutated patient. The same occurred with a *PCYT2* mutation in patient IDSPG27 [40], ranked 3 in a VCF file with 1738 variants, because PCYT2 protein interacts with other proteins associated with spastic paraplegia such as PNPLA6 or COASY (Fig. 5).

#### RFC1 expansion analysis

During our study, two reports identified biallelic pentanucleotide AAGGG intronic expansion of 400 to 2000 repeats in the *RFC1* gene in patients affected by CA, neuropathy and vestibular areflexia syndrome (CANVAS) [46, 47]. Because this is a relatively frequent expansion causing up to 14% of adult sporadic ataxias [48], we investigated our patients with compatible phenotypes. We thus applied a combination of repeat-primed PCR (RP-PCR) targeting the AAGGG repeat unit and standard flanking PCR as described [47] on the remaining 41 negative cases. We detected the presence of a biallelic mutated AAGGG repeat expansion in 5/41 patients (12%). One of the patients had previously been diagnosed with idiopathic late-onset CA (ILOCA), three had been diagnosed with CA and axonal sensory neuropathy, and only one exhibited a full CANVAS phenotype. Our results support the high prevalence of biallelic expansion of RFC1 in this clinical spectrum.

By adding this additional diagnosis through *RFC1* repeat expansion to the previous data, we obtained a total positive genetic diagnosis in 86 out of 135 HSP/ataxia cases (64%), which increased to 95 (70%) when considering the phenotypically compatible VUS variants **(**Fig. 4B and 4C; Additional file 1: Tables S2 and S5, and “ Methods”).

#### Genetic findings in the HSP/CA real-world discovery cohort

While our final diagnostic rate was 70% (95/135), the diagnostic rate by onset age was 75% (64/85) in those with paediatric onset (< 20 years) and 62% (31/50) in those with adult onset (≥ 20 years). There were no significant differences in yield between the 84 sporadic cases (71%, 60/84) and the 51 familial cases (69%, 35/51). Considering the clinical pattern, the diagnostic rate was 15/22 (68%) in the pure HSP group, 1/3 (33%) in the pure CA group and 79/110 (70%) in the HSP/CA spectrum group.

Although the genetic heterogeneity in our cohort was very high, some genes were found to be more frequently mutated, including *POLR3A*, *SPG11* and RFC1 (*n* = 5 each), *BSCL2*, *SPAST* and *SPG7* (*n* = 4 each), and *ATL1* and *KIF1A* (*n* = 3 each) (Additional file 1: Table S6). New phenotypes were identified in three cases (*LONP1*, *PDK3* and *SPTAN1*), a new inheritance mode and associated novel phenotypes were identified in two patients (a biallelic variant in *KCNA1* [43] and heterozygous variant in *SARS1* [49]), and atypical forms of presentation were identified for six genes (*GFAP* [37], *NDUFS6*, *ACER3*, *KIDINS220*, *COL6A3* and *PMM2*) (Additional file 1: Table S3). Finally, four patients had complex, blended phenotypes associated with variants in more than one gene (Additional file 1: Results).

Among the 95 cases diagnosed, 56 harboured biallelic variants (34 homozygous variants; 16 of them in reported consanguineous families), 36 showed an autosomal-dominant mode of inheritance (11 de novo) and 3 cases were caused by mutation in an X-linked gene. Moreover, we identified two uniparental disomy events of maternal origin: one event was observed on chromosome 16 in patient IDSPG10, who harboured a nonsynonymous single-nucleotide variant in the *FA2H* gene [50, 51]; the other event occurred on chromosome 6 in patient IDLNF68 with a frameshift deletion variant in the *SLC35B2* gene [45]. Segregation by Sanger sequencing was performed in all but 10 patients due to the unavailability of parental samples. We found several variants more than once in our patients: (i) the *BSCL2* p.(Asn88Ser) variant was found in four independent families from a small region of the Basque Country coast, suggesting a founder effect (frequency: 0.000001591 in gnomAD (v2.1.1) [52]; (ii) the deep intronic *POLR3A* c.1909 + 22G > A variant (frequency: 0.001364 in gnomAD (v2.1.1)) was found in 3 families, and it had previously been identified as a frequent cause of hereditary spastic ataxia [53]; (iii) the *IRF2BPL* p.(Arg188Ter) variant (frequency: 0.000000433 in gnomAD (v2.1.1)) was found in two independent families [54], and (iv) the *SVBP* p.(Leu49Pro) variant was found twice independently (frequency: 0.0000019 in gnomAD (v2.1.1)) (Launay et al., under review). We identified only two CNVs in this series in *SPAST* and *FARS2*, most likely because the most frequent CNVs were excluded by candidate-gene testing prior to WES. An added value of our study is that 36 of the 103 identified variants had not been previously reported in the literature, the Human Gene Mutation Database (HGMD, public access), or the ClinVar database (Table 3). The transfer of these novel variants to the ClinVar database is planned.

**Table 3:** New variants (not described previously in literature)

Gene	Patient	Inheritance	Type	Nomenclature	Classification ACMG
*ACER3*	*IDSPG75*	AR	Missense	NP_060837.3:p.(Gly211Cys)	Pathogenic
*AMPD2*	*IDSPG78*	AR	Frameshift deletion	NP_631895.1:p.(Ala62SerfsTer40)	Pathogenic
*BCKDK*	*IDSPG47.0*	AR	Missense	NP_005872.2:p.(Arg327Trp)	Likely pathogenic
*CAPN10*	*IDSPG47.1*	AR	Splicing	NM_023083.3:c.1989 + 1G > A	Pathogenic
*DLG4*	*IDSPG107*	AD	Splicing	NM_001365.4:c.1721-1G > A	Pathogenic
*ERBB4*	*IDSPG38*	AD	Non-canonical splicing	NM_005235.2:c.2487 + 8_2487 + 11dell	VUS
*FA2H*	*IDSPG10*	AR	Missense	NP_077282.3:p.(Lys262Thr)	Likely pathogenic
*GFAP*	*IDSPG4*	AD	Missense	NP_001124491.1:p.(Gly18Val)	Pathogenic
*IFIH1*	*IDSPG3*	AD	Missense	NP_071451.2:p.(Leu320Phe)	VUS
*KCNA1*	*IDLNF52*	AR	Missense	NP_000208.2:p.(Val368Leu))	Pathogenic
*KIDINS220*	*IDSPG118*	AD	Splicing	NM_020738.3:c.4054-1G > C	Likely pathogenic
*KIF5A*	*IDSPG17*	AD	Missense	NP_004975.2:p.(Gly246Val)	Pathogenic
*KMT2B*	*IDSPG114*	AD	Missense	NP_055542.1:p.(Ala1727Ser)	Likely pathogenic
*LAMA1*	*IDSPG56*	AR	Frameshift insertion	NP_005550.2:p.(Gly2899GlufsTer18)	Pathogenic
*LAMA1*	*IDSPG56*	AR	Non-canonical splicing	NM_005559.3:c.1423-12C > G	Pathogenic
*LONP1*	*IDSPG166*	AR	Splicing	NM_004793.3:c.2154 + 1G > C	Pathogenic
*LONP1*	*IDSPG166*	AR	Missense	NP_004784.2:p.(Leu306Trp)	Likely pathogenic
*PCYT2*	*IDSPG27*	AR	Missense, splicing	NP_001171846.1:p.(Lys319Asn)	Likely pathogenic
*PI4KA*	*IDSPG16*	AR	Frameshift deletion	NP_477352.3:p.(Thr2053SerfsTer4)	Pathogenic
*PI4KA*	*IDSPG16*	AR	Frameshift deletion	NP_477352.3:p.(Glu1820del)	Pathogenic
*PI4KA*	*IDSPG149*	AR	Missense	NP_477352.3:p.(Val1556Met)	Pathogenic
*PI4KA*	*IDSPG149*	AR	Missense	NP_477352.3:p.(Thr1720Ile)	Pathogenic
*PNPLA6*	*IDSPG13*	AR	Splicing	NM_006702.4:c.598-2A > C	Pathogenic
*PNPLA6*	*IDSPG13*	AR	Missense	NP_001159586.1:p.(Ser1138Cys)	Likely pathogenic
*POLG*	*IDSPG113*	AR	Non-canonical splicing	NM_002693.2:c.2266-64C > T	VUS
*POLR3B*	*IDSPG66*	AD	Missense	NP_060552.4:p.(Ala69Gly)	VUS
*REEP1*	*IDSPG12*	AD	Missense	NP_075063.1:p.(Leu59His)	Likely pathogenic
*SARS1*	*IDSPG64*	AD	Splicing	NM_006513.4:c.969 + 1_969 + 3del	Pathogenic
*SHMT2*	*IDSPG26*	AR	Missense	NP_005403.2:p.(Pro499Ala)	Likely pathogenic
*SLC35B2*	*IDLNF68*	AR	Frameshift deletion	NP_835361.1:p.Arg408SerfsTer18	Pathogenic
*SPG7*	*IDSPG23*	AR	Non frameshift deletion	NP_003110.1:p.(Val311del)	Likely pathogenic
*SPG7*	*IDSPG30*	AR	Missense	NP_003110.1:p.(Met667Ile))	Likely pathogenic
*SPTBN2*	*IDSPG125*	AD	Frameshift deletion	NP_008877.1:p.(Asp1861ThrfsTer59)	Pathogenic
*SVBP*	*IDSPG8,IDSPG46*	AR	Missense	NP_955374.1:p.(Leu49Pro)	Likely pathogenic
*TAF1*	*IDSPG71*	XL	Missense	NP_001273003.1:p.(Ala1732Ser)	VUS
*UBAP1*	*IDSPG76*	AD	Frameshift deletion	NP_057609.2:p.(Phe159Ter)	Pathogenic

*AD* autosomal dominant, *AR* autosomal recessive, *SNV* single-nucleotide variant, *VUS* variant of unknown significance

List with the 36 SNV/INDEL new variants identified in our cohort, classification according to ACMG criteria

#### Management implications of a positive genetic diagnosis

Importantly, establishing the genetic diagnosis allowed us to improve the clinical management of 10 patients (Additional file 1: Table S2). In 3 of these patients, the genetic diagnosis led to the consideration of a specific treatment option for the disease, for instance, changes in dietary management for a patient with branched-chain ketoacid dehydrogenase kinase deficiency caused by *BCKDK* pathogenic variants (OMIM # 614,923); or ameliorated seizure management by adding a sodium channel blocker (oxcarbazepine) for a patient with epileptic encephalopathy caused by a *KCNA1* variant (IDLNF52), which markedly improved seizure control [43].

Finally, we identified and reported incidental findings after current ACMG guidelines in five patients across four genes: *MYBPC3* (p.Asn1023GlnfsTer28) in IDSPG103, *PKP2* (p.Leu92Ter) in IDSPG170, *DSC2* (c.1664-1G > A) in IDSPG149 and (p.Arg375Ter) in IDSPG47.0 and *PMS2* (p.Arg287SerfsTer19) in IDSPG3.5. The first four patients underwent cardiological surveillance, whereas the fifth patient was referred to a specialized Cancer Genetics Risk Assessment and Counselling unit.

#### Experimental validation of variants of unknown significance

According to the ACMG and the Association for Molecular Pathology (AMP) guidelines [55–57], 86 cases were classified as definitively diagnosed with pathogenic or likely pathogenic variants. To validate the pathogenic role of VUSs, we performed several functional assays. We evaluated the impact of 6 variants on splicing using either a minigene splicing assay and/or fibroblast or peripheral blood mononuclear cell cDNA sequencing of the *SPG7* [35], *LAMA1*, *KIDINS220* and *SEPSECS* genes. Targeted quantitative lipidomics studies confirmed a pathogenic role for variants in genes associated with lipid metabolism disorders, such as *PI4KA* [38]*, PCYT2* [40] and *ACER3*, in *n* = 4 cases. Targeted metabolomics and mitochondrial respiration assays showed a significant impairment of amino acid and folate metabolism and mitochondrial energy production, key pathways catalysed by the enzyme serine hydroxymethyltransferase encoded by *SHMT2* [39]. A patch-clamp assay to measure potassium currents allowed us to confirm the pathogenic loss-of-function (LoF) role of a homozygous variant in *KCNA1* (p.Val368Leu), unveiling a novel inheritance mode for the disorder [43]. The SARS1 variant was functionally confirmed using serylation assays and yeast complementation studies [49], and SLC35B2 variant pathogenicity was confirmed by mRNA and protein quantification, together with immunofluorescence analysis. These analyses, together with quantitative real-time (qRT‒PCR) for CNV validation in *FARS2* and *SPAST* genes, Western blots, or immunofluorescence when needed, identified a deleterious effect for 18 variants, which enabled us to classify these variants as pathogenic (Additional file 1: Table S5 and S7).

### ClinPrior generates an expanded network for novel disease gene discovery in HSP/CA

Based on the principle that physically and functionally interacting genes may account for related biological processes and cause similar diseases, we decided to generate an HSP/ataxia-specific interactome (Fig. 5A) [58]. We started with an initial list of 718 seed genes causing or associated with HSP/CA that were identified as having the terms “spastic paraplegia” or “ataxia” in HPOs included in the OMIM database. Next, we used ClinPrior to obtain a list of the top 1000 prioritized genes for each seed gene after considering the HPO-gene associations of the 718 genes as the patient clinical features. With the most recurrent genes present among the 718 lists, we obtained 2187 genes that we extracted from the global physical and functional ClinPrior networks, resulting in a final HSP/CA expanded interactome of 27,759 gene‒gene interactions. To assess whether there was greater connectivity in the HSP/CA expanded network than in the global network, we compared the number of connections and the average path length between all node pairs with a 1000 randomly selected set of 2187 genes derived from the global network. We determined that the HSP/CA expanded network was significantly more cohesive than expected by chance (*p* < 1E − 25).

To evaluate the functional signature of these 2187 proteins, we performed an enrichment analysis of the Gene Ontology (GO) terms (Additional file 1: Tables S8-S10). In line with our hypothesis that genes associated with similar diseases may converge towards common biological pathways, major modules that have previously been linked to HSP/ataxia pathophysiology emerged from our analysis: (i) anterograde transsynaptic signalling (e.g. the SPG11 vesicle trafficking-associated protein spatacsin); (ii) microtubule binding (e.g. spastin, *SPAST*); (iii) the mitochondrial oxidative phosphorylation (OXPHOS) system (e.g. NADH ubiquinone oxidoreductase Fe-S protein 1, *NDUFS1*); (iv) aminoacyl-tRNA ligase activity (e.g. aspartyl-tRNA synthetase 1, *DARS1*); and (v) the peroxisome biogenesis and metabolic network (e.g. peroxin 16, *PEX16*).

Among the 2187 genes conforming to this network, we can highlight 3 groups of genes: (i) 718 genes that were used as seed genes due to their direct association with HSP/CA, (ii) 1394 that have previously been associated with rare diseases (but have not yet been associated with HSP/ataxia), and (iii) 75 novel candidate genes that were not previously associated with HSP/ataxia or any other disease (Additional file 1: Table S11). Among these 75 new candidates, we found 17 genes predicted to be extremely intolerant to loss-of-function (pLI ≥ 0.9) and 8 genes strongly intolerant to missense variation (*Z* score ≥ 3.08) (i.e., with probability *p* < 0.001). This last list of 75 genes can be instrumental in identifying the causative mutations in undiagnosed patients, for which the mutated gene is not yet associated with disease, such as (1) *DECR1* (22,4-dienoyl-CoA reductase 1) (Fig. 5E) and (2) *EIF2S1* (eukaryotic translation initiation Factor 2 subunit alpha) (Fig. 5F; Additional file 1: Table S11). Our method prioritizes these genes because they are functional interactors of genes with similar functions that cause diseases, such as the fatty acid beta oxidation enzymes *ACAT1* and *ECHS1* and eukaryotic translation initiation factors *EIF2S3*, *EIF2AK2*, *EIF2B4*, or *EIF2B5*, respectively. In the candidate list, we found novel disease genes already identified in our HSP/CA patient cohort, such as (i) *SHMT2* (OMIM #619121), mutated in patient IDSPG26 [39] Fig. 5B; (ii) *PI4KA (OMIM # 616531)*, mutated in patients IDSPG16 and IDSPG149 [38]; (iii) *UBAP1*, (OMIM #618418), mutated in patient IDSPG76 [41] Fig. 5C; (iv) *PCYT2* (OMIM # 618770), mutated in patient IDSPG27 [40] Fig. 5D; and (v) *DLG4* (OMIM # 18793), mutated in patient IDSPG107 [42].

## Discussion

Recent variant prioritization tools have demonstrated efficacy, albeit only a handful of them have been thoroughly validated with real-world cohorts, and none have been validated to identify novel disease-gene associations [3, 4]. Here, we present a phenotype-driven computational tool to aid with clinical correlation and variant interpretation based on interactome data and provide proof of efficacy at novel disease-gene discovery. Indeed, when applied to a synthetic cohort of 66,800 WES cases, ClinPrior was able to identify the causative variants in 99.8% of cases with a gene previously associated with disease and in 41% of cases when the causative gene was a novel disease-candidate gene, being the causative gene ranked in the top-5 positions. A similar percentage of 41% was achieved with the real-world prospective cohort of 135 families, thus underscoring the high efficiency of the method to diagnose the most challenging cases.

Overall, ClinPrior facilitated the genetic analysis in a series of 135 families by WES/WGS, enabling the diagnosis of 60 families (44.4%) carrying a pathogenic or likely pathogenic SNV or small INDEL variant, including WES re-analysis at 12 and 24 months with new disease associations and improved variant calling, but without functional validation. The diagnostic yield increases by almost 15% to 79 families (58.5%) when including both functional validation of VUSs that match the clinical phenotype and of VUSs in novel phenotypes/candidate genes. The diagnostic yield reaches 88 families (65%) if we include non-experimentally validated cases with VUS but with compatible segregation studies and specific clinical and MRI findings highly suggestive of the particular gene. Finally, by adding CNV identification and the RFC1 expansion test to all remaining undiagnosed cases, we reached a final diagnostic yield of 70%, which is, to our knowledge, the series presenting the highest diagnostic rate for the HSP/CA spectrum. Indeed, these results are superior to those recently reported in a study that included 260 cases studied by singleton WES, CNV analysis and short tandem repeat expansion analysis, which reached a diagnostic yield of 52% and in which 7% of the diagnoses were obtained after reanalysis [59]. Their results are consistent with those reported in previous studies, where diagnostic yield excluding VUSs ranged between 25 and 55% (mean: 33%) (Additional file 1: Table S1). Altogether, our report adds to the growing body of evidence indicating that WES/WGS provides superior diagnostic yield compared to studies using targeted gene panels or even clinical exomes (between 111 and 6700 genes) [60–63], which solved only 19–46% of the cases. For instance, using PanelApp gene panels to analyse the obtained results from our real-world cohort (see Additional file 1: Methods), we would have diagnosed variants in 64/135 (47.4%) in contrast to ClinPrior’s 79/135 (58.5%) cases solved. Therefore, variants in 15/135 cases (11.11%) would have not been detected by using. Of those 15 missed cases, 6 cases carried variants in our recently validated candidate genes in 2020 (*SHMT2* [39], 2021 (*DLG4* [42], 2022 (*SLC35B2* [45] and publications on progress, explaining absence of these genes from panels. The other 9 missed cases are related to atypical phenotypes or non-classical HSP genes (*L2HGDH*, *NDUFS6*, *SARS1* [49], *KMT2B*, *TRMT5*, *COL6A3*, *LONP1*, *MMUT* and *PDK3*). Moreover, in this cohort, we identified 5 cases with incidental findings, which would have been missed in gene panels, with the consequent negative impact on the proband health. We believe this illustrates main advantages of using WES/WGS over gene panels. It is worth noting that the high diagnostic yield was achieved after reanalysis and functional validation of the VUSs prioritized by the algorithm. Therefore, we believe that functional validation is not a stand-alone approach, but a necessary step to validate the results of the prioritizer.

The number of known genes responsible for human disease has increased exponentially since the advent of NGS-based technologies, with more than ~ 300 novel disease-gene associations being reported annually according to The Mendelian Genomic Research Consortium (https://gregorconsortium.org). The OMIM database reflects between 30 and 60 new entries on novel disease genes or phenotypes and between 300 and 900 updates on known genes per month (https://www.omim.org/statistics/update, December 22, 2022), which underscores the ever-changing genetic diagnostic landscape.

The incomplete knowledge of disease-gene databases, together with the challenge of identifying atypical phenotypes not yet described in the literature, hampers patient diagnosis. This clinical heterogeneity/variable expressivity is frequently encountered in rare diseases, across broad overlapping clinical spectra to very distinct phenotypes [64, 65]. Therefore, the use of tools directed to novel disease entities is needed to optimize diagnostic yields. In this sense, ClinPrior enabled the diagnosis of several cases with atypical presentations (*GFAP* [37], *PMM2*, *PDK3*, *KIDINS220, COL6A3*), expanded the phenotype of recently identified genes (*SPTAN1, NDUFS6, ACER3*), described previously unknown modes of inheritance (*KCNA1* [43] and *SARS1* [49]) characterized families harbouring variants in more than one causative gene with blended phenotypes (i.e., *CACNA1A* and *POLR3A* in the same patient), and, importantly, discovered novel disease entities and their novel causative genes in 16 cases. From these, we functionally validated 9 cases to date (i.e., *PCYT2* [40], *SHMT2* [39], *PI4KA* [38], *UBAP1* [41], *DLG4* [42], *SLC35B2* [45] and *SVBP* (Launay et al., under review)), while others are currently ongoing (Table 2; Additional file 1: Table S2).

Furthermore, in 13 families (9.7%), we identified genes primarily associated with peripheral neuropathies (3 patients: *SLC2A46, PDK3, MORC2*), white matter disorders (3 patients: *RNASEH2B*, *GFAP*, *ACER3*), and neurodevelopmental disorders (5 patients: *IRF2BPL*, *CTNNB1*, *DLG4*, *TAF1* and *SPTAN1*), which would have most likely been missed by gene panels targeting HSP/ataxia genes. These results highlight the notion of a clinical spectrum continuum between HSPs and CAs with these other clinical entities. Consequently, ClinPrior boosted the identification of novel genes responsible for Mendelian diseases and the recognition of clinical heterogeneity in atypical cases, largely surpassing diagnostic yields based on NGS panels and clinical WES.

WES is inefficient at detecting deep intronic variants, structural variants and repeat expansions, which are known to be prevalent in these diseases [47, 66]. Although our cohort was screened for known repeat expansions linked to HSP/CA prior to WES and a posteriori for the repeat expansion in *RFC1* [48], it is possible that some of our negative cases can be explained by novel, undetected structural variants or repeat expansions. Indeed, while this paper was in preparation, a novel intronic expansion in the *FGF14* gene responsible for 10–15% of adult CA cases was reported [67, 68]. Thus, sequencing of the remaining negative cases by WGS, together with upgraded methods for detecting repeat expansions (ExpansionHunter, exSTRa, STRetch, TREDPARSE) [69]) and CNVs, is warranted to solve the remaining negative cases.

Our method and study protocol have certain limitations. The quality and number of patient HPO terms provided to ClinPrior is critical to achieving good results. We recommend annotating the patient phenotype with as many HPO terms as possible, reflecting the entire pathology and not just the most important aspects. To take advantage of the phenotypic prioritization process, we recommend running ClinPrior with at least 7 to 10 specific HPOs (see benchmarking for HPO number optimization in the Supplementary Results). Otherwise, the variant will be prioritized primarily by the variant deleteriousness score. In addition to HSPs, we have also demonstrated the performance of ClinPrior in a large cohort of brain white matter disorders [70], encouraging the testing of this algorithm in other disease entities. Another limitation is the knowledge gaps in phenotypic data and in the multi-layered interactome that is fed by dynamic and changing databases. Therefore, regular updating of HPO-gene associations and gene–gene interactions is critical to achieve better results.

In summary, we provide evidence of the effectiveness of ClinPrior applied to WES/WGS data to diagnose patients with HSP and CA and to identify new phenotypes and novel disease genes. Those inherited disorders display considerable genetic heterogeneity (68 different genes identified among the 95 diagnosed cases) and show an evident genotypic and phenotypic overlap, thus supporting a unified diagnostic approach for considering spastic paraplegias, cerebellar ataxias, peripheral neuropathies and white matter diseases as part of the same continuum.

## Conclusions

The phenotype-driven, interactome-based prioritization algorithm ClinPrior provides an opportunity to accelerate and improve clinical genomic diagnostics yields, along with the recognition of clinical heterogeneity in atypical cases, shortening diagnostic Odysseys and largely surpassing NGS panels and clinical WES. ClinPrior is particularly well suited for boosting the discovery of novel disease-causing genes which allows broadening fundamental knowledge related to human disease. Of note, functional analysis increased diagnostic yield by 15%, underscoring the benefits of integrated functional labs in clinical genomic units.

### Supplementary Information

**Additional file 1.**

## Data Availability

We have uploaded to the Zenodo repository under the following https://doi.org/10.5281/zenodo.7945507: (i) a Pheno-dataset with 82 patients with the causal gene and associated patient HPO terms, (ii) a ClinVar dataset with 66,800 pathogenic variants in a VCF file, (iii) benchmarking data to obtain the optimal number of HPOs required for gene prioritization in 82 patients from the real-world cohort, and (iv) benchmarking data for the prioritization yield of known and candidate disease genes using 66,800 synthetic WES [31]. The ClinPrior package is available at under the following doi: https://zenodo.org/record/7845939 [29] and at https://github.com/aschluter/ClinPrior [28]. The HSP/CA expanded interactome is available in the NDEx repository at [58]: http://www.ndexbio.org/#/network/9a5c7fd0-e61f-11eb-b666-0ac135e8bacf?accesskey=d786cfb7addf9e47df34e3c149d6eb7e3c728a97bcfa8f4676a8dda072365e1c The NGS data supporting the results of this study are available on request from the corresponding author. The data will not be publicly available due to privacy and ethical restrictions as outlined in the signed informed consent form. Genomic data in VCF file format will only be transferred to an accredited institutional researcher after verification that the purpose of the study is for a biomedical research project approved by a local ethics committee duly accredited at a national level. In addition, a Data Transfer Agreement (DTA) must be signed as part of the inter-institutional collaboration.

## Abbreviations

aCGH: Array comparative genomic hybridization
ACMG: American College of Medical Genetics and Genomics
AMP: Association for Molecular Pathology
AUROC: Area under the receiver operating characteristic
CA: Cerebellar ataxia
CANVAS: Cerebellar ataxia, neuropathy and vestibular areflexia syndrome
CDF: Cumulative distribution function
CNV: Copy number variation
GO: Gene Ontology
HGMD: Human Gene Mutation Database
HPO: Human Phenotype Ontology
HSP: Hereditary spastic paraplegia
ILOCA: Idiopathic late-onset cerebellar ataxia
INDEL: Insertion deletion variant
MAF: Minor allele frequency
MRI: Magnetic resonance imaging
NGS: Next-generation sequencing
qRT‒PCR: Real-time quantitative reverse transcription PCR
ROC: Receiver operating characteristic
RP-PCR: Repeat-primed PCR
SNV: Single-nucleotide variant
VCF: Variant call format
VEP: Variant Effect Predictor
VUS: Variant of uncertain significance
WES: Whole-exome sequencing
WGS: Whole-genome sequencing
