# Supplementary material for: ClinPrior: an algorithm for diagnosis and novel gene discovery by network-based prioritization
Source: Genome Med. 2023 Sep 7;15:68. doi: 10.1186/s13073-023-01214-2 (PMC10486091; doi:10.1186/s13073-023-01214-2)
Supplement: Supplementary file 1 — Additional file 1. [file 13073_2023_1214_MOESM1_ESM.pdf]

Additional Methods

- **Benchmarking using a synthetic WES dataset**
- **HSP/CA expanded network**
- **WES/WGS and variant calling**
- **Variant segregation and classification**
- **Functional validation**
- **Detection of the biallelic intronic expansion AAGGG in RFC1**
- **PanelApp panels related to Hereditary Spastic Paraplegia or Ataxia**
- **Receiver Operating Characteristic (ROC)**

Additional Results

- **Illustrative Clinical Cases**
  1. **Atypical phenotypes**
  2. **Novel phenotypes**
  3. **New inheritance mode**
  4. **Cases with dual diagnoses**
- **WGS cases with SNVs**
- **Benchmarking for HPO number optimisation on a real-world cohort**

Additional References

Additional Tables

- **Table S1. NGS diagnostic yield among published ataxia/HSP studies.**
- **Table S2. Clinical description table.**
- **Table S3. Atypical cases.**

- Table S4. Cases with functionally validated CNVs.

- Table S5. ACMG criteria for identified variant classification.

- Table S6. List of identified genes, OMIM nomenclature and numbers of cases identified in our cohort.

- Table S7. Cases with functionally validated variants.

- Table S8. Molecular function GO terms enriched in the HSP/CA network.

- Table S9. Biological process GO terms enriched in the HSP/CA network.

- Table S10. Cellular compartment GO terms enriched in the HSP/CA network.

- Table S11. Candidate genes in the HSP/CA expanded network.

Additional Methods

Benchmarking using a synthetic WES dataset

To test the performance of the method at identifying causal variants in sequencing data, we generated a synthetic whole exome sequencing (WES) dataset consisting of 68,210 VCF files (one for each of the pathogenic variants retrieved from the ClinVar December 2019 release). We excluded variants classified as “modifier” and in noncoding regions and those genes with HPO-gene associations below 5, resulting in 66,800 variants [1]. The synthetic dataset was created by inserting a single pathogenic variant from the set of 66,800 in high-confidence variant calls for one individual (NA12878) published by the Genome in a Bottle (GIAB) consortium, used frequently for benchmarking purposes [2]. Each pathogenic variant zygosity was adjusted to match the OMIM disease’s mode of inheritance for the associated gene (heterozygous for dominant diseases, homo or hemizygous for recessive diseases). Synthetic exomes were then filtered based on frequency (MAF<0.1% according to the VEP filter tool in each of the 1000 Genomes Project, NHLBI GO Exome Sequencing Project (ESP), ExAC and gnomAD) and variant impact excluding noncoding regions and modifier ones, resulting in 642 variants. We then ran ClinPrior using as an input 1) the filtered VCF files and 2) the patient phenotypic features created with the HPOs of the pathogenic variant-associated gene extracted from the HPO-gene associations from the phenotypic layer.

The prioritization process in ClinPrior is weighted by two variables: i) calculation of the phenotypic association metric derived from prior phenotype-gene association knowledge and further network propagation of this metric, and ii) calculation of the variant deleteriousness

score. Using the synthetic data from 66,800 WES, we consider four different benchmarking scenarios:

Scenario 1.

- Causal gene known (prior knowledge).
- Patient with perfect phenotypic match (all HPOs associated to the gene).

Scenario 2.

- Causal gene known (prior knowledge).
- Patient with unmatching phenotypic data (random HPOs).

Scenario 3.

- Novel candidate causal gene (removed candidate HPOs-gene associations from the 439.300 HPO-gene associations, therefore without prior knowledge)
- Patient with perfect phenotypic match (all HPOs associated to the gene).

Scenario 4.

- Novel candidate causal gene (removed candidate HPOs-gene associations from the 439.300 HPO-gene associations, therefore without prior knowledge)
- Patient with unmatching phenotypic data (random HPOs).

In the first scenario 1, we considered an ideal situation where the simulated patient's phenotype perfectly matches the clinical description from the databases associated with the causal gene. Here, the phenotypic association score is the highest possible, resulting in the desired near-perfect **top-1** identification (AUROC = 0.9994). This perfect scenario is in contrast with the rest: i) Scenario 2, with random phenotypic data, where patient phenotypic mismatch diminishes the phenotypic association metric, and the prioritization is driven mostly by the variant deleteriousness score (AUROC = 0.8393); ii) Scenario 3, with simulated novel candidate genes, removing prior HPO-gene associations of the candidate, but

with perfect patient phenotypic matching. Here, both the phenotypic metric propagation and the variant deleteriousness score are the main drivers of variant ranking (AUROC = 0.7824); and iii) Scenario 4, or worse-case scenario, with simulated novel candidate genes but with unmatched (random) patient phenotypic data. Here, only the variant deleteriousness score is the main driver of variant prioritization (AUROC=0.6489).

HSP/CA expanded network

We constructed an HSP/CA expanded network with an initial list of 718 seed genes with the terms "spastic paraplegia" or "ataxia" in HPOs included in the OMIM database. Next, we used ClinPrior to obtain a list of the 1,000 prioritized genes for each seed gene after considering the HPO-gene associations of the 718-gene from the phenotypic layer as the patient. Then, we selected the most recurrent genes, with at least 75 appearances, among the 718 lists with the top 1,000 prioritized genes. With this procedure, we obtained 2,187 genes that we extracted from the global physical and functional ClinPrior networks, resulting in a final HSP/CA expanded interactome of 27,759 gene–gene interactions. Network available in the NDEx repository [3].

To assess whether there was greater connectivity in an HSP/CA expanded network than in the global network, we calculated i) the number of edges between protein pairs and ii) the average path length in the HSP/CA network by calculating the shortest paths between all protein pairs. We then compared these statistics for 1,000 permutations of a randomly selected set of 2,187 proteins derived from the global network. Finally, we calculated the Z scores to determine how far the measures of the HSP/CA expanded network deviate from the expected mean (μ).

To evaluate which pathways or functional categories were enriched in the HSP/CA network, we followed a similar strategy as described elsewhere [4]. Briefly, we used hypergeometric-based tests from the GOstats package [5]. We used $p < 0.001$ as the cut-off point for GO terms with fewer than 1,000 protein members to determine which GO terms were significantly enriched.

WES-WGS and variant calling

For WES analysis, capture was performed using the SeqCap EZ Human Exome Kit v3.0 (Roche Nimblegen, USA) or the SureSelect XT Human All Exon V5 50 Mb kit (Agilent, USA) with 100-bp paired-end read sequences, and for WGS, a PCR-free library with 150-bp paired-end read sequences was generated on a HiSeq 2000-4000 platform (Illumina, Inc. USA) at Centre Nacional d'Anàlisi Genòmica (CNAG Barcelona, Spain). Sequences were aligned to hg19 by Burrows–Wheeler Aligner (BWA mem), and single nucleotide variants and small insertions/deletions (indels) were identified using GATK, applying GATK's best practices for germline single nucleotide polymorphism (SNP) and indel discovery in WES [6]. Structural variants, including CNVs, were called using ClinSV v0.9[7]. CNVs for genome data were detected by ControlFreeC v11.5 [8,9] using a window size of 20 kb and a step size of 4 kb. All the variants identified by at least one tool were considered for further analysis.

Variant segregation and classification

Sanger sequencing was used in all cases to confirm the findings, and for family segregation, 8 of the 11 *de novo* variants were tested in both parents. Especially for novel variants, downstream targeted inheritance testing was critical to variant classification.

The candidate variants were strictly classified following the ACMG/AMP standards and guidelines for the interpretation of sequence variants [10–12]. We used the VarSome search engine [13] to annotate the identified variants. Discrepancies in the classification in some variants when compared to VarSome are due to the finding of another pathogenic variant in trans in the same gene (PM3), a *de novo* variant (PS2, PM6), a good phenotypic fit (PP4) or functional validation (PS3). A case was considered solved if variants were classified as pathogenic or likely pathogenic. Cases with a variant of unknown significance (VUS) but compatible segregation studies and specific clinical and MRI findings highly suggestive of a given disease were also considered solved. Incidental findings were reviewed in all patients according to published guidelines [14,15].

Functional validation

Several VUSs were functionally tested by different methods, including transfection assays, lipidomics, cDNA sequencing or minigene splicing assays, targeted metabolomics and mitochondrial respiration assays, patch-clamp assays to measure potassium currents, serylation assays and yeast complementation studies, mRNA and protein quantification with immunofluorescence analysis, and quantitative real-time (qRT–PCR) for CNV validation (**additional Tables S4** and **Table S7**).

Detection of the biallelic intronic expansion AAGGG in RFC1

Forty-three patients with negative results in WES were studied**.** We made the diagnosis of pathogenic biallelic expansion in RFC1 in those cases in whom 1) RP-PCR did not repeatedly amplify any product compared to healthy controls, and 2) RP-PCR showed a characteristic "sawtooth" pattern. PCR primers (F:TCAAGTGATACTCCAGCTACACCGTTGC and

R:GTGGGAGACAGGCCAATCACTTCAG) were extracted from Cortese *et al.* [16]. Polymerase chain reactions (PCRs) were performed in a final volume of 50 µl using Taq polymerase (Sigma). The amplification products were visualized after 2% agarose gel electrophoresis (1 h, 60 V). The primers used in the RP-PCR were extracted from Rafehi *et al* [17]. PCR was performed in a final volume of 50 µl using 20 ng genomic DNA, 0.8 mM of the primers CANVAS_FAM_2F and M13R, and 0.2 mM of the first M13R_CANVAS_RE_R using Phusion FLASH High Fidelity MASTER MIX (Thermo Fisher) polymerase. Products were diluted in formamide and analysed in an ABI3730xl DNA Analyser-sequencer using GeneMapper (Applied Biosystems).

PanelApp panels related to Hereditary Spastic Paraplegia or Ataxia

We examined the genes diagnosed in our real-world cohort, including pathogenic variants detected by both WES and WGS and variants in candidate genes validated by us (validated candidate), but excluding phenotype-matching VUSs, in the following 6 different PanelApp panels (https://panelapp.genomicsengland.co.uk/) related to hereditary spastic paraplegia or ataxia.

- Adult-onset hereditary spastic paraplegia (115 genes)
- Childhood onset hereditary spastic paraplegia (145 genes)
- Hereditary spastic paraplegia (110 genes)
- Hereditary ataxia with onset in adulthood (260 genes)
- Hereditary ataxia (178 genes)
- Hereditary ataxia and cerebellar anomalies – childhood onset (469 genes)

Receiver Operating Characteristic (ROC).

The predictive performance of ClinPrior was evaluated by plotting the area under the receiver operating characteristic (AUROC) curve to assess: i) variant prioritization of known disease genes and candidate disease genes in 66,800 synthetic WES analysed, and ii) the optimal number of HPOs required for gene prioritization in 82 patients from the real-world cohort. When querying the 66,800 synthetic WES, we considered the top 50 positions of the variant ranked lists, and when querying the 82 patients (100 runs each), we considered the top 1,000 positions of the gene ranked list after average prioritization (the top ~5% of genes queried). When we look at the top 50 positions (if we do top 50 for variant prioritization (i)) and the top 1,000 positions (if we do top 1,000 for gene prioritization in HPO benchmarking (ii)), the predicted rank is progressively weighted, i.e. the AUROC decreases progressively as the prior rank increases. Thus, if ClinPrior prioritizes at position 1, the resulting AUROC is also 1, and if the rank is 10, the AUROC is 0.8163 (i) or 0.991 (ii). For a rank of 50 or higher (i) or 1,000 or higher (ii) the AUROC is 0. The pROC package in R was used to calculate AUCs along with their standard errors and 95% confidence intervals [18]. We used plotROC [19] and ggplot2 [20] packages to plot the ROC curves. The rank lists for each scenario are available on zenodo [1].

Additional Results

We enrolled 135 families with undiagnosed HSP and/or CA after targeted screening for the most common genetic causes, as described in the Materials and Methods. Based on the phenotypic traits, we classified patients into several groups: i) pure spastic paraplegia, ii) pure CA, and iii) complex phenotypes presenting ataxia and/or spasticity with other symptoms (**Fig. 3A**). The probands were 85 males and 50 females, with ages ranging from 1 month to 83 years (median 32 years). The age of clinical onset ranged from the first month of life to 72

years (median 7 years); age was less than 20 years in 85 patients (63%) and more than 20 years in 50 patients. The median evolution of disease before WES testing was 9 years (1 month - 53 years), and it was longer than 10 years in 56% of patients. Consanguinity was reported in 16 families (12%).

Illustrative clinical cases

1. **Atypical phenotypes**

***GFAP* (IDSPG4):** In this family with 3 generations (the proband, a 46-year-old woman; her son; her cousin; and her cousin's son) affected by adult-onset, mild spastic paraplegia, cranial and medullar MRI findings were initially described as normal. A heterozygous novel missense variant (p.Gly18Val) in the *GFAP* gene cosegregated in all affected family members, as well as in two asymptomatic relatives (the proband's sister and her mother). Mutations in this gene cause Alexander disease (OMIM #203450) [21], an autosomal-dominant leukodystrophy with described adult presentations [22]. We therefore decided to clinically re-evaluate all family members; all of them showed clear signs of cerebellar dysfunction and spastic paraparesis, and two patients were paucisymptomatic, presenting mild alterations in neurological examinations, namely, scoliosis, nystagmus, diplopia, hyperreflexia and the Babinski sign. MRI reexamination of all patients showed notable spinal cord and medulla atrophy, in contrast to what was observed in the less affected patients, who presented mild signal changes in the trunk and less atrophy. Magnetic resonance spectroscopy showed a metabolite profile suggestive of astrocyte hypertrophy, consistent with neuroaxonal degeneration [23]. We validated this variant using a transfection assay to test the capacity of the GFAP protein encoded by the gene carrying p.Gly18Val variant to

induce protein aggregation in the astrocytoma cell Line U251-MG. We did not observe inclusions, dot-like clumps or aggregates in the p.Gly18Val-mutant construct, but we did observe abnormally large cell sizes with long astrocytic processes, a phenotype that was confirmed by quantitative image analysis. Thus, we proposed considering astrocyte hypertrophy as an additional criterion of pathogenicity in the functional evaluation of unreported variants [24].

NDUFS6 **(IDSPG55)**: This patient was a 66-year-old female with spastic paraparesis, hyperreflexia and dysarthria beginning at 10 years of age, accompanied by seizures with good response to antiepileptic drugs, tremor, cavus feet, keratoconus, cataracts, vocal cord weakness, and mild developmental delay. She learned to talk at 5 years of age. Nerve conduction studies revealed axonal sensorimotor neuropathy, and cranial MRI showed a signal intensity alteration in the posterolateral portion of both lenticulate nuclei that was hyperintense in proton density and T2 images. She had an affected sister. They were born from consanguineous parents. Metabolic studies including lactate were not available. Both patients were homozygous for the splicing variant in *NDUFS6*, c.309+5G>A, classified as pathogenic according to the ACMG criteria [11,25]. This variant was previously reported in two different patients with Leigh syndrome and was validated functionally [26,27]. The clinical picture and MRI findings were compatible with Leigh syndrome. Most previous reports suggested that *NDUFS6* pathogenic variants invariably lead to neonatal or early childhood death; instead, these patients showed a slowly progressive disease, as seen in Leigh syndrome caused by mutations in other genes. To date, mutations in more than 80 genes have been found to be related to Leigh syndrome [28]. Therefore, this family expands the clinical phenotype associated with *NDUFS6*.

***ACER3* (IDSPG75)**: This patient, a 16-year-old male born to consanguineous parents, had an affected male cousin. Both had shown early-onset, slowly progressive pure spastic paraparesis and had normal MRI findings. They harbour one homozygous variant in the *ACER3* gene p.(Gly211Cys), classified as pathogenic. Only six cases of *ACER3*-related progressive leukodystrophy have been reported [29,30], and all of these patients showed severe developmental delay with neurological regression, mainly including truncal hypotonia, spasticity, dystonia, seizures, feeding problems and, except for one patient, acquired late-infantile-onset microcephaly. Regarding the imaging findings, they exhibited abnormal periventricular and deep WM signals, a thin corpus callosum and progressive atrophy. These patients had a noticeably milder disease than previously reported patients, thus expanding the clinical spectrum of patients carrying these variants.

***KIDINS220* (IDSPG18)**: This patient was a 10-year-old female with spastic paraparesis and preserved cognition. Her development was considered normal until 4 years of age. The findings of MRI performed at 10 years of age were normal. She was heterozygous for a functionally validated variant (p.Ser1352Glyfs43Ter) in *KIDINS220*. A complex-spastic paraplegia phenotype known as spastic paraplegia, intellectual disability, nystagmus, and obesity syndrome (SINO) has been related to heterozygous variants in this gene, and reports show invariably delayed psychomotor development, intellectual disability, spasticity, cerebral atrophy with reduced white matter volume, nystagmus and increased height and weight [31,32]. Furthermore, homozygous variants lead to a severe autosomal-recessive cognitive disorder characterized by the onset of arthrogryposis and ventriculomegaly (VENARG) that is not compatible with life [33]. This patient had a noticeably milder disease than previously reported patients, thus expanding the clinical spectrum of patients carrying these variants.

***COL6A3* (IDSPG161):** This patient, a 56-year-old woman presenting with severe and congenital axonal peripheral neuropathy, developed pyramidal signs at 3 years of age. She had no previous remarkable family or personal history. We identified a homozygous variant (p.Lys2483Glu) in *COL6A3*, a gene that has been associated with Bethlem myopathy, dystonia and Ulrich congenital muscular dystrophy. More than 10 patients with this mutation have been reported [34,35]. This patient shows an atypical form because predominantly distal involvement and the pattern of neurogenic involvement in the neurophysiological study are present. Patients with both axonal neuropathy and contractures may have a collagen VI-related disorder.

***PMM2* (IDSPG123)**: This patient, a 13-year-old male patient presenting with nonprogressive congenital ataxia with oculomotor apraxia associated with intellectual disability (IQ: 50), neurodevelopmental delay, and hypotonia, was born to healthy nonconsanguineous parents. MRI showed nonprogressive global cerebellar atrophy. Two variants in compound heterozygosity were found in *PMM2*, a gene associated with congenital glycosylation disorder type Ia, CDG Ia (OMIM: # 212065) [36]. The p.Arg141His variant has been reported in numerous publications as the variant most frequently found in patients with CDG Ia [37]. The intronic branch site c.640-23A>G was previously described in a patient with CDG Ia who presented this variant in compound heterozygosity with the p.Arg141His variant. The adenosine substituted in the c.640-23A> G variant is found in the sequence of the branch-site TTCAT, a highly conserved domain within intron 7 that facilitates the removal of the intron in the splicing process [38]. Strikingly, patient IDSPG123 presented a fundamental neurological phenotype and exhibited normal phosphomannomutase activity, similar to that observed in patients with other "mild" splicing variants, who can show normal values of enzymatic activity even in the presence of disease.

2. Novel phenotypes

SPTAN1 **(IDSPG134):** This patient, a male with no relevant personal or familial antecedents, presented with pure spastic paraparesis at 7 years old. His condition was slowly progressive, and at 21 years old, he had normal MRI and nerve conduction study findings. WES detected a nonsynonymous single nucleotide variant (c.55C>T:p.Arg19Trp) in *SPTAN1*, and Sanger segregation confirmed a *de novo* state. *De novo* variants in nonerythrocytic alpha-II-spectrin (*SPTAN1*) cause several phenotypes, including i) early-infantile epileptic encephalopathy type 5 (OMIM #613477), an early onset epilepsy with global developmental delay and spastic quadriplegia [39]; ii) CA with hypoplastic brain structures and intellectual disability without seizures [40]; iii) late-onset hereditary motor neuropathy [41]; and iv) a neurodevelopmental phenotype with peripheral sensorimotor neuropathy [42]. A previous report suggested that biallelic *SPTAN1* variants may lead to pure autosomal-recessive hereditary spastic paraparesis [43]; therefore, we performed WGS in this patient to find a second variant. WGS did not find another deleterious variant. Thus, the case of this patient further expands the phenotypic spectrum associated with *SPTAN1* variants to pure spastic paraparesis with *de novo* heterozygous variants.

LONP1 **(IDSPG166)**: In this family, two siblings, aged 39 and 36 years, had child-onset CA with pyramidal involvement that was slowly progressive. MRI revealed cerebellar atrophy and a dilated 4th ventricle. They had two variants in a compound heterozygous state in the *LONP1* gene, classified as pathogenic and likely pathogenic after applying ACMG criteria. A multisystemic phenotype known as CODAS, an acronym for cerebral ocular dental auricular skeletal syndrome, has been linked to this gene. Affected patients have developmental delay, craniofacial anomalies, cataracts, ptosis, a median nasal groove, delayed tooth eruption,

hearing loss, short stature, delayed epiphyseal ossification, metaphyseal hip dysplasia, and vertebral coronal clefts [44]. In this family, there are predominant neurological manifestations such as ataxia and spastic paraparesis, but these manifestations are not associated with ocular, auricular, or skeletal abnormalities. Thus, this family expands the clinical spectrum associated with *LONP1*.

***PDK3* (IDSPG172):** This patient, a 50-year-old woman presenting with severe and congenital axonal sensorimotor peripheral neuropathy, developed pyramidal signs at 13 years of age. She had no previous remarkable family or personal history. We identified a *de novo* hemizygous variant (c.473G>A) in *PDK3*, a gene that has been associated with Charcot-Marie-Tooth disease, X-linked dominant, 6. Only two patients with this mutation have been reported. The presence of spastic paraplegia had not been previously reported in Charcot-Marie-Tooth disease, X-linked dominant, 6.

3. New inheritance mode

***KCNA1* (IDLNF52)**: This patient, an 8-year-old male presenting with a severe combination of dyskinesia and neonatal epileptic encephalopathy, was born to consanguineous parents. WES revealed a homozygous variant (p.Val368Leu) in *KCNA1*, involving a conserved residue in the pore domain, close to the selectivity signature sequence for K+ ions (TVGYG). Functional analysis showed that the mutant protein alone failed to produce functional channels in the homozygous state, while the coexpression of the mutant protein with wild-type protein had no effects on K+ currents, which were similar to those with wild-type protein alone. Over 50 families affected by the episodic ataxia type 1 disease spectrum have been described with mutations in *KCNA1,* encoding the voltage-gated K+ channel subunit

Kv1.1. All these mutations are either transmitted in an autosomal-dominant mode or found as *de novo* events. This clinical phenotype was reported in a previous publication [45].

SARS1 **(IDSPG64)**: This patient, a 14-year-old female presenting with a global developmental delay, with late acquisition of independent walking at early childhood, motor clumsiness and delayed speech, was able to produce only a few bisyllables during early childhood. Early clumsiness evolved to overt signs of spastic paraparesis that worsened slowly during middle childhood but subsequently remained stable. WES revealed a heterozygous variant (NM_006513.4:c.969+1_969+3del) that causes the ablation of a canonical splice site in the boundary of exon 7 and intron 7. Functional analysis showed that this change causes the inclusion of 16 intronic bp into the cDNA, which results in a loss-of-function, dominant negative effect in complementation assays in S. cerevisiae and serylation assays. This family presents a novel complex spastic paraplegia phenotype and mode of inheritance (*de novo* dominant) for a variant in SARS1. This clinical phenotype was reported in a previous publication [46].

4. Cases with dual diagnoses

POLR3A **and** ***CACNA1A*** **(IDLNF56):** This patient, a 15-year-old female, presented with moderate intellectual disability, ADHD, behavioural abnormalities (obsessions, mood disorder, emotional lability, visual hallucinations), and absence and myoclonic seizures beginning at thirteen years old. She also had nystagmus, strabismus and instability starting in the first years of life. MRI showed periventricular heterogeneous T2 WM hyperintensities and hypointensity in the globus pallidus, thalamic anterolateral nuclei, dentate nuclei, optic radiations, and pyramidal tracts, with mild atrophy of the cerebellar superior vermis. WES

analysis identified two variants in *POLR3A* in compound heterozygosis, classified as pathogenic (c.2171G>A:p.Cys724Tyr) and likely pathogenic (c.2113C>G:p.Pro705Ala), as well as a heterozygous LoF variant in *CACNA1A* (c.1637dup:p.Tyr546Ter) that was revealed to be *de novo* after the segregation study. The patient's clinical picture could be more related to the variant in *CACNA1A*, but the radiological pattern was more consistent with the *POLR3A* variant.

WGS cases with SNVs

We obtained a positive WGS diagnosis for 3 additional families with SNVs in the *SPG7*, *SPTBN2* and *SPTAN1* genes. In one case (IDSPG21), we found a deep intronic splicing variant in the second allele (c.286+853A>G in SPG7) [47]. In a second case (IDSPG125), a single pathogenic variant in one allele was detected in SPTBN2, a gene in which both autosomal-dominant and autosomal-recessive modes of inheritance with the same phenotype of CA had been described (spinocerebellar ataxia, autosomal recessive 14, SCAR14, OMIM #615386 and spinocerebellar ataxia 5, SCA5, OMIM #600224). The absence of findings in WGS ascertained a dominant inheritance mode in this family. Finally, in a third patient (IDSPG134) with pure spastic paraplegia harbouring a single *de novo* variant in SPTAN1 (of described dominant inheritance for a developmental phenotype or recessive for complex spastic paraplegia), WGS ruled out a second variant in trans, thus uncovering a novel phenotype linked to a dominant inheritance mode.

Benchmarking for HPO number optimisation on a real-world cohort.

We performed a benchmark using the 82 causal genes from the real-world cohort patients and their associated HPOs to determine the number of HPOs required for optimal prioritization of

the genes causing the patient's phenotype. For each patient (x), we ran ClinPrior with an increasing number of HPOs (2, 3, 4, until we reached the maximum number of HPOs available from each patient, number of HPOs = z). For each z (2 or 3 or 4 HPOs…), we ran ClinPrior 100 times for each patient (x) with HPOs randomly selected from the patient's total HPOs. The predictive performance of ClinPrior was evaluated by plotting the AUROC to assess the optimal number of HPOs. **Fig. S1** shows that the AUROCs improve as number of HPOs (z) increases.

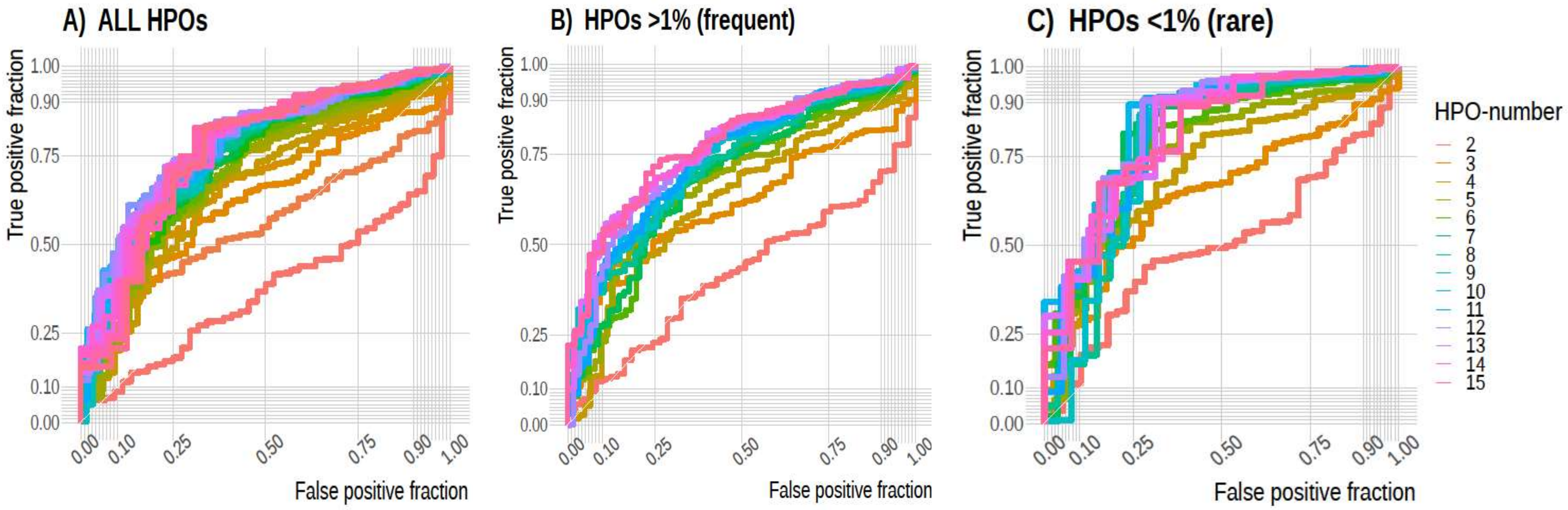

Fig. S1: Gene rank yield by HPOs number. Gene prioritization performance by area under the receiver operating characteristic curve (AUROC) in identifying the optimal number of HPOs. Each ROC curve represents the prioritization performance using a different set of HPOs from each patient, ranging from 2 to 30 randomly selected HPOs. The curves were calculated by integrating 100 runs per patient (82 patients) and considering only the top 1,000 positions (the top ~5% of genes interrogated) with progressive weighting in the prioritization rank. A) All HPOs. B) high-frequency (>1%) HPOs. C) low-frequency (<1%) HPOs.

We assessed whether the optimal number of HPOs varied when the HPOs were more or less specific. Therefore, we divided the HPOs of each patient into two groups according to whether the normalised frequency of each HPO term within the 439,379 gene-HPO associations was less than or greater than 1%. We re-ran ClinPrior 100 times for each patient (x) and increasing number of HPOs (z), but now selecting either common (>1%) or rare (<1%) HPOs. We evaluated the performance by looking at the ROC and once again, we observed that as the number of HPOs increased, the AUROCs improved. Of note, when the HPOs are more specific (less frequent), results improve (AUROC = 0.844 with 10 HPOs). Further, in a situation with both specific and unspecific HPOs, we observe that with a number of HPOs around 20, the yield is close to the best results of scenario with specific HPOs (AUROC=0.790 with 24 HPOs).

Similar performance was obtained when evaluating the frequency of gene prioritization ranks (<1%, <5%, <10%, <20% and >20% of 23,511 total genes) in the same three scenarios: i) no HPO frequency constraint (mix of specific and non-specific terms), ii) HPO frequency above 1% and iii) HPO frequency below 1% (**Fig. S2**). Optimal prioritization performance was achieved with about 7 to 10 HPOs in a rare scenario. In conclusion, we recommend using as many HPOs as possible, but using specific (below 1%) HPOs is also more informative. Interestingly, using common HPOs (more frequent than 1%) does not compromise accurate prioritization, and using a mix of at least 15 specific and non-specific HPOs, ClinPrior can still perform accurate prioritization with similar diagnostic yield as using 7-10 specific HPOs.

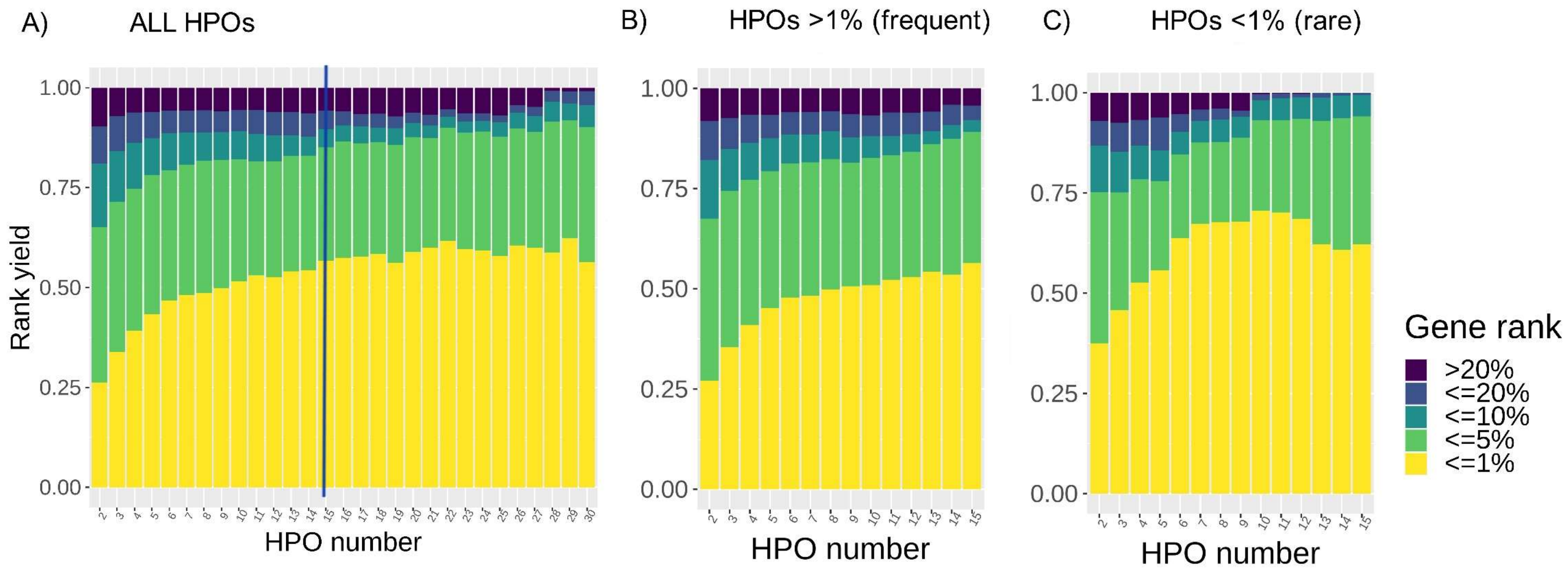

Fig. S2: **ClinPrior performance by HPO number**. Prioritization yield in ranking 82 causal genes to top positions: <1%, <5%, <10%, <20% and >20% (out of 23,511 total genes). The three stacked bar plots show the frequency of gene ranks as the number of HPOs increases. In plot (A) there is no constraint on HPO frequency, and in plots (B) and (C) there is a constraint with HPO frequencies >1% and <1%, respectively. The difference in the maximum number of HPOs displayed is because in a patient with an average of 30 HPOs (A), approximately 15 will have a frequency >1% (B) and the remaining 15 will have a frequency <1% (C).

Additional References

1. Schlüter A. ClinPrior: An algorithm for diagnosis and novel gene discovery by network-based prioritization [Internet]. Zenodo. 2023. Available from: https://zenodo.org/record/7945507
2. Zook JM, Chapman B, Wang J, Mittelman D, Hofmann O, Hide W, et al. Integrating human sequence data sets provides a resource of benchmark SNP and indel genotype calls. Nature Biotechnology 2014 32:3 [Internet]. 2014 [cited 2022 May 11];32:246–51. Available from: https://www.nature.com/articles/nbt.2835
3. Schlüter A. Hereditary spastic paraplegias expanded network [Internet]. NDEx. 2023. Available from: https://www.ndexbio.org/index.html#/network/9a5c7fd0-e61f-11eb-b666-0ac135e8bacf?accesskey=d786cfb7addf9e47df34e3c149d6eb7e3c728a97bcfa8f4676a8dda072365e1c
4. Schluter A, Espinosa L, Fourcade S, Galino J, Lopez E, Ilieva E, et al. Functional genomic analysis unravels a metabolic-inflammatory interplay in adrenoleukodystrophy. Hum Mol Genet. 2012;21:1062–77.
5. Falcon S, Gentleman R. Using GOstats to test gene lists for GO term association. Bioinformatics. 2007;23:257–8.
6. Auwera GAV der., O'Connor BD. Genomics in the Cloud : Using Docker, GATK, and WDL in Terra. :502.
7. Minoche AE, Lundie B, Peters GB, Ohnesorg T, Pinese M, Thomas DM, et al. ClinSV: clinical grade structural and copy number variant detection from whole genome sequencing data. Genome Med. 2021;13.
8. Boeva V, Zinovyev A, Bleakley K, Vert JP, Janoueix-Lerosey I, Delattre O, et al. Control-free calling of copy number alterations in deep-sequencing data using GC-content normalization. Bioinformatics. 2011;27.
9. Boeva V, Popova T, Bleakley K, Chiche P, Cappo J, Schleiermacher G, et al. Control-FREEC: A tool for assessing copy number and allelic content using next-generation sequencing data. Bioinformatics. 2012;28.
10. Brandt T, Sack LM, Arjona D, Tan D, Mei H, Cui H, et al. Adapting ACMG/AMP sequence variant classification guidelines for single-gene copy number variants. Genetics in Medicine. 2020;22:336–44.
11. Amendola LM, Jarvik GP, Leo MC, McLaughlin HM, Akkari Y, Amaral MD, et al. Performance of ACMG-AMP Variant-Interpretation Guidelines among Nine Laboratories in the Clinical Sequencing Exploratory Research Consortium. Am J Hum Genet. 2016;98:1067–76.
12. Richards S, Aziz N, Bale S, Bick D, Das S, Gastier-Foster J, et al. Standards and guidelines for the interpretation of sequence variants: a joint consensus recommendation of the American College of Medical Genetics and Genomics and the Association for Molecular Pathology. Genetics in Medicine. 2015;17:405–24.
13. Kopanos C, Tsiolkas V, Kouris A, Chapple CE, Albarca Aguilera M, Meyer R, et al. VarSome: the human genomic variant search engine. Bioinformatics. 2019;35:1978–80.
14. Kalia SS, Adelman K, Bale SJ, Chung WK, Eng C, Evans JP, et al. Recommendations for reporting of secondary findings in clinical exome and genome sequencing, 2016 update (ACMG SF v2.0): A policy statement of the American College of Medical Genetics and Genomics. Genetics in Medicine. 2017;19:249–55.
15. Miller DT, Lee K, Chung WK, Gordon AS, Herman GE, Klein TE, et al. ACMG SF v3.0 list for reporting of secondary findings in clinical exome and genome sequencing: a policy statement of the American College of Medical Genetics and Genomics (ACMG). Genetics in Medicine. 2021;

16. Cortese A, Simone R, Sullivan R, Vandrovcova J, Tariq H, Yau WY, et al. Biallelic expansion of an intronic repeat in RFC1 is a common cause of late-onset ataxia. Nat Genet. 2019;51.
17. Rafehi H, Szmulewicz DJ, Bennett MF, Sobreira NLM, Pope K, Smith KR, et al. Bioinformatics-Based Identification of Expanded Repeats: A Non-reference Intronic Pentamer Expansion in RFC1 Causes CANVAS. The American Journal of Human Genetics. 2019;105.
18. Robin X, Turck N, Hainard A, Tiberti N, Lisacek F, Sanchez JC, et al. pROC: an open-source package for R and S+ to analyze and compare ROC curves. BMC Bioinformatics [Internet]. 2011;12:77. Available from: http://www.ncbi.nlm.nih.gov/entrez/query.fcgi?cmd=Retrieve&db=PubMed&dopt=Citation&list_uids=21414208
19. Sachs MC. Plotroc: A tool for plotting ROC curves. J Stat Softw. 2017;79.
20. Wickham H. ggplot2 - Elegant Graphics for Data Analysis | Hadley Wickham | Springer. Springer Science & Business Media. 2017.
21. Brenner M, Johnson AB, Boespflug-Tanguy O, Rodriguez D, Goldman JE, Messing A. Mutations in GFAP, encoding glial fibrillary acidic protein, are associated with Alexander disease. Nat Genet. 2001;27:117–20.
22. Pareyson D, Fancellu R, Mariotti C, Romano S, Salmaggi A, Carella F, et al. Adult-onset Alexander disease: a series of eleven unrelated cases with review of the literature. Brain. 2008;131:2321–31.
23. Brockmann K, Dechent P, Meins M, Haupt M, Sperner J, Stephani U, et al. Cerebral proton magnetic resonance spectroscopy in infantile Alexander disease. J Neurol. 2003;250.
24. Casasnovas C, Verdura E, Vélez V, Schlüter A, Pons-Escoda A, Homedes C, et al. A novel mutation in the *GFAP* gene expands the phenotype of Alexander disease. J Med Genet. 2019;jmedgenet-2018-105959.
25. Richards S, Aziz N, Bale S, Bick D, Das S, Gastier-Foster J, et al. Standards and guidelines for the interpretation of sequence variants: A joint consensus recommendation of the American College of Medical Genetics and Genomics and the Association for Molecular Pathology. Genetics in Medicine. 2015;17:405–24.
26. Ogawa E, Shimura M, Fushimi T, Tajika M, Ichimoto K, Matsunaga A, et al. Clinical validity of biochemical and molecular analysis in diagnosing Leigh syndrome: a study of 106 Japanese patients. J Inherit Metab Dis. 2017;40.
27. Rouzier C, Chaussenot A, Fragaki K, Serre V, Ait-El-Mkadem S, Richelme C, et al. NDUFS6 related Leigh syndrome: a case report and review of the literature. J Hum Genet. 2019;64.
28. Rahman J, Noronha A, Thiele I, Rahman S. Leigh map: A novel computational diagnostic resource for mitochondrial disease. Ann Neurol. 2017;81.
29. Dehnavi AZ, Heidari E, Rasulinezhad M, Heidari M, Ashrafi MR, Hosseini MM, et al. ACER3-related leukoencephalopathy: expanding the clinical and imaging findings spectrum due to novel variants. Hum Genomics. 2021;15.
30. Edvardson S, Yi JK, Jalas C, Xu R, Webb BD, Snider J, et al. Deficiency of the alkaline ceramidase ACER3 manifests in early childhood by progressive leukodystrophy. J Med Genet. 2016;53:389–96.
31. Josifova DJ, Monroe GR, Tessadori F, de Graaff E, van der Zwaag B, Mehta SG, et al. Heterozygous *KIDINS220/ARMS* nonsense variants cause spastic paraplegia, intellectual disability, nystagmus, and obesity. Hum Mol Genet. 2016;25.
32. Schmieg N, Thomas C, Yabe A, Lynch DS, Iglesias T, Chakravarty P, et al. Novel Kidins220/ARMS Splice Isoforms: Potential Specific Regulators of Neuronal and Cardiovascular Development. PLoS One. 2015;10.

33. Mero I-L, Mørk HH, Sheng Y, Blomhoff A, Opheim GL, Erichsen A, et al. Homozygous KIDINS220 loss-of-function variants in fetuses with cerebral ventriculomegaly and limb contractures. Hum Mol Genet. 2017;26.
34. Stavusis J, Micule I, Wright NT, Straub V, Topf A, Panadés-de Oliveira L, et al. Collagen VI-related limb-girdle syndrome caused by frequent mutation in COL6A3 gene with conflicting reports of pathogenicity. Neuromuscul Disord [Internet]. 2020 [cited 2023 Jan 26];30:483–91. Available from: https://pubmed.ncbi.nlm.nih.gov/32448721/
35. Briñas L, Richard P, Quijano-Roy S, Gartioux C, Ledeuil C, Lacène E, et al. Early onset collagen VI myopathies: Genetic and clinical correlations. Ann Neurol [Internet]. 2010 [cited 2023 Jan 26];68:511–20. Available from: https://onlinelibrary.wiley.com/doi/full/10.1002/ana.22087
36. Jaeken J, Eggermont E, Stibler H. AN APPARENT HOMOZYGOUS X-LINKED DISORDER WITH CARBOHYDRATE-DEFICIENT SERUM GLYCOPROTEINS. The Lancet. 1987;330.
37. Kjaergaard S. Congenital disorder of glycosylation type Ia (CDG-Ia): phenotypic spectrum of the R141H/F119L genotype. Arch Dis Child. 2001;85.
38. Vuillaumier-Barrot S, Le Bizec C, De Lonlay P, Madinier-Chappat N, Barnier A, Dupré T, et al. PMM2 intronic branch-site mutations in CDG-Ia. Mol Genet Metab. 2006;87.
39. Saitsu H, Tohyama J, Kumada T, Egawa K, Hamada K, Okada I, et al. Dominant-Negative Mutations in α-II Spectrin Cause West Syndrome with Severe Cerebral Hypomyelination, Spastic Quadriplegia, and Developmental Delay. The American Journal of Human Genetics. 2010;86.
40. Gartner V, Markello TC, Macnamara E, De Biase A, Thurm A, Joseph L, et al. Novel variants in *SPTAN1* without epilepsy: An expansion of the phenotype. Am J Med Genet A. 2018;
41. Beijer D, Deconinck T, De Bleecker JL, Dotti MT, Malandrini A, Urtizberea JA, et al. Nonsense mutations in alpha-II spectrin in three families with juvenile onset hereditary motor neuropathy. Brain. 2019;142.
42. Ylikallio E, Ritari N, Sainio M, Toppila J, Kivirikko S, Tyynismaa H, et al. *De novo SPTAN1* mutation in axonal sensorimotor neuropathy and developmental disorder. Brain. 2020;143.
43. Leveille E, Estiar MA, Krohn L, Spiegelman D, Dionne-Laporte A, Dupré N, et al. SPTAN1 variants as a potential cause for autosomal recessive hereditary spastic paraplegia. J Hum Genet. 2019;64.
44. Strauss KA, Jinks RN, Puffenberger EG, Venkatesh S, Singh K, Cheng I, et al. CODAS Syndrome Is Associated with Mutations of LONP1, Encoding Mitochondrial AAA+ Lon Protease. The American Journal of Human Genetics. 2015;96.
45. Verdura E, Fons C, Schlüter A, Ruiz M, Fourcade S, Casasnovas C, et al. Complete loss of KCNA1 activity causes neonatal epileptic encephalopathy and dyskinesia. J Med Genet. 2020;57.
46. Verdura E, Senger B, Raspall-Chaure M, Schlüter A, Launay N, Ruiz M, et al. Loss of seryl-tRNA synthetase (SARS1) causes complex spastic paraplegia and cellular senescence. J Med Genet [Internet]. 2022 [cited 2022 Aug 31];jmedgenet-2022-108529. Available from: https://pubmed.ncbi.nlm.nih.gov/36041817/
47. Verdura E, Schlüter A, Fernández-Eulate G, Ramos-Martín R, Zulaica M, Planas-Serra L, et al. A deep intronic splice variant advises reexamination of presumably dominant *SPG7* Cases. Ann Clin Transl Neurol. 2020;7.
48. Oladnabi M, Musante L, Larti F, Hu H, Abedini SS, Wienker T, et al. New evidence for the role of calpain 10 in autosomal recessive intellectual disability: Identification of two novel nonsense variants by exome sequencing in Iranian families. Arch Iran Med. 2015;18.

49. Djordjevic D, Pinard M, Gauthier MS, Smith-Hicks C, Hoffman TL, Wolf NI, et al. De novo variants in POLR3B cause ataxia, spasticity, and demyelinating neuropathy. Am J Hum Genet; 2021;108:186–93.
50. Guasto A, Dubail J, Aguilera-Albesa S, Paganini C, Vanhulle C, Haouari W, et al. Biallelic variants in SLC35B2 cause a novel chondrodysplasia with hypomyelinating leukodystrophy . Brain. 2022;145.
51. Soehn AS, Rattay TW, Beck-Wödl S, Schäferhoff K, Monk D, Döbler-Neumann M, et al. Uniparental disomy of chromosome 16 unmasks recessive mutations of FA2H /SPG35 in 4 families. Neurology. 2016;87.
52. Rattay TW, Lindig T, Baets J, Smets K, Deconinck T, Söhn AS, et al. FAHN/SPG35: a narrow phenotypic spectrum across disease classifications. Brain. 2019;142:1561–72.
53. Vélez-Santamaría V, Verdura E, Macmurdo C, Planas-Serra L, Schlüter A, Casas J, et al. Expanding the clinical and genetic spectrum of PCYT2-related disorders. Brain. 2020;143.
54. Verdura E, Rodríguez-Palmero A, Vélez-Santamaria V, Planas-Serra L, de la Calle I, Raspall-Chaure M, et al. Biallelic PI4KA variants cause a novel neurodevelopmental syndrome with hypomyelinating leukodystrophy. Brain. 2021;
55. García-Cazorla À, Verdura E, Juliá-Palacios N, Anderson EN, Goicoechea L, Planas-Serra L, et al. Impairment of the mitochondrial one-carbon metabolism enzyme SHMT2 causes a novel brain and heart developmental syndrome. Acta Neuropathol. 2020;140.

Table S1

Reference	Article	Total families	Families with definitive positive result	Diagnostic Yield	Clinically relevant variant (adding VUS)	% Detection of clinically relevant variants	Population characteristics	PMID
da Graça, 2021	Diagnostic Yield of Whole Exome Sequencing for Adults with Ataxia: a Brazilian Perspective	**76**	25	**33%**	VUS no reported		Non-Caucasian ataxic adult population. In Cerebellar ataxia with Pyramidal signs subgroup, WES showed a better performance.	33956305
Kim, 2020	Clarification of undiagnosed ataxia using whole-exome sequencing with clinical implications	**68**	18	**26%**	32	47%	WES in korean patients with either a positive family history of ataxia or an onset age <50 years. SPG7 and SPG11 were the most common diagnoses.	32961395
Hengel, 2020	First-line exome sequencing in Palestinian and Israeli Arabs with neurological disorders is efficient and facilitates disease gene discovery	**83**	35	**42%**	42	51%	WES in highly consanguineous cohort with neurological disorders (at least 2 affected members)	32214227
Ngo, 2020	A diagnostic ceiling for exome sequencing in cerebellar ataxia and related neurological disorders	**260**	65	**25%**	135	52%	Mostly sporadic adult onset spastic paraplegia or cerebellar ataxia. WES + CNV / STR analysis	31692161
Kim, 2019	Increased Diagnostic Yield of Spastic Paraplegia with or Without Cerebellar Ataxia Through Whole-Genome Sequencing	**18**	7	**39%**	VUS no reported		WGS in Spastic Paraplegia with or whithout cerebellar ataxia	31104286
Mu, 2019	The utility of whole exome sequencing in diagnosing neurological disorders in adults from a highly consanguineous population	**24**	13	**54%**	VUS no reported		Adults with undiagnosed neurologic disorders (mostly ataxia and developmental delay) with consanguinity. AR disorders comprised 77% of diagnoses, due to homozygous variants.	30724636
De Souza, 2017	New genetic causes for complex hereditary spastic paraplegia	**17**	12	**71%**	VUS no reported		Complex HSP. 10 known variants and 2 new variants (in silico) in genes not related to HSP previously.	28716262
Nibbeling, 2017	Exome sequencing and network analysis identifies shared mechanisms underlying spinocerebellar ataxia	**20**	9	**45%**	VUS no reported		WES in hereditary ataxias, validation of 5 new genes.	29053796

**PRE- ACMG**								
Kuperberg, 2016	Utility of Whole Exome Sequencing for Genetic Diagnosis of Previously Undiagnosed Pediatric Neurology Patients	57	28	**49%**			Utility of WES in neurogenetic disorders. (Symptoms: GDD, ataxia:57%, muscular, seizures, dystonia) Change of treatment in 5 patients.	27572814
Van de Warrenburg, 2016	Clinical exome sequencing for cerebellar ataxia and spastic paraplegia uncovers novel gene–disease associations and unanticipated rare disorders	76	26	**34%**			Clinical exome (200 genes) in familial HSP and CA (AO<45 or >1 affected) Reveals 'old' gene–'new' disease associations (SPG7-CA, TH-HSP) and uncovers unanticipated disorders.	27165006
Kara, 2015	Genetic and phenotypic characterization of complex hereditary spastic paraplegia	66	20	**30%**			Clinical exome 26%: 97 cHSP. 1) SPG11 (MLPA)-> 30 (30%). 2) clinical exome in 20/66: 26 (4813 genes) + 7 WES. Characterizes a extensive SPG11 cohort.	27217339
Pyle, 2015	Exome sequencing in undiagnosed inherited and sporadic ataxias	22	9	**41%**			DY WES in Ataxia: 64% (9/22 european fam, CO and AO) PreACMG: 41% path, 23% probably path). De novo mutations, validated disease genes previously described in isolated families, and broadened the clinical phenotype of known disease genes.	25497598
Farwell, 2014	Enhanced utility of family-centered diagnostic exome sequencing with inheritance model-based analysis: results from 500 unselected families with undiagnosed genetic conditions.	500	152	**30%**			DY WES in neurogenetic disorders: 30% PreACMG. Ataxias DY:44%. Diagnostic rate was significantly higher among trio (37%) compared with singleton (21%)	25356970
Novarino, 2014	Exome Sequencing Links Corticospinal Motor Neuron Disease to Common Neurodegenerative Disorders	55	18	**33%**			DY WES in SPG: 55 fam AR: 18 previously unknown HSP genes, identified and validated (functionally in zebra fish or genetically).	24482476
Fogel, 2014	Exome sequencing in the clinical diagnosis of sporadic or familial cerebellar ataxia	76	16	**21%**			DY in cerebellar ataxia (predominantly adult sporadic onset)	25133958
Ohba, 2013	Diagnostic utility of whole exome sequencing in patients showing cerebellar and/or vermis atrophy in childhood	23	9	**39%**			DY WES in children with Cerebellar Atrophy.	24091540

Table S1. NGS diagnostic yield among published ataxia/HSP studies.

Table S2

Gene	ID	Sex	Age	Age at onset	Affected relatives	Consanguinity	Inheritance mode	Pyramidal involvement	Cerebellar involvement	Neuropathy	Cognitive / Behaviour	Extrapyramidal features	Seizures	MRI findings	Other findings (exam or investigation tests)	Diagnostic technique	Treatment
ACER3	IDSPG75	Female	16	1	YES (brother)	YES	AR (Homozygous)	Pure spastic paraplegia	None	None	None	None	None	Normal	None	WES	
ALS2	IDSPG74	Female	11	3	None	YES	AR (Homozygous)	Spastic paraparesis	None	None	GDD + learning difficulty	None	None	Normal	None	WES	
AMPD2	IDSPG78	Male	34	0	None	None	AR (Homozygous)	Spastic paraparesis	None	Axonal sensory neuropathy	GDD + ID	None	None	Normal	None	WES	
AP4B1	IDSPG167	Female	47	20	None	None	AR (Compouund Heterozygous)	Spastic paraparesis	Ataxia	None	None	Head tremor	Abdominal myoclonus	Normal	None	WES	
ATL1	IDSPG5	Male	40	10	YES (three affected generations)	None	AD	Spastic paraparesis	None	Distal hereditary motor neuropathy	None	None	None	Normal	None	WES	
ATL1	IDSPG70	Female	8	2	YES (three affected generations)	None	AD	Spastic paraparesis	None	None	None	None	None	Normal	Seborrheic dermatitis	WES	
ATL1	IDSPG168	Male	10	1	None	None	AD	Spastic paraparesis	None	None	Learning disability	None	None	Normal	None	WES	
BCKDK	IDSPG47.0	Male	45	12	YES (sister)	YES	AR (Homozygous)	Spastic paraparesis	None	None	GDD + ID	None	Seizures	Frontal T2 WM HI, caudate nuclei HI, enlarged IV ventricle	None	WES (Reanalysis)	BCAA supplementation and high-protein diet
BSCL2	IDSPG29	Male	48	2	YES (mother)	None	AD	Spastic paraparesis	None	Demyelinating sensorimotor peripheral neuropathy	None	None	None	Normal	None	WES	
BSCL2	IDSPG112	Male	34	12	YES (three affected generations)	None	AD	Pyramidal signs	None	Axonal sensorimotor peripheral neuropathy	None	None	None	Normal	None	WES	
BSCL2	IDSPG145	Male	71	57	YES (two affected generations)	None	AD	None	None	Distal hereditary motor neuropathy	None	None	None	Cerebellar atrophy	None	WES	
BSCL2	IDSPG157	Female	63	57	None	None	AD	Pure spastic paraplegia	None	None	None	None	None	Normal	None	WES	
CACNA1A	IDSPG126	Male	17	1	YES (sister)	None	AD	None	Episodic Ataxia	None	None	None	Seizures	Vermian atrophy	EEG: Centroparietal epileptiform discharges	WES (Reanalysis)	
CACNA1A / POLR3A	IDLNF56	Female	16	1	None	None	AD/AR (Compouund Heterozygous)	Spastic paraparesis	Ataxia	None	GDD + behaviour disorder	None	YES	Periventricular T2 WM HI	Hepatic steatosis, obesity, amenorrhea, hypertrichosis	WES	
CAPN1	IDSPG2	Male	62	40	YES (brother)	None	AR (Homozygous)	Spastic paraparesis	None	None	None	None	None	Periventricular T2 WM HI Cerebral atrophy	None	WES	
CC2D2A	IDLNF129	Male	6	1	None	YES	AR (Compouund Heterozygous)	None	Ataxia	None	GDD + ASD	None	None	Dysplastic cerebellar vermis	High hydroxybutyrate, normalized later. Abnormal VEP	WES	
COL6A3	IDSPG161	Female	56	3	None	None	AR (Homozygous)	Pyramidal signs	None	Distal hereditary motor neuropathy	None	None	None	None	None	WES (Reanalysis)	
CTNNB1	IDSPG139	Female	12	7m	None	None	AD	Spastic tetraparesis	None	None	Learning disability	Dystonia	None	Normal	Strabismus, wheelchair	WES	
CYP2U1	IDLNF40	Male	10	1	YES (brother)	YES	AR (Homozygous)	Spastic paraparesis	None	None	GDD + ID	Dystonia	None	Periventricular WM HI WM, CC and BS atrophy	None	WES	
DDHD2	IDSPG61	Female	6	2	None	YES	AR (Homozygous)	Spastic tetraplegia	None	None	GDD + ID	None	None	Periventricular T2 WM HI, thin CC	None	WES	
DLG4	IDSPG107	Female	16	1	None	None	AD	Spastic paraparesis	None	None	Language disorder	None	Rolandic epilepsy	Left hippocampal malrotation with dysmorphic and dilated temporal horn	None	WES (Reanalysis)	
DSTYK / MMUT	IDLNF84	Male	74	72	None	YES	AR (Homozygous/Homozygous)	Pyramidal signs	None	None	Cognitive decline	None	None	Periventricular T2 WM HI	Increased urine methylmalonic acid Dysphagia Osteopenia	WES	
ERBB4	IDSPG38	Female	54	40	YES (mother)	None	AD	Spastic paraparesis	None	Distal hereditary motor neuropathy	None	None	None	Normal	Chronic neurogen pattern in EMG study	WES (Reanalysis)	
FA2H	IDSPG10	Female	27	4	None	None	AR (Homozygous)	Spastic tetraparesis	Ataxia	None	Mild ID	Dystonia	None	Normal	Wheelchair	WES	
FARS2	IDSPG116	Male	9	1	None	None	AR (Compouund Heterozygous)	Spastic paraparesis	Ataxia	None	None	None	None	Hypomyelination	None	WES	

Gene	ID	Sex	Age	Age at onset	Affected relatives	Consanguinity	Inheritance mode	Pyramidal involvement	Cerebellar involvement	Neuropathy	Cognitive / Behaviour	Extrapiramidal features	Seizures	MRI findings	Other findings (exam or investigation tests)	Diagnostic technique	Treatment
GFAP	IDSPG4	Female	61	20	YES (three affected generations)	None	AD	Spastic paraparesis	Nystagmus	None	None	None	None	Mild signal change in brainstem	Non alcoholic fatty liver disease	WES	
IFIH1	IDSPG3	Male	85	56	YES (brother)	None	AD	Pure spastic paraplegia	None	None	None	None	None	Normal	None	WES (Reanalysis)	
IFIH1	IDSPG60	Female	6	1	None	None	AD	Pure spastic paraplegia	None	None	None	None	None	None	None	WES (Reanalysis)	
IQSEC2	IDSPG127	Male	11	0	None	None	X-Linked	Spastic paraparesis	Ataxia	None	ASD + Behavioral Disorder	None	None	None	None	WES (Reanalysis)	
IRF2BPL	IDSPG44	Male	12	0	None	None	AD	Spastic paraparesis	None	None	GDD + ID	None	None	Normal	Tiptoe gait	WES (Reanalysis)	
IRF2BPL	IDLNF58	Male	16	5	None	None	AD	Spastic tetraplegia	Ataxia	None	GDD + regression	Rigid akynetic syndrome	None	Normal	Vertical gaze palsy, hypomimia	WES (Reanalysis)	
KCNA1	IDLNF52	Female	8	0	None	YES	AR (Homozygous)	None	None	None	GDD + severe ID	Choreo-dystonic movements	Epileptic Encephalopathy	Normal	EEG: Slow and disorganised background with occipital paroxysmal activity	WES (Reanalysis)	Seizure control with a sodium channel blocker (oxcarbazepine)
KIDINS220	IDSPG118	Female	10	4	None	None	AD	Pure spastic paraplegia	None	None	None	None	None	Normal	None	WES	
KIF1A	IDSPG39	Male	11	3	None	None	AD	Pure spastic paraplegia	None	None	None	None	None	Normal	Abnormal VEP	WES	
KIF1A	IDSPG110	Female	8	1	YES (father)	None	AD	Spastic paraparesis	None	Axonal sensorimotor peripheral neuropathy	None	None	None	Normal	None	WES	
KIF1A	IDSPG108	Male	8	1	None	None	AD	Spastic paraparesis	None	None	ASD	None	None	Thin CC	None	WES	
KIF5A	IDSPG17	Male	28	21	None	None	AD	Spastic paraparesis	None	Axonal sensorimotor peripheral neuropathy	None	None	None	Normal	Idiopatic interstitial tubular acidosis	WES	
KMT2B	IDSPG114	Male	19	3	None	None	AD	Spastic paraparesis	None	None	ADHD	Dystonia	None	Normal	None	WES (Reanalysis)	
L2HGDH / CAPN10	IDSPG47.1	Female	41	5	YES (brother)	YES	AR (Homozygous/homozygous)	Spastic paraparesis	None	None	GDD + ID	None	Seizures	Frontal T2 WM HI	None	WES	
LAMA1	IDSPG56	Female	10	1	None	None	AR (Compouund Heterozygous)	Hypotonia	None	Distal hereditary motor neuropathy	GDD + ID	None	Seizures	Subcortical hypomielination, thin CC	Abnormal VEP, nocturnal respiratory insufficiency	WES (Reanalysis)	
LONP1	IDSPG166	Female	40	40	YES (brother)	None	AR (Compouund Heterozygous)	Spastic paraparesis	Ataxia	None	None	None	None	Normal	Prion protein 14.3.3 Positive	WES	
MORC2	IDSPG37	Female	48	11	None	None	AD	Pyramidal signs	None	Axonal sensorimotor peripheral neuropathy	None	None	None	Normal	None	WES	
NDUFS6	IDSPG55	Female	67	10	YES (sister)	None	AR (Homozygous)	Spastic paraparesis	None	Axonal sensorimotor peripheral neuropathy	GDD	Tremor	Seizures	Lenticular nuclei HI	None	WES	
PAX6	IDSPG155	Male	51	0	YES (son)	None	AD	Pyramidal signs	None	Axonal sensorimotor peripheral neuropathy	ID	None	None	Normal	Aniridia	WES	
PCYT2	IDSPG27	Male	60	19	YES (sister)	YES	AR (Homozygous)	Spastic paraparesis	Ataxia	Distal hereditary motor neuropathy	None	None	None	Normal	None	WES (Reanalysis)	
PDK3	IDSPG172	Male	50	13	None	None	X-Linked	Pyramidal signs	None	Axonal sensorimotor peripheral neuropathy	None	None	None	Normal	None	WES	
PI4KA	IDSPG16	Male	43	17	None	None	AR (Compouund Heterozygous)	Spastic paraparesis	None	None	None	None	None	Arachnoid cyst of the posterior fossa and cervical spinal cord atrophy	Chron´s disease	WES (Reanalysis)	
PI4KA	IDSPG149	Male	12	3	None	None	AR (Compouund Heterozygous)	Spastic paraparesis	None	None	Learning disability	None	None	Normal	None	WES (Reanalysis)	
PMM2	IDSPG123	Male	13	0	None	None	AR (Compouund Heterozygous)	None	Ataxia	None	GDD + ID	None	None	Hyperintensities in cerebellar cortex, cerebellar atrophy	Oculomotor apraxia	WES (Reanalysis)	
PNKP / AFG3L2	IDSPG42	Female	26	6	None	None	AR (Compouund Heterozygous) / AD	None	Ataxia	Demyelinating sensorimotor peripheral neuropathy	Learning disability	None	None	Normal	Hand stereotyped behaviour guttural tics, obesity, hirsutism. Abnormal VEP, hypoalbuminemia	WES (Reanalysis)	
PNPLA6	IDSPG13	Male	36	32	None	None	AR (Compouund Heterozygous)	Pure spastic paraplegia	None	None	None	None	None	Normal	None	WES	

Gene	ID	Sex	Age	Age at onset	Affected relatives	Consanguinity	Inheritance mode	Pyramidal involvement	Cerebellar involvement	Neuropathy	Cognitive / Behaviour	Extrapiramidal features	Seizures	MRI findings	Other findings (exam or investigation tests)	Diagnostic technique	Treatment
POLG	IDSPG113	Male	39	30	None	None	AR (Compouund Heterozygous)	Spastic paraparesis	Ataxia	Distal hereditary motor neuropathy	Learning disability	None	None	Periventricular T2 WM HI	None	WES (Reanalysis)	Q10 coenzyme
POLR3A	IDSPG14	Male	51	14	None	None	AR (Compouund Heterozygous)	Spastic paraparesis	Ataxia	None	None	None	None	Periventricular T2 WM HI	None	WES (Re-analysis)	
POLR3A	IDSPG28	Male	76	28	YES (brother)	None	AR (Compouund Heterozygous)	Pure spastic paraplegia	None	None	None	None	None	Cervical spinal cord atrophy	Wheelchair; abnormal SSEP	WES	
POLR3A	IDSPG109	Male	8	1	None	YES	AR (Homozygous)	Spastic paraparesis	Ataxia	None	Learning disability	Bradikynesia, dystonia	Myoclonus	Normal	None	WES (Reanalysis)	
POLR3A	IDSPG122	Male	35	22	None	None	AR (Compouund Heterozygous)	Spastic paraparesis	None	None	None	Head, vocal tremor	Seizures	Normal	None	WES	
POLR3B	IDSPG66	Male	13	7	YES (mother)	None	AD	Pure spastic paraplegia	None	None	None	None	None	None	None	WES (Reanalysis)	
REEP1	IDSPG12	Male	80	22	YES (three affected generations)	None	AD	Spastic paraparesis	Ataxia	None	None	Tremor	None	Normal	None	WES	
REEP1	IDSPG124	Female	14	1	None	None	AD	Spastic paraparesis	None	Distal hereditary motor neuropathy	None	None	None	Normal	Neonatal jaundice	WES	
RFC1	IDSPG173	Female	65	60	None	YES	AR	None	Ataxia	Axonal sensory neuropathy	None	None	None	None	None	RFC1 expansion	
RFC1	IDSPG184	Male	69	60	None	None	AR	None	Pure cerebellar Ataxia	None	None	None	None	None	None	RFC1 expansion	
RFC1	IDSPG192	Female	75	60	None	None	AR	None	Ataxia	Axonal sensory neuropathy	None	None	None	None	Cough, abnormal Head impulse test	RFC1 expansion	
RFC1	IDSPG194	Female	63	55	None	None	AR	None	Ataxia	Axonal sensory neuropathy	None	None	None	None	None	RFC1 expansion	
RFC1	IDSPG198	Male	61	48	None	None	AR	None	Ataxia	Axonal sensory neuropathy	None	None	None	None	None	RFC1 expansion	
RNASEH2B	IDLNF141	Male	9	1	None	None	AR (Homozygous)	Spastic tetraplegia	None	None	GDD	None	None	Multifocal subcortical hyperintensities, improvement (4y)	Abnormal VLCFA, onset after infection	WES	JAK1 and JAK2 inhibitors Monitoring of immuno-mediated manifestations
SARS1	IDSPG64	Female	14	0	None	None	AD	Spastic paraparesis	Ataxia	None	GDD + ID	None	Seizures	Non progressive puntiform frontal subcortical HI	Strabismus, abnormal VEP	WES (Reanalysis)	
SEPSECS	IDLNF67	Male	11	1	YES (brother)	YES	AR (Homozygous)	Spastic tetraplegia	Ataxia	None	GDD	None	None	Cerebellar and bilateral thalamic atrophy	Abnormal VEP	WES	
SEPSECS	IDLNF121	Male	5	1	None	None	AR (Homozygous)	Spastic paraparesis	Nystagmus	None	GDD + ID	Tremor	None	Periventricular T2 WM HI Cerebellar atrophy	None	WES	
SHMT2	IDSPG26	Male	24	1	YES (brother)	None	AR (Homozygous)	Spastic paraparesis	Ataxia	None	GDD + ID	None	None	Hypoplastic CC, perisylvian polymicrogyria-like pattern	Dysmorphic features, congenital mycrocephaly, hypertrophic cardiomyopathy	WES (Reanalysis)	
SLC25A46	IDSPG93	Male	49	25	None	None	AR (Homozygous)	Pyramidal signs	Ataxia	Axonal sensorimotor peripheral neuropathy	None	None	None	Occipitoparietal billateral atrophy,superior cerebellar atrophy, mega cisterna magna	Billateral optic atrophy, hypoacusia, mild dysmorphic features	WES	
SLC35B2	IDLNF68	Female	8	0	None	None	AR (Homozygous)	Spastic tetraplegia	Nystagmus	None	GDD + ID	Dystonia	None	Severe hypomielination	None	WES (Reanalysis)	
SPAST	IDSPG9	Female	89	45	YES (three affected generations)	None	AD	Spastic paraparesis	Nystagmus	None	None	None	None	Normal	Wheelchair	WES	
SPAST	IDSPG73	Male	20	3	None	None	AD	Spastic paraparesis	Nystagmus	None	Language disorder	None	None	Myelination delay	None	WES	
SPAST	IDSPG132	Female	36	30	YES (father)	None	AD	Pure spastic paraplegia	None	None	None	None	None	None	None	CNV	
SPAST	IDSPG160	Male	64	49	YES (mother)	None	AD	Pure spastic paraplegia	None	None	None	None	None	Normal	None	WES	
SPG11	IDSPG20	Female	32	28	None	None	AR (Compouund Heterozygous)	Spastic paraparesis	None	None	None	None	None	Periventricular T2 WM HI	Congenital hip luxation, psoriasis.	WES	
SPG11	IDSPG24	Male	19	18	None	None	AR (Compouund Heterozygous)	Spastic paraparesis	None	None	ID	None	None	Periventricular T2 WM HI	None	WES	
SPG11	IDSPG40	Female	25	15	None	None	AR (Compouund Heterozygous)	Spastic paraparesis	None	Peripheral neuropathy	None	None	None	Periventricular T2 WM HI	None	WES	

Gene	ID	Sex	Age	Age at onset	Affected relatives	Consanguinity	Inheritance mode	Pyramidal involvement	Cerebellar involvement	Neuropathy	Cognitive / Behaviour	Extrapiramidal features	Seizures	MRI findings	Other findings (exam or investigation tests)	Diagnostic technique	Treatment
SPG11	IDSPG48	Male	21	2	None	None	AR	Spastic paraparesis	None	Optic neuropathy	GDD + attention disorder	None	None	Periatrial T2 WM HI, mild atrophy, thin CC	None	WES	
SPG11	IDSPG99	Male	41	31	None	None	AR (Homozygous)	Pure spastic paraplegia	None	None	None	None	None	Normal	None	WES	
SPG7	IDSPG21	Male	51	37	None	None	AR (Compouund Heterozygous)	Spastic paraparesis	Ataxia	None	None	None	None	Olivopontocerebellar degeneration	Bilateral ptosis, ophtalmoparesia	WGS	
SPG7	IDSPG23	Female	60	50	YES (sister)	YES	AR (Homozygous)	Pure spastic paraplegia	None	None	None	None	None	Cervical spinal cord atrophy	None	WES	
SPG7	IDSPG30	Male	53	35	YES (two siblings)	YES	AR (Homozygous)	Spastic paraparesis	Ataxia	None	None	None	None	Cerebellar atrophy	Ptosis	WES	
SPG7	IDSPG33	Female	64	40	YES (grandmother)	YES	AR (Homozygous)	Pure spastic paraplegia	None	None	None	None	None	Normal	None	WES	
SPTAN1	IDSPG134	Male	20	7	None	None	AD	Pure spastic paraplegia	None	None	None	None	None	Normal	D8 - D10 syringomyelia	WGS	
SPTBN2	IDSPG125	Male	38	30	None	None	AD	None	Ataxia	None	Executive dysfunction	Dystonia	None	Cerebellar atrophy	None	WGS	
SVBP	IDSPG8	Male	69	16	YES (brother)	None	AR (Homozygous)	Spastic paraparesis	None	None	ID	None	None	Normal	None	WES (Reanalysis)	
SVBP	IDSPG46	Female	37	30	YES (sister)	None	AR (Homozygous)	None	None	Axonal sensorimotor peripheral neuropathy	None	None	None	Dilation of the ventricles and cortical sulci, greater than expected	None	WES (Reanalysis)	
TAF1	IDSPG71	Male	7	0	None	None	X-Linked	Spastic paraparesis	None	None	GDD	None	None	Dysplastic CC, hydrocephalus secondary to aqueductal stenosis, colpocephalia	High lactate and amonium in CSF	WES (Reanalysis)	
TMEM240	IDSPG92	Male	47	3	None	YES	AD	None	Ataxia	None	OCD	Tremor	None	Cerebellar atrophy, specially superior foliae, brainstem atrophy	None	WES	
TRMT5	IDSPG120	Male	16	0	None	None	AR (Compouund Heterozygous)	Spastic paraparesis	None	Distal hereditary motor neuropathy	ID	None	None	Cerebellar atrophy	Abnormal SSEP	WES	Coenzyme Q10, thiamine, monitoring of cardiac manifestations
UBAP1	IDSPG76	Female	14	7	YES (Mother)	None	AD	Spastic paraparesis	None	None	ADHD	None	None	Normal	None	WES (Reanalysis)	

AD Autosomal Dominant
ADHD Attention Deficit and hyperactivity disorder
AR Autosomal Recessive
ASD Autism Spectrum Disease
BCAA Branched-Chain Amino Acids
BG Basal ganglia
CC Corpus callosum
CSF Cerebrospinal fluid
EEG Electroencephalogram
EMG Electromyogram
FDG-PET/CT Fluorodesoxyglucose Positron Emission Tomography / Computerised tomography
GDD Global Developmental Delay
HI Hyperintensity
ID Intellectual Disability
OCD Obsessive-Compulsive Disorder
SSEP Somatosensory-evoked potential
VEP Visual-evoked potential
WM White matter

Table S2. Clinical table. Clinical manifestations, main complementary exams and genes identified in diagnosed cases. Cases in which, after retrospective review, we considered that the clinical diagnosis was possible.

Table S3

Gene	Variant	Patient	Sex	Age	Age of onset	Family History	Main Phenotypical Features	Inheritance	ACMG Classification	Comments
ACER3	NP_060837.3:p.(Gly211Cys)	IDSPG75	M	16	1	YES (affected cousin) consanguinity	Pure spastic paraparesis, early onset. Normal MRI	AR (Homozygous)	PATH	ACER3 causes an early childhood-onset progressive leukodystrophy, patients are severely impaired, needing mechanical ventilation and feeding tube. Milder phenotype with slowly progressive pure spastic paraplegia without white matter involvement.
COL6A3	NP_004360.2:p.(Lys2483Glu)	IDSPG161	F	56	3	NO	Pyramidal signs. Distal hereditary motor neuropathy	AR (Homozygous)	PATH	Atypical form with predominantly distal involvement and the neurophysiological patterns of neurogenic origin.
GFAP	NP_001124491.1:p.(Gly18Val)	IDSPG4	F	61	20	YES (three generations)	Spastic paraparesis, nystagmus. MRI showed mild signal change in brainstem	AD	PATH	This variant, functionally validated in an astrocytoma cell line, might cause variable expressivity and an attenuated phenotype, probably associated with alternative pathogenic mechanisms [24].
KCNA1	NP_000208.2:p.(Val368Leu)	IDLNF52	F	8	0	Consanguinity	Early onset epileptic encephalopathy, dyskinesias, global developmental delay with intelectual disability	AR (Homozygous)	PATH	New inheritance mode with a more severe phenotype.Over 50 families affected by the episodic ataxia type 1 disease spectrum have been described [45].
KIDINS220	NM_020738.3:c.4054-1G>C	IDSPG118	F	10	4	NO	Spastic paraparesis. Motor delay, autonomous ambulation at 4.5 years. Cognitive level preserved	AD	LP	Previously reported: SINO syndrome (spastic paraplegia with intellectual disability, nystagmus, and obesity). Additional features besides spastic paraplegia were not present in this patient.
LONP1	NM_004793.3:c.2154+1G>C<br>NP_004784.2:p.(Leu306Trp)	IDSPG166	F	39	2	YES (affected brother)	Child onset cerebellar ataxia with pyramidal involvement, slowly progressive. MRI: Cerebellar atrophy and dilated 4th ventricle	AR (Compound Heterozygous)	LP/PATH	Previously reported: CODAS syndrome (Cerebral, Ocular, Dental, Auricular, and Skeletal Anomalies). New phenotype, exhibing only cerebellar ataxia.
NDUFS6	NM_004553.4:c.309+5G>A	IDSPG55	F	67	10	YES (affected sister) consanguinity	Spastic paraparesis, axonal sensorimotor peripheral neuropathy, hypoacusia, keratoconus, cataracts, tremor, seizures, vocal cord palsy. MRI: lenticular nuclei hyperintensities	AR (Homozygous)	PATH	Most of patients present with severe neonatal lactic acidemia and complex I deficiency leading to death in the first days of life. Third described case with the same variant, mildest phenotype, first one reaching adult life.
PDK3	NP_001135858.1:p.(Arg158His)	IDSPG172	M	50	13	NO	Spastic paraparesis with congenital severe axonal sensorimotor peripheral neuropathy	X-Linked	PATH	Pyramidal signs not described previously
PMM2	NP_000294.1:p.(Arg141His)<br>NM_000303.2:c.640-23A>G	IDSPG123	M	13	0	NO	Non-progressive congenital ataxia with oculomotor apraxia, hypotonia; neurodevelopmental delay with intellectual disability	AR (Compound Heterozygous)	PATH/PATH	Pure neurologic and mild phenotype without enteropathy, coagulopathy and hepatopathy; furthermore, sialotransferrin profile is normal.
SARS1	NM_006513.4:c.969+1_969+3del	IDSPG64	M	9	0	NO	Spastic paraparesis with ataxia, global developmental delay, intellectual disability and febrile seizures	AD	PATH	New inheritance mode (*de novo* dominant) with a novel complex spastic paraplegia phenotype. This clinical phenotype was reported in a previous publication [46].
SPTAN1	NP_001123910.1:p.(Arg19Trp)	IDSPG134	M	18	7	NO	Pure spastic paraparesis, pediatric onset, pace of progression. Normal MRI	AD	LP	Pure HSP related with biallelic SPTAN1 variants have been described; SPTAN1 heterozygous mutations may cause EIEE5 and distal axonal motor neuropathy with pyramidal involvement. WGS ruled out a second variant, consistent with a pure HSP phenotype associated with a *de novo* heterozygous variant.

M = male; F = female; EIEE5 early infantile epileptic encephalopathy-5; HSP = hereditary spastic paraplegia; LP = Likely pathogenic

Table S3. Atypical cases. Patients with new phenotypes, atypical forms of presentation and blended phenotypes in families with more than one gene associated with the phenotype.

Table S4

ID	Genes	Inheritance	Chr	Start base	End base	Type	Nomenclature	CNV validation
IDSPG116	***FARS2***	Heterozygous	6	5172693	5459957	deletion	6p25.1(5172693-5459957)x1	Q-PCR was carried out to measure the relative copy number of the chromosome 6: 5172693-5459957. Expression level of human *LYRM4* (exon 2-exon 2) and FARS2 gene (intron 1-intron 1 and exon 3-exon 3) were compared to the human *CDLY* (exon 4-exon 4) or NRN1 (exon 2-exon 2) genes. IDSPG116 and father exhibit only one copy of the *FARS2* gene compared to the mother and 8 healthy individuals.
IDSPG132	***SPAST***	Heterozygous	2	32337285	32350543	deletion	2p22.3(32337285-32350543)x1	Q-PCR was carried out to measure the relative copy number of the human SPAST gene (exon 5-exon 5, exon 7-exon 7, and intron 7-intron 7) compared to the human *MEMO1* (exon 2-exon 2) or *NLRC4* (exon 4-exon 4) genes. IDSPG132 and father exhibit only one copy of the *SPAST* gene compared to the mother and 8 healthy individuals.

Table S4. CNV cases. Validation of CNV variants from WGS.

Table S5

											PVS	PS			PM						PP						
**Gene**	**ID**	**Inheritance**	**Type**	**Genomic GRCh37 description**	**Transcript description**	**Protein description**	**Chr**	**VCF Genomic**	**Reference**	**Alternative**	**PVS1**	**PS1**	**PS2**	**PS3**	**PM1**	**PM2**	**PM3**	**PM4**	**PM5**	**PM6**	**PP1**	**PP2**	**PP3**	**PP4**	**PP5**	**CONCLUSION**	**ClinVar accession numbers**
**ACER3**	IDSPG75	AR (Homozygous)	nonsynonymous SNV	NC_000011.9:g.76727750G>T	NM_018367.5:c.631G>T	NP_060837.3:p.(Gly211Cys)	11	76727750	G	T	NO	NO	NO	YES	YES	YES	NO	NO	NO	NO	YES	NO	YES	YES	NO	**PATHOGENIC**	SCV003920739
**AFG3L2**	IDSPG42	AD	nonsynonymous SNV	NC_000018.9:g.12340333T>C	NM_006796.2:c.1847A>G	NP_006787.2:p.(Tyr616Cys)	18	12340333	T	C	NO	NO	NO	YES	NO	YES	NO	NO	YES	NO	NO	NO	YES	YES	YES	**PATHOGENIC**	SCV003920754
**ALS2**	IDSPG74	AR (Homozygous)	nonsynonymous SNV	NC_000002.11:g.202593881T>G	NM_020919.3:c.2606A>C	NP_065970.2:p.(Gln869Pro)	2	202593881	T	G	NO	NO	NO	NO	NO	YES	NO	NO	NO	NO	NO	YES	NO	YES	YES	**LIKELY PATHOGENIC**	SCV003920765
**AMPD2**	IDSPG78	AR (Homozygous)	frameshift deletion	NC_000001.10:g.110168326_110168338del	NM_139156.3:c.184_196del	NP_631895.1:p.(Ala62SerfsTer40)	1	110168322	TAGTGCC CCGTATG	T	YES	NO	NO	NO	NO	YES	NO	NO	NO	NO	NO	NO	YES	YES	NO	**PATHOGENIC**	SCV003920776
**AP4B1**	IDSPG167	AR (Compound Heterozygous)	nonsynonymous SNV	NC_000001.10:g.114438613G>A	NM_001253852.1:c.1558C>T	NP_001240781.1:p.(Arg520Cys)	1	114438613	G	A	NO	NO	NO	NO	NO	YES	YES	NO	NO	NO	NO	NO	YES	YES	YES	**LIKELY PATHOGENIC**	SCV003920787
**AP4B1**	IDSPG167	AR (Compound Heterozygous)	nonsynonymous SNV	NC_000001.10:g.114443898C>T	NM_001253852.1:c.577G>A	NP_001240781.1:p.(Val193Ile)	1	114443898	C	T	NO	YES	NO	NO	YES	YES	NO	NO	NO	NO	NO	NO	YES	YES	YES	**PATHOGENIC**	SCV003920798
**ATL1**	IDSPG168	AD	nonsynonymous SNV	NC_000014.8:g.51058301A>C	NM_181598.3:c.466A>C	NP_853629.2:p.(Thr156Pro)	14	51058301	A	C	NO	NO	YES	NO	YES	YES	NO	NO	YES	NO	NO	NO	YES	YES	YES	**PATHOGENIC**	SCV003920809
**ATL1**	IDSPG5	AD	nonsynonymous SNV	NC_000014.8:g.51095112C>T	NM_015915.4:c.1483C>T	NP_056999.2:p.(Arg495Trp)	14	51095112	C	T	NO	NO	NO	YES	NO	YES	NO	NO	NO	NO	YES	NO	YES	YES	YES	**PATHOGENIC**	SCV003920820
**ATL1**	IDSPG70	AD	nonsynonymous SNV	NC_000014.8:g.51080061C>T	NM_015915.4:c.715C>T	NP_056999.2:p.(Arg239Cys)	14	51080061	C	T	NO	NO	NO	YES	NO	YES	NO	NO	NO	NO	YES	NO	YES	YES	YES	**PATHOGENIC**	SCV003920831
**BCKDK**	IDSPG47.0	AR (Homozygous)	nonsynonymous SNV	NC_000016.9:g.31123233C>T	NM_005881.3:c.979C>T	NP_005872.2:p.(Arg327Trp)	16	31123233	C	T	NO	NO	NO	NO	YES	YES	NO	NO	NO	NO	NO	NO	YES	YES	NO	**LIKELY PATHOGENIC**	SCV003920740
**BSCL2**	IDSPG112	AD	nonsynonymous SNV	NC_000011.9:g.62469971T>C	NM_032667.6:c.263A>G	NP_116056.3:p.(Asn88Ser)	11	62469971	T	C	NO	NO	NO	YES	YES	YES	NO	NO	NO	NO	YES	NO	YES	YES	YES	**PATHOGENIC**	SCV003920745
**BSCL2**	IDSPG145	AD	nonsynonymous SNV	NC_000011.9:g.62469971T>C	NM_032667.6:c.263A>G	NP_116056.3:p.(Asn88Ser)	11	62469971	T	C	NO	NO	NO	YES	YES	YES	NO	NO	NO	NO	YES	NO	YES	YES	YES	**PATHOGENIC**	SCV003920745
**BSCL2**	IDSPG157	AD	nonsynonymous SNV	NC_000011.9:g.62469971T>C	NM_032667.6:c.263A>G	NP_116056.3:p.(Asn88Ser)	11	62469971	T	C	NO	NO	NO	YES	YES	YES	NO	NO	NO	NO	NO	NO	YES	YES	YES	**PATHOGENIC**	SCV003920745
**BSCL2**	IDSPG29	AD	nonsynonymous SNV	NC_000011.9:g.62469971T>C	NM_032667.6:c.263A>G	NP_116056.3:p.(Asn88Ser)	11	62469971	T	C	NO	NO	NO	YES	YES	YES	NO	NO	NO	NO	NO	NO	YES	YES	YES	**PATHOGENIC**	SCV003920745
**CACNA1A**	IDLNF56	AD	stopgain	NC_000019.9:g.13423517dup	NM_001127221.1:c.1637dup	NP_001120693.1:p.(Tyr546Ter)	19	13423516	G	GT	YES	NO	YES	NO	NO	YES	NO	NO	NO	NO	NO	NO	NO	YES	YES	**PATHOGENIC**	SCV003920746
**CACNA1A**	IDSPG126	AD	nonsynonymous SNV	NC_000019.9:g.13370469G>A	NM_001127222.1:c.4297C>T	NP_001120694.1:p.(Arg1433Trp)	19	13370469	G	A	NO	NO	NO	NO	NO	YES	NO	NO	NO	NO	NO	YES	YES	YES	YES	**VUS**	SCV003920747
**CAPN1**	IDSPG2	AR (Homozygous)	splicing	NC_000011.9:g.64974295G>A	NM_001198868.1:c.1605+5G>A	NP_001185797.1:p.?	11	64974295	G	A	YES	NO	NO	YES	NO	YES	NO	NO	NO	NO	YES	NO	YES	NO	YES	**PATHOGENIC**	SCV003920748
**CAPN10**	IDSPG47.1	AR (Homozygous)	splicing	NC_000002.11:g.241537815G>A	NM_023083.3:c.1989+1G>A	NP_075571.1:p.?	2	241537815	G	A	YES	NO	NO	NO	NO	YES	NO	NO	NO	NO	NO	NO	YES	YES	NO	**PATHOGENIC**	SCV003920749
**CC2D2A**	IDLNF129	AR (Compound Heterozygous)	stopgain	NC_000004.11:g.15565018C>T	NM_001080522.2:c.3055C>T	NP_001073991.2:p.(Arg1019Ter)	4	15565018	C	T	YES	NO	NO	NO	NO	YES	NO	NO	NO	NO	NO	NO	YES	YES	YES	**PATHOGENIC**	SCV003920750
**CC2D2A**	IDLNF129	AR (Compound Heterozygous)	nonsynonymous SNV	NC_000004.11:g.15601322A>T	NM_001080522.2:c.4667A>T	NP_001073991.2:p.(Asp1556Val)	4	15601322	A	T	NO	NO	NO	NO	NO	YES	YES	NO	NO	NO	NO	NO	YES	YES	YES	**LIKELY PATHOGENIC**	SCV003920751
**COL6A3**	IDSPG161	AR (Homozygous)	nonsynonymous SNV	NC_000002.11:g.238253214T>C	NM_004369.4:c.7447A>G	NP_004360.2:p.(Lys2483Glu)	2	238253214	T	C	NO	NO	NO	NO	NO	YES	NO	NO	NO	NO	NO	NO	YES	YES	YES	**LIKELY PATHOGENIC**	SCV003920752
**CTNNB1**	IDSPG139	AD	frameshift deletion	NC_000003.11:g.41277961_41277962del	NM_001904.3:c.1925_1926del	NP_001895.1:p.(Glu642ValfsTer5)	3	41277958	CAG	C	YES	NO	YES	NO	NO	YES	NO	NO	NO	NO	NO	NO	NO	YES	YES	**PATHOGENIC**	SCV003920753
**CYP2U1**	IDLNF40	AR (Homozygous)	nonsynonymous SNV	NC_000004.11:g.108866168G>C	NM_183075.2:c.533G>C	NP_898898.1:p.(Arg178Thr)	4	108866168	G	C	NO	NO	NO	NO	NO	YES	NO	NO	NO	NO	YES	YES	YES	YES	NO	**LIKELY PATHOGENIC**	SCV003920755
**DDHD2**	IDSPG61	AR (Homozygous)	stopgain	NC_000008.10:g.38103267C>T	NM_001164232.1:c.856C>T	NP_001157704.1:p.(Gln286Ter)	8	38103267	C	T	YES	NO	NO	NO	NO	YES	NO	NO	NO	NO	NO	NO	NO	YES	YES	**PATHOGENIC**	SCV003920756
**DLG4**	IDSPG107	AD	splicing	NC_000017.10:g.7096904C>T	NM_001365.4:c.1721-1G>A	NP_001356.1:p.?	17	7096904	C	T	YES	NO	YES	NO	NO	YES	NO	NO	NO	NO	NO	NO	YES	YES	NO	**PATHOGENIC**	SCV003920757
**DSTYK**	IDLNF84	AR (Homozygous)	splicing	NC_000001.10:g.205156545C>T	NM_015375.2:c.654+1G>A	NP_056190.1:p.?	1	205156545	C	T	YES	NO	NO	NO	NO	YES	NO	NO	NO	NO	NO	NO	YES	NO	YES	**PATHOGENIC**	SCV003920758
**ERBB4**	IDSPG38	AD	non-canonical splicing	NC_000002.11:g.212426619_212426622del	NM_005235.2:c.2487+8_2487+11del	NP_005226.1:p.?	2	212426616	ACAGG	A	NO	NO	NO	NO	NO	YES	NO	NO	NO	NO	NO	NO	YES	YES	NO	**VUS**	SCV003920759
**FA2H**	IDSPG10	AR (Homozygous)	nonsynonymous SNV	NC_000016.9:g.74752887T>G	NM_024306.4:c.785A>C	NP_077282.3:p.(Lys262Thr)	16	74752887	T	G	NO	NO	NO	NO	YES	YES	NO	NO	NO	NO	NO	NO	YES	YES	NO	**LIKELY PATHOGENIC**	SCV003920760
**FARS2**	IDSPG116	AR (Compound Heterozygous)	nonsynonymous SNV	5613418C>T	NM_006567.5:c.1082C>T	NP_006558.1:p.(Pro361Leu)	6	5613418	C	T	NO	NO	NO	NO	NO	YES	YES	NO	NO	NO	NO	NO	YES	YES	YES	**PATHOGENIC**	SCV003920761
**FARS2**	IDSPG116	AR (Compound Heterozygous)	deletion	6p25.1(5172693-5459957)x1			6	5172693			YES	NO	NO	NO	YES	NO	NO	NO	NO	NO	NO	NO	NO	YES	NO	**PATHOGENIC**	SCV003920762
**GFAP**	IDSPG4	AD	nonsynonymous SNV	NC_000017.10:g.42992802C>A	NM_001131019.2:c.53G>T	NP_001124491.1:p.(Gly18Val)	17	42992802	C	A	NO	NO	NO	YES	NO	YES	NO	NO	NO	NO	YES	NO	NO	YES	NO	**PATHOGENIC**	SCV003920763
**IFIH1**	IDSPG3	AD	nonsynonymous SNV	NC_000002.11:g.163144780C>G	NM_022168.4:c.960G>C	NP_071451.2:p.(Leu320Phe)	2	163144780	C	G	NO	NO	NO	NO	YES	YES	NO	NO	NO	NO	NO	NO	YES	NO	NO	**VUS**	SCV003920764
**IFIH1**	IDSPG60	AD	nonsynonymous SNV	NC_000002.11:g.163174445G>C	NM_022168.4:c.373C>G	NP_071451.2:p.(Leu125Val)	2	163174445	G	C	NO	NO	NO	NO	NO	YES	NO	NO	YES	NO	NO	NO	YES	NO	YES	**VUS**	SCV003920766
**IQSEC2**	IDSPG127	X-Linked	nonsynonymous SNV	NC_000023.10:g.53265648G>A	NM_001111125.3:c.3307C>T	NP_001104595.1:p.(Arg1103Trp)	X	.53265648	G	A	NO	NO	NO	NO	NO	YES	NO	NO	NO	NO	NO	NO	YES	YES	YES	**VUS**	SCV003920767
**IRF2BPL**	IDLNF58	AD	stopgain	NC_000014.8:g.77493574G>A	NM_024496.3:c.562C>T	NP_078772.1:p.(Arg188Ter)	14	77493574	G	A	YES	NO	NO	YES	NO	YES	NO	NO	NO	YES	NO	NO	YES	YES	YES	**PATHOGENIC**	SCV003920768

Gene	ID	Inheritance	Type	Genomic GRCh37 description	Transcript description	Protein description	Chr	VCF Genomic	Reference	Alternative	PVS1	PS1	PS2	PS3	PM1	PM2	PM3	PM4	PM5	PM6	PP1	PP2	PP3	PP4	PP5	CONCLUSION	ClinVar accession numbers
**IRF2BPL**	IDSPG44	AD	stopgain	NC_000014.8:g.77493574G>A	NM_024496.3:c.562C>T	NP_078772.1:p.(Arg188Ter)	14	77493574	G	A	YES	NO	NO	YES	NO	YES	NO	NO	NO	YES	NO	NO	YES	YES	YES	**PATHOGENIC**	SCV003920768
**KCNA1**	IDLNF52	AR (Homozygous)	nonsynonymous SNV	NC_000012.11:g.5021646G>C	NM_000217.2:c.1102G>C	NP_000208.2:p.(Val368Leu)	12	5021646	G	C	NO	NO	NO	YES	YES	YES	NO	NO	NO	NO	NO	YES	YES	YES	NO	**PATHOGENIC**	SCV003920769
**KIDINS220**	IDSPG118	AD	splicing	NC_000002.11:g.8872113C>G	NM_020738.3:c.4054-1G>C	NP_065789.1:p.?	2	8872113	C	G	NO	NO	NO	YES	NO	YES	NO	NO	NO	NO	NO	NO	YES	YES	NO	**LIKELY PATHOGENIC**	SCV003920770
**KIF1A**	IDSPG108	AD	nonsynonymous SNV	NC_000002.11:g.241722504G>A	NM_004321.7:c.821C>T	NP_004312.2:p.(Ser274Leu)	2	241722504	G	A	NO	NO	YES	NO	YES	YES	NO	NO	NO	NO	NO	YES	YES	YES	YES	**PATHOGENIC**	SCV003920771
**KIF1A**	IDSPG110	AD	nonsynonymous SNV	NC_000002.11:g.241723181G>A	NM_004321.7:c.773C>T	NP_004312.2:p.(Thr258Met)	2	241723181	G	A	NO	NO	NO	NO	YES	YES	NO	NO	NO	NO	NO	YES	YES	YES	YES	**LIKELY PATHOGENIC**	SCV003920772
**KIF1A**	IDSPG39	AD	nonsynonymous SNV	NC_000002.11:g.241723193C>T	NM_004321.7:c.761G>A	NP_004312.2:p.(Arg254Gln)	2	241723193	C	T	NO	NO	YES	NO	YES	YES	NO	NO	YES	NO	NO	YES	YES	YES	YES	**PATHOGENIC**	SCV003920773
**KIF5A**	IDSPG17	AD	nonsynonymous SNV	NC_000012.11:g.57962768G>T	NM_004984.2:c.737G>T	NP_004975.2:p.(Gly246Val)	12	57962768	G	T	NO	NO	YES	NO	YES	YES	NO	NO	NO	NO	NO	YES	YES	YES	NO	**PATHOGENIC**	SCV003920774
**KMT2B**	IDSPG114	AD	nonsynonymous SNV	NC_000019.9:g.36221345G>T	NM_014727.2:c.5179G>T	NP_055542.1:p.(Ala1727Ser)	19	36221345	G	T	NO	NO	NO	NO	YES	YES	NO	NO	NO	YES	NO	YES	NO	YES	NO	**LIKELY PATHOGENIC**	SCV003920775
**L2HGDH**	IDSPG47.1	AR (Homozygous)	nonsynonymous SNV	NC_000014.8:g.50750660C>A	NM_024884.2:c.632G>T	NP_079160.1:p.(Gly211Val)	14	50750660	C	A	NO	NO	NO	NO	NO	YES	NO	NO	NO	NO	NO	NO	YES	YES	YES	**VUS**	SCV003920777
**LAMA1**	IDSPG56	AR (Compound Heterozygous)	frameshift insertion	NC_000018.9:g.6948418_6948422dup	NM_005559.3:c.8691_8695dup	NP_005550.2:p.(Gly2899GlufsTer18)	18	6948416	C	CCGCTT	YES	NO	NO	NO	NO	YES	NO	NO	NO	NO	NO	NO	NO	YES	NO	**PATHOGENIC**	SCV003920778
**LAMA1**	IDSPG56	AR (Compound Heterozygous)	non-canonical splicing	NC_000018.9:g.7038961G>C	NM_005559.3:c.1423-12C>G	NP_005550.2:p.?	18	7038961	G	C	NO	NO	NO	YES	NO	YES	YES	YES	NO	NO	NO	NO	YES	YES	NO	**PATHOGENIC**	SCV003920779
**LONP1**	IDSPG166	AR (Compound Heterozygous)	splicing	NC_000019.9:g.5694771C>G	NM_004793.3:c.2154+1G>C	NP_004784.2:p.?	19	5694771	C	G	YES	NO	NO	NO	NO	YES	NO	NO	NO	NO	YES	NO	YES	YES	NO	**PATHOGENIC**	SCV003920780
**LONP1**	IDSPG166	AR (Compound Heterozygous)	nonsynonymous SNV	NC_000019.9:g.5708368A>C	NM_004793.3:c.917T>G	NP_004784.2:p.(Leu306Trp)	19	5708368	A	C	NO	NO	NO	NO	NO	YES	YES	NO	NO	NO	YES	NO	YES	YES	NO	**LIKELY PATHOGENIC**	SCV003920781
**MMUT**	IDLNF84	AR (Homozygous)	nonsynonymous SNV	NC_000006.11:g.49425727G>A	NM_000255.3:c.430C>T	NP_000246.2:p.(Arg144Cys)	6	49425727	G	A	NO	NO	NO	YES	YES	YES	NO	NO	NO	NO	NO	NO	YES	YES	NO	**PATHOGENIC**	SCV003920782
**MORC2**	IDSPG37	AD	nonsynonymous SNV	NC_000022.10:g.31337490G>A	NM_014941.1:c.568C>T	NP_055756.1:p.(Arg190Trp)	22	31337490	G	A	NO	NO	YES	NO	YES	YES	NO	NO	NO	NO	NO	YES	YES	YES	YES	**PATHOGENIC**	SCV003920783
**NDUFS6**	IDSPG55	AR (Homozygous)	splicing	NC_000005.9:g.1814580G>A	NM_004553.4:c.309+5G>A	NP_004544.1:p.?	5	1814580	G	A	YES	NO	NO	YES	NO	YES	NO	NO	NO	NO	NO	NO	YES	YES	YES	**PATHOGENIC**	SCV003920784
**PAX6**	IDSPG155	AD	stopgain	NC_000011.9:g.31815335G>A	NM_001310160.1:c.373C>T	NP_001297089.1:p.(Arg125Ter)	11	31815335	G	A	YES	NO	NO	NO	NO	YES	NO	NO	NO	NO	NO	NO	YES	YES	YES	**PATHOGENIC**	SCV003920785
**PCYT2**	IDSPG27	AR (Homozygous)	nonsynonymous SNV + splicing	NC_000017.10:g.79863546C>G	NM_001184917.2:c.957G>C	NP_001171846.1:p.(Lys319Asn)	17	79863546	C	G	NO	NO	NO	YES	NO	YES	NO	YES	NO	NO	NO	NO	NO	YES	NO	**LIKELY PATHOGENIC**	SCV003920786
**PDK3**	IDSPG172	X-Linked	nonsynonymous SNV	NC_000023.10:g.24521596G>A	NM_001142386.2:c.473G>A	NP_001135858.1:p.(Arg158His)	X	24521596	G	A	NO	NO	YES	YES	YES	YES	NO	NO	NO	NO	NO	NO	YES	YES	YES	**PATHOGENIC**	SCV003920788
**PI4KA**	IDSPG149	AR (Compound Heterozygous)	nonsynonymous SNV	NC_000022.10:g.21083617C>T	NM_058004.3:c.4666G>A	NP_477352.3:p.(Val1556Met)	22	21083617	C	T	NO	NO	NO	YES	YES	YES	NO	NO	NO	NO	NO	NO	YES	YES	NO	**PATHOGENIC**	SCV003920789
**PI4KA**	IDSPG149	AR (Compound Heterozygous)	nonsynonymous SNV	NC_000022.10:g.21073068G>A	NM_058004.3:c.5159C>T	NP_477352.3:p.(Thr1720Ile)	22	21073068	G	A	NO	NO	NO	YES	YES	YES	YES	NO	NO	NO	NO	NO	NO	YES	NO	**PATHOGENIC**	SCV003920790
**PI4KA**	IDSPG16	AR (Compound Heterozygous)	frameshift deletion	NC_000022.10:g.21064212_21064215del	NM_058004.3:c.6156_6159del	NP_477352.3:p.(Thr2053SerfsTer4)	22	21064209	TTGTC	T	YES	NO	NO	YES	NO	YES	YES	NO	NO	NO	NO	NO	YES	YES	NO	**PATHOGENIC**	SCV003920791
**PI4KA**	IDSPG16	AR (Compound Heterozygous)	frameshift deletion	NC_000022.10:g.21068749_21068751del	NM_058004.3:c.5459_5461del	NP_477352.3:p.(Glu1820del)	22	21068745	CCTT	C	YES	NO	NO	YES	YES	YES	NO	YES	NO	NO	NO	NO	YES	YES	NO	**PATHOGENIC**	SCV003920792
**PMM2**	IDSPG123	AR (Compound Heterozygous)	nonsynonymous SNV	NC_000016.9:g.8905010G>A	NM_000303.2:c.422G>A	NP_000294.1:p.(Arg141His)	16	8905010	G	A	NO	NO	NO	NO	YES	YES	NO	NO	YES	NO	NO	NO	YES	YES	YES	**PATHOGENIC**	SCV003920793
**PMM2**	IDSPG123	AR (Compound Heterozygous)	non-canonical splicing	NC_000016.9:g.8941558A>G	NM_000303.2:c.640-23A>G	NP_000294.1:p.?	16	8941558	A	G	NO	NO	NO	YES	NO	YES	YES	NO	NO	NO	NO	NO	NO	YES	YES	**PATHOGENIC**	SCV003920794
**PNKP**	IDSPG42	AR (Compound Heterozygous)	nonsynonymous SNV	NC_000019.9:g.50365360C>T	NM_007254.3:c.1129G>A	NP_009185.2:p.(Gly377Arg)	19	50365360	C	T	NO	NO	NO	NO	YES	YES	YES	NO	NO	NO	NO	NO	YES	YES	YES	**LIKELY PATHOGENIC**	SCV003920795
**PNKP**	IDSPG42	AR (Compound Heterozygous)	nonsynonymous SNV	NC_000019.9:g.50365445C>A	NM_007254.3:c.1123G>T	NP_009185.2:p.(Gly375Trp)	19	50365445	C	A	NO	NO	NO	NO	YES	YES	NO	NO	NO	NO	NO	NO	YES	YES	YES	**PATHOGENIC**	SCV003920796
**PNPLA6**	IDSPG13	AR (Compound Heterozygous)	splicing	NC_000019.9:g.7605514A>C	NM_006702.4:c.598-2A>C	NP_006693.3:p.?	19	7605514	A	C	YES	NO	NO	NO	NO	YES	NO	NO	NO	NO	NO	NO	YES	YES	NO	**PATHOGENIC**	SCV003920797
**PNPLA6**	IDSPG13	AR (Compound Heterozygous)	nonsynonymous SNV	NC_000019.9:g.7623750A>T	NM_001166114.1:c.3412A>T	NP_001159586.1:p.(Ser1138Cys)	19	7623750	A	T	NO	NO	NO	NO	YES	YES	YES	NO	NO	NO	NO	YES	YES	YES	NO	**LIKELY PATHOGENIC**	SCV003920799
**POLG**	IDSPG113	AR (Compound Heterozygous)	nonsynonymous SNV	NC_000015.9:g.89872286A>C	NM_002693.2:c.911T>G	NP_002684.1:p.(Leu304Arg)	15	89872286	A	C	NO	NO	NO	NO	YES	YES	NO	NO	NO	NO	NO	NO	YES	YES	YES	**PATHOGENIC**	SCV003920800
**POLG**	IDSPG113	AR (Compound Heterozygous)	non-canonical splicing	NC_000015.9:g.89866197G>A	NM_002693.2:c.2266-64C>T	NP_002684.1:p.?	15	89866197	G	A	NO	NO	NO	NO	NO	YES	YES	NO	NO	NO	NO	NO	NO	YES	NO	**VUS**	SCV003920801
**POLR3A**	IDLNF56	AR (Compound Heterozygous)	nonsynonymous SNV	NC_000010.10:g.79764550C>T	NM_007055.3:c.2171G>A	NP_008986.2:p.(Cys724Tyr)	10	79764550	C	T	NO	NO	NO	NO	NO	YES	NO	NO	NO	NO	NO	NO	YES	YES	YES	**PATHOGENIC**	SCV003920802
**POLR3A**	IDLNF56	AR (Compound Heterozygous)	nonsynonymous SNV	NC_000010.10:g.79764608G>C	NM_007055.3:c.2113C>G	NP_008986.2:p.(Pro705Ala)	10	79764608	G	C	NO	NO	NO	NO	NO	YES	YES	NO	NO	NO	NO	NO	YES	YES	NO	**LIKELY PATHOGENIC**	SCV003920803
**POLR3A**	IDSPG109	AR (Homozygous)	non-canonical splicing	NC_000010.10:g.79769440G>C	NM_007055.3:c.1771-7C>G	NP_008986.2:p.?	10	79769440	G	C	YES	NO	NO	YES	NO	YES	NO	NO	NO	NO	NO	NO	YES	YES	YES	**PATHOGENIC**	SCV003920804
**POLR3A**	IDSPG122	AR (Compound Heterozygous)	nonsynonymous SNV	NC_000010.10:g.79741203C>T	NM_007055.3:c.3874G>A	NP_008986.2:p.(Asp1292Asn)	10	79741203	C	T	NO	NO	NO	NO	NO	YES	YES	NO	NO	NO	NO	NO	YES	YES	YES	**LIKELY PATHOGENIC**	SCV003920805
**POLR3A**	IDSPG122	AR (Compound Heterozygous)	non-canonical splicing	NC_000010.10:g.79769273C>T	NM_007055.3:c.1909+22G>A	NP_008986.2:p.?	10	79769273	C	T	YES	NO	NO	YES	NO	YES	NO	NO	NO	NO	NO	NO	YES	YES	YES	**PATHOGENIC**	SCV003920806

Gene	ID	Inheritance	Type	Genomic GRCh37 description	Transcript description	Protein description	Chr	VCF Genomic	Reference	Alternative	PVS1	PS1	PS2	PS3	PM1	PM2	PM3	PM4	PM5	PM6	PP1	PP2	PP3	PP4	PP5	CONCLUSION	ClinVar accession numbers
**POLR3A**	IDSPG14	AR (Compound Heterozygous)	non-canonical splicing	NC_000010.10:g.79769273C>T	NM_007055.3:c.1909+22G>A	NP_008986.2:p.?	10	79769273	C	T	YES	NO	NO	YES	NO	YES	NO	NO	NO	NO	YES	NO	NO	YES	YES	**PATHOGENIC**	SCV003920806
**POLR3A**	IDSPG14	AR (Compound Heterozygous)	nonsynonymous SNV	NC_000010.10:g.79785447C>T	NM_007055.3:c.251G>A	NP_008986.2:p.(Gly84Glu)	10	79785447	C	T	NO	NO	NO	NO	NO	YES	YES	NO	NO	NO	YES	YES	YES	YES	NO	**LIKELY PATHOGENIC**	SCV003920807
**POLR3A**	IDSPG28	AR (Compound Heterozygous)	non-canonical splicing	NC_000010.10:g.79769273C>T	NM_007055.3:c.1909+22G>A	NP_008986.2:p.?	10	79769273	C	T	YES	NO	NO	YES	NO	YES	NO	NO	NO	NO	NO	NO	YES	YES	YES	**PATHOGENIC**	SCV003920806
**POLR3A**	IDSPG28	AR (Compound Heterozygous)	nonsynonymous SNV	NC_000010.10:g.79769342C>T	NM_007055.3:c.1862G>A	NP_008986.2:p.(Gly621Asp)	10	79769342	C	T	NO	NO	NO	NO	NO	YES	YES	NO	NO	NO	NO	NO	YES	YES	YES	**LIKELY PATHOGENIC**	SCV003920808
**POLR3B**	IDSPG66	AD	nonsynonymous SNV	NC_000012.11:g.106760479C>G	NM_018082.5:c.206C>G	NP_060552.4:p.(Ala69Gly)	12	106760479	C	G	NO	NO	NO	NO	NO	YES	NO	NO	NO	NO	NO	YES	YES	YES	NO	**VUS**	SCV003920810
**REEP1**	IDSPG12	AD	nonsynonymous SNV	NC_000002.11:g.86491094A>T	NM_022912.2:c.176T>A	NP_075063.1:p.(Leu59His)	2	86491094	A	T	NO	NO	NO	NO	YES	YES	NO	NO	YES	NO	NO	NO	YES	YES	NO	**LIKELY PATHOGENIC**	SCV003920811
**REEP1**	IDSPG124	AD	stopgain	NC_000002.11:g.86479160G>A	NM_022912.2:c.337C>T	NP_075063.1:p.(Arg113Ter)	2	86479160	G	A	YES	NO	NO	YES	NO	YES	NO	NO	NO	NO	NO	NO	YES	YES	YES	**PATHOGENIC**	SCV003920812
**RNASEH2B**	IDLNF141	AR (Homozygous)	nonsynonymous SNV	NC_000013.10:g.51519581G>A	NM_024570.3:c.529G>A	NP_078846.2:p.(Ala177Thr)	13	51519581	G	A	NO	NO	NO	YES	NO	YES	NO	NO	NO	NO	NO	NO	YES	YES	YES	**PATHOGENIC**	SCV003920813
**SARS1**	IDSPG64	AD	splicing	NC_000001.10:g.109778054_109778056del	NM_006513.4:c.969+1_969+3del	NP_006504.2:p.?	1	109778052	AGGT	A	YES	NO	YES	YES	NO	YES	NO	NO	NO	NO	NO	NO	YES	NO	NO	**PATHOGENIC**	SCV003920814
**SEPSECS**	IDLNF121	AR (Homozygous)	non-canonical splicing	NC_000004.11:g.25161875T>C	NM_016955.3:c.114+3A>G	NP_058651.3:p.?	4	25161875	T	C	YES	NO	NO	YES	NO	YES	NO	NO	NO	NO	YES	NO	YES	YES	YES	**PATHOGENIC**	SCV003920815
**SEPSECS**	IDLNF67	AR (Homozygous)	non-canonical splicing	NC_000004.11:g.25161875T>C	NM_016955.3:c.114+3A>G	NP_058651.3:p.?	4	25161875	T	C	YES	NO	NO	YES	NO	YES	NO	NO	NO	NO	NO	NO	YES	YES	YES	**PATHOGENIC**	SCV003920815
**SHMT2**	IDSPG26	AR (Homozygous)	nonsynonymous SNV	NC_000012.11:g.57628124C>G	NM_005412.5:c.1495C>G	NP_005403.2:p.(Pro499Ala)	12	57628124	C	G	NO	NO	NO	YES	NO	YES	NO	NO	NO	NO	YES	NO	YES	YES	NO	**LIKELY PATHOGENIC**	SCV003920816
**SLC25A46**	IDSPG93	AR (Homozygous)	nonsynonymous SNV	NC_000005.9:g.110097243C>T	NM_138773.3:c.1018C>T	NP_620128.1:p.(Arg340Cys)	5	110097243	C	T	NO	NO	NO	YES	YES	YES	NO	NO	YES	NO	NO	YES	NO	YES	YES	**PATHOGENIC**	SCV003920817
**SLC35B2**	IDLNF68	AR (Homozygous)	frameshift deletion	NC_000006.12:g.44222516ACT>A	NM_178148.4:c.1224_1225delAG	NP_835361.1:p.Arg408SerfsTer18	6	44222516	ACT	A	YES	NO	NO	YES	NO	YES	NO	NO	NO	NO	NO	NO	NO	NO	NO	**PATHOGENIC**	SCV003920818
**SPAST**	IDSPG132	AD	deletion	2p22.3(32337285-32350543)x1			2	32337285			YES	NO	NO	NO	YES	NO	NO	NO	NO	NO	YES	NO	NO	YES	NO	**PATHOGENIC**	SCV003920819
**SPAST**	IDSPG160	AD	nonsynonymous SNV	NC_000002.11:g.32366986C>T	NM_014946.3:c.1507C>T	NP_055761.2:p.(Arg503Trp)	2	32366986	C	T	NO	NO	NO	NO	YES	YES	NO	NO	NO	NO	NO	NO	YES	YES	YES	**LIKELY PATHOGENIC**	SCV003920821
**SPAST**	IDSPG73	AD	nonsynonymous SNV	NC_000002.11:g.32361662C>G	NM_014946.3:c.1276C>G	NP_055761.2:p.(Leu426Val)	2	32361662	C	G	NO	NO	NO	YES	YES	YES	NO	NO	YES	NO	NO	NO	YES	YES	YES	**PATHOGENIC**	SCV003920822
**SPAST**	IDSPG9	AD	splicing	NC_000002.11:g.32362038G>A	NM_014946.3:c.1413+1G>A	NP_055761.2:p.?	2	32362038	G	A	YES	NO	NO	NO	NO	YES	NO	NO	NO	NO	YES	NO	YES	YES	YES	**PATHOGENIC**	SCV003920823
**SPG11**	IDSPG20	AR (Compound Heterozygous)	stopgain	NC_000015.9:g.44856746T>A	NM_001160227.1:c.6811A>T	NP_001153699.1:p.(Lys2271Ter)	15	44856746	T	A	NO	NO	NO	NO	NO	YES	YES	YES	NO	NO	NO	NO	NO	YES	YES	**LIKELY PATHOGENIC**	SCV003920824
**SPG11**	IDSPG20	AR (Compound Heterozygous)	non-canonical splicing	NC_000015.9:g.44862719T>C	NM_001160227.1:c.6138+4A>G	NP_001153699.1:p.?	15	44862719	T	C	YES	NO	NO	YES	NO	YES	NO	NO	NO	NO	NO	NO	YES	YES	YES	**PATHOGENIC**	SCV003920825
**SPG11**	IDSPG24	AR (Compound Heterozygous)	stopgain	NC_000015.9:g.44876685C>T	NM_001160227.1:c.5193G>A	NP_001153699.1:p.(Trp1731Ter)	15	44876685	C	T	YES	NO	NO	NO	NO	YES	NO	NO	NO	NO	NO	NO	NO	YES	NO	**PATHOGENIC**	SCV003920826
**SPG11**	IDSPG24	AR (Compound Heterozygous)	frameshift deletion	NC_000015.9:g.44949428_44949429del	NM_001160227.1:c.733_734del	NP_001153699.1:p.(Met245ValfsTer2)	15	44949427	CAT	C	YES	NO	NO	NO	NO	YES	YES	NO	NO	NO	NO	NO	NO	YES	YES	**PATHOGENIC**	SCV003920827
**SPG11**	IDSPG40	AR (Compound Heterozygous)	frameshift insertion	NC_000015.9:g.44859639dup	NM_001160227.1:c.6399dup	NP_001153699.1:p.(Glu2134Ter)	15	44859637	C	CA	YES	NO	NO	NO	NO	YES	NO	NO	NO	NO	NO	NO	NO	YES	NO	**PATHOGENIC**	SCV003920828
**SPG11**	IDSPG40	AR (Compound Heterozygous)	stopgain	NC_000015.9:g.44912518C>A	NM_001160227.1:c.2704G>T	NP_001153699.1:p.(Glu902Ter)	15	44912518	C	A	YES	NO	NO	NO	NO	YES	YES	NO	NO	NO	NO	NO	NO	YES	NO	**PATHOGENIC**	SCV003920829
**SPG11**	IDSPG48	AR (Homozygous)	frameshift deletion	NC_000015.9:g.44949428_44949429del	NM_001160227.1:c.733_734del	NP_001153699.1:p.(Met245ValfsTer2)	15	44949427	CAT	C	YES	NO	NO	NO	NO	YES	NO	NO	NO	NO	NO	NO	NO	YES	YES	**PATHOGENIC**	SCV003920827
**SPG11**	IDSPG99	AR (Compound Heterozygous)	splicing	NC_000015.9:g.44876011C>T	NM_025137.3:c.5866+1G>A	NP_079413.3:p.?	15	44876011	C	T	YES	NO	NO	YES	NO	YES	NO	NO	NO	NO	NO	NO	NO	YES	YES	**PATHOGENIC**	SCV003920830
**SPG11**	IDSPG99	AR (Compound Heterozygous)	frameshift deletion	NC_000015.9:g.44943705del	NM_001160227.1:c.1440del	NP_001153699.1:p.(Cys481AlafsTer4)	15	44943704	AC	A	YES	NO	NO	NO	NO	YES	YES	NO	NO	NO	NO	NO	NO	YES	YES	**PATHOGENIC**	SCV003920832
**SPG7**	IDSPG21	AR (Compound Heterozygous)	nonsynonymous SNV	NC_000016.9:g.89623308T>C	NM_003119.2:c.2195T>C	NP_003110.1:p.(Leu732Pro)	16	89623308	T	C	NO	NO	NO	YES	YES	YES	NO	NO	NO	NO	NO	NO	YES	YES	NO	**PATHOGENIC**	SCV003920833
**SPG7**	IDSPG21	AR (Compound Heterozygous)	non-canonical splicing	NC_000016.9:g.89577853A>G	NM_003119.3:c.286+853A>G	NP_003110.1:p.?	16	89577853	A	G	YES	NO	NO	YES	NO	YES	NO	NO	NO	NO	NO	NO	YES	YES	NO	**PATHOGENIC**	SCV003920834
**SPG7**	IDSPG23	AR (Homozygous)	nonframeshift deletion	NC_000016.9:g.89597161_89597163del	NM_003119.3:c.932_934del	NP_003110.1:p.(Val311del)	16	89597159	CGTG	C	NO	NO	NO	NO	NO	YES	NO	YES	YES	NO	YES	NO	NO	YES	NO	**LIKELY PATHOGENIC**	SCV003920835
**SPG7**	IDSPG30	AR (Homozygous)	nonsynonymous SNV	NC_000016.9:g.89620266G>A	NM_003119.2:c.2001G>A	NP_003110.1:p.(Met667Ile)	16	89620266	G	A	NO	NO	NO	NO	YES	YES	NO	NO	NO	NO	YES	NO	YES	YES	NO	**LIKELY PATHOGENIC**	SCV003920836
**SPG7**	IDSPG33	AR (Homozygous)	frameshift deletion	NC_000016.9:g.89613070_89613078del	NM_003119.3:c.1454_1462del	NP_003110.1:p.(Arg485_Glu487del)	16	89613064	AGGAGAGGCG	A	YES	NO	NO	NO	NO	YES	NO	NO	NO	NO	YES	NO	YES	YES	YES	**PATHOGENIC**	SCV003920837
**SPTAN1**	IDSPG134	AD	nonsynonymous SNV	NC_000009.11:g.131329074C>T	NM_001130438.2:c.55C>T	NP_001123910.1:p.(Arg19Trp)	9	131329074	C	T	NO	NO	YES	NO	NO	YES	NO	NO	NO	NO	NO	YES	YES	YES	YES	**PATHOGENIC**	SCV003920838
**SPTBN2**	IDSPG125	AD	frameshift deletion	NC_000011.9:g.66457740del	NM_006946.2:c.5581del	NP_008877.1:p.(Asp1861ThrfsTer59)	11	66457738	TC	T	YES	NO	NO	NO	NO	YES	NO	NO	NO	NO	NO	NO	NO	YES	NO	**PATHOGENIC**	SCV003920839
**SVBP**	IDSPG8	AR (Homozygous)	nonsynonymous SNV	NC_000001.10:g.43273140A>G	NM_199342.3:c.146T>C	NP_955374.1:p.(Leu49Pro)	1	43273140	A	G	NO	NO	NO	YES	NO	YES	NO	NO	NO	NO	YES	NO	NO	YES	NO	**LIKELY PATHOGENIC**	SCV003920840
**SVBP**	IDSPG46	AR (Homozygous)	nonsynonymous SNV	NC_000001.10:g.43273140A>G	NM_199342.3:c.146T>C	NP_955374.1:p.(Leu49Pro)	1	43273140	A	G	NO	NO	NO	YES	NO	YES	NO	NO	NO	NO	YES	NO	NO	YES	NO	**LIKELY PATHOGENIC**	SCV003920840

Gene	ID	Inheritance	Type	Genomic GRCh37 description	Transcript description	Protein description	Chr	VCF Genomic	Reference	Alternative	PVS1	PS1	PS2	PS3	PM1	PM2	PM3	PM4	PM5	PM6	PP1	PP2	PP3	PP4	PP5	CONCLUSION	ClinVar accession numbers
**TAF1**	IDSPG71	X-Linked	nonsynonymous SNV	NC_000023.10:g.70679465G>T	NM_001286074.1:c.5194G>T	NP_001273003.1:p.(Ala1732Ser)	X	70679465	G	T	NO	NO	NO	NO	NO	**YES**	NO	NO	NO	NO	NO	**YES**	NO	**YES**	NO	**VUS**	SCV003920841
**TMEM240**	IDSPG92	AD	nonsynonymous SNV	NC_000001.10:g.1470752G>A	NM_001114748.1:c.509C>T	NP_001108220.1:p.(Pro170Leu)	1	1470752	G	A	NO	NO	**YES**	NO	NO	**YES**	NO	NO	NO	NO	NO	**YES**	NO	**YES**	**YES**	**PATHOGENIC**	SCV003920741
**TRMT5**	IDSPG120	AR (Compound Heterozygous)	frameshift deletion	NC_000014.8:g.61446302_61446305del	NM_020810.3:c.312_315del	NP_065861.3:p.(Ile105SerfsTer4)	14	61446300	CTATT	C	**YES**	NO	NO	**YES**	NO	**YES**	NO	NO	NO	NO	NO	NO	NO	**YES**	**YES**	**PATHOGENIC**	SCV003920742
**TRMT5**	IDSPG120	AR (Compound Heterozygous)	nonsynonymous SNV	NC_000014.8:g.61445951A>G	NM_020810.3:c.665T>C	NP_065861.3:p.(Ile222Thr)	14	61445951	A	G	NO	NO	NO	NO	NO	**YES**	**YES**	NO	NO	NO	NO	NO	**YES**	**YES**	**YES**	**LIKELY PATHOGENIC**	SCV003920743
**UBAP1**	IDSPG76	AD	frameshift deletion	NC_000009.11:g.34241499_34241500del	NM_016525.4:c.476_477del	NP_057609.2:p.(Phe159Ter)	9	34241497	CTT	C	**YES**	NO	NO	NO	NO	**YES**	NO	NO	NO	NO	**YES**	NO	NO	**YES**	NO	**PATHOGENIC**	SCV003920744

Table S5. ACMG criteria for identified variant classification.

Table S6

Gene	OMIM Associated Phenotype	MIM Number	Inheritance mode	Cases
POLR3A	Leukodystrophy, hypomyelinating, 7, with or without oligodontia and/or hypogonadotropic hypogonadism	607694	AR	5
RFC1	Cerebellar ataxia, neuropathy, and vestibular areflexia syndrome	614575	AR	5
SPG11	Spastic paraplegia 11, autosomal recessive,	604360	AR	5
BSCL2	Silver spastic paraplegia syndrome	260685	AD	4
SPAST	Spastic paraplegia 4, autosomal dominant	182601	AD	4
SPG7	Spastic paraplegia 7, autosomal recessive	607259	AR	4
ATL1	Spastic paraplegia 3A, autosomal dominant	182600	AD	3
KIF1A	Spastic paraplegia 30, autosomal recessive / Spastic paraplegia 30, autosomal dominant	601357	AD/AR	3
CACNA1A	Episodic ataxia, type 2	108500	AD	2
IFIH1	Aicardi-Goutieres syndrome 7	615846	AD	2
IRF2BPL	Neurodevelopmental disorder with regression, abnormal movements, loss of speech, and seizures	618088	AD	2
IRF2BPL	Neurodevelopmental disorder with ataxia, hypotonia, and microcephaly	618569	AR	2
PI4KA	Polymicrogyria, perisylvian, with cerebellar hypoplasia and arthrogryposis	616531	AR	2
REEP1	Spastic paraplegia 31, autosomal dominant	610250	AD	2
SEPSECS	Pontocerebellar hypoplasia type 2D	613811	AR	2
SVBP	Neurodevelopmental disorder with ataxia, hypotonia, and microcephaly	618569	AR	2
ACER3	Leukodystrophy, progressive, early childhood-onset	617762	AR	1
AFG3L2	Spinocerebellar ataxia 28 / Spastic ataxia 5, autosomal recessive	610246	AD	1
ALS2	Spastic paralysis, infantile onset ascending	607225	AR	1
AMPD2	Spastic paraplegia 63	515686	AR	1
AP4B1	Spastic paraplegia 47, autosomal recessive	614066	AR	1
BCKDK	Branched-chain ketoacid dehydrogenase kinase deficiency	614923	AR	1
CAPN1	Spastic paraplegia 76, autosomal recessive	616907	AR	1
CAPN10	*		AR	1
CC2D2A	Joubert syndrome 9	612285	AR	1
COL6A3	Dystonia 27	616411	AR	1
CTNNB1	Neurodevelopmental disorder with spastic diplegia and visual defects	615075	AD	1
CYP2U1	Spastic paraplegia 56, autosomal recessive	615030	AR	1
DDHD2	Spastic paraplegia 54, autosomal recessive	615033	AR	1
DLG4	Intellectual developmental disorder 62	618793	AD	1

Gene	OMIM Associated Phenotype	MIM Number	Inheritance mode	Cases
DSTYK	Spastic paraplegia 23	270750	AR	1
ERBB4	Amyotrophic lateral sclerosis 19	615515	AD	1
FA2H	Spastic paraplegia 35, autosomal recessive	612319	AR	1
FARS2	Spastic paraplegia 77, autosomal recessive	617046	AR	1
GFAP	Alexander disease	203450	AD	1
IQSEC2	Intellectual developmental disorder, X-linked 1	309530	XLD	1
KCNA1	Episodic ataxia/myokymia syndrome	160120	AD	1
KIDINS220	Spastic paraplegia, intellectual disability, nystagmus, and obesity	617296	AD	1
KIF5A	Spastic paraplegia 10, autosomal dominant	604187	AD	1
KMT2B	Dystonia 28, childhood-onset	617284	AD	1
L2HGDH	L-2-hydroxyglutaric aciduria	236792	AR	1
LAMA1	Poretti-Boltshauser syndrome	615960	AR	1
LONP1	CODAS syndrome	600373	AR	1
MMUT	Methylmalonic aciduria, mut(0) type	251000	AR	1
MORC2	Charcot-Marie-Tooth disease, axonal, type 2Z	616688	AD	1
NDUFS6	Mitochondrial complex I deficiency, nuclear type 9	618232	AR	1
PAX6	Aniridia	106210	AD	1
PCYT2	Spastic paraplegia 82, autosomal recessive	618770	AR	1
PDK3	Charcot-Marie-Tooth disease, X-linked dominant, 6	300905	XLD	1
PMM2	Congenital disorder of glycosylation, type Ia	212065	AR	1
PNKP	Ataxia-oculomotor apraxia 4	616267	AR	1
PNPLA6	Spastic paraplegia 39, autosomal recessive	612020	AR	1
POLG	Mitochondrial recessive ataxia syndrome	607459	AR	1
POLR3B	**		AD	1
RNASEH2B	Aicardi-Goutieres syndrome 2	610181	AR	1
SARS1	***		AD	1
SHMT2	Neurodevelopmental disorder with cardiomyopathy, spasticity, and brain abnormalities	619121	AR	1
SLC25A46	Neuropathy, hereditary motor and sensory, type VIB	616505	AR	1
SLC35B2	****		AR	1
SPTAN1	Developmental and epileptic encephalopathy 5	613477	AD	1
SPTBN2	Spinocerebellar ataxia 5	600224	AD	1

Gene	OMIM Associated Phenotype	MIM Number	Inheritance mode	Cases
TAF1	Mental retardation, X-linked, syndromic 33	314250	XLD	1
TMEM240	Spinocerebellar ataxia 21	607454	AD	1
TRMT5	Combined oxidative phosphorylation deficiency 26	616539	AR	1
UBAP1	Spastic paraplegia 80, autosomal dominant	618418	AD	1

AD: Autosomal Dominant
AR: Autosomal Recessive
* Loss of function mutations in CAPN10 have been linked to recessive intellectual disability [48]
** heterozygous mutations in POLR3B have been linked to ataxia, spasticity and demyelinating neuropathy [49]
*** heterozygous variants in SARS1 have been linked to spastic paraparesis with ataxia, global developmental delay, intellectual disability and febrile seizures [46]
**** biallelic variants in SLC35B2 have been linked to hypomyelinating leukodystrophy [50]

Table S6. List of identified genes, OMIM nomenclature and numbers of cases identified in our cohort.

Table S7

Gene	ID	Variant	Inheritance	Type	Functional testing	Description of functional tests performed at the laboratory
ACER3	IDSPG75	NP_060837.3:p.(Gly211Cys)	AR (Homozygous)	nonsynonymous SNV	**targeted lipidomics and qRT-PCR analysis**	To validate ACER3 variants, a targeted lipidomics analysis towards sphingolipids was performed demonstrating a similar lipid profile as the already published for Edvardson et al, Journal of Medical Genetics 2016 [30]. Moreover, qRT-PCR analysis showed decreased mRNA expression of ACER3 in patients' fibroblasts.
FA2H	IDSPG10	NP_077282.3:p.(Lys262Thr)	AR (Homozygous)	nonsynonymous SNV	**microarray and methylation assay**	Sanger analysis revealed that SPG-10 patient was homozygous for p.(Lys262Thr) variant whereas the father was heterozygous carrier and the mother did not carry FA2H mutation. A microsatellite array revealed uniparental disomy (UPD) leading to homozygous FA2H mutation. UPD was confirmed by microarray analysis and methylation profiling [51,52].
GFAP	IDSPG4	NP_001124491.1:p.(Gly18Val)	AD	nonsynonymous SNV	**transfection assay**	GFAP transfection assay showed that U251-MG cells transfected with GFAP-EGFP-expressing plasmid carrying the mutation (p.Gly18Val) resulted into an enlarged cell size compared to cells transfected with wild-type GFAP-EGFP [24].
KCNA1	IDLNF52	NP_000208.2:p.(Val368Leu)	AR (Homozygous)	nonsynonymous SNV	**cellular assay and patch clamp recording**	Patch-clamp studies of variant p.Val368Leu revealed that mutant protein alone failed to produce functional channels in homozygous state, while coexpression with wild-type produced no effects on K+ currents, similar to wild-type protein alone. These findings are concordant with the reported mode of inheritance in this family and the severity of the patient's phenotype [45].
KIDINS220	IDSPG118	NM_020738.3:c.4054-1G>C	AD	splicing	**cDNA analysis**	Sanger sequencing of PBMC cDNA revealed that c.4054-1G>C results into skipping of 337 bp, resulting into a truncated transcript (p.Ser1352Glyfs*44) not targeted by NMD. Truncated C-term of *KIDINS220* is a constant finding in KIDINS220-related patients.
LAMA1	IDSPG56	NM_005559.3:c.1423-12C>G	AR (Compound Heterozygous)	non-canonical splicing	**minigene and qRT-PCR analysis**	To confirm the association between the c.1423-12 C>G *LAMA1* variant and the partial inclusion of exon 11, we performed a minigene splicing assay. Semi-quantitative fluorescent RT-PCR revealed a normal/abnormal-splicing ratio of 0.8 for the wild-type allele and 0.2 for the mutated allele. This intronic mutation resulted into generation of various isoforms in the minigene splicing assay, showing that ~20% residual wild-type isoform is still expressed by the intronic-mutated allele alone.
PCYT2	IDSPG27	NP_001171846.1:p.(Lys319Asn)	AR (Homozygous)	nonsynonymous SNV + splicing	**targeted lipidomics and cDNA analysis**	A targeted lipidomics analysis towards phospholipids was performed demonstrating a similar lipid profile as the already published for Vaz et al 48. Moreover, cDNA analysis showed two different transcripts for Case1: the first carried the missense variant, whereas the second used an alternative donor site in intron 11, resulting in the inclusion of 102 bp into the coding sequence, leading to the insertion of 34 amino as described in Vélez-Santamaría et al [53].
PI4KA	IDSPG16	NP_477352.3:p.(Thr2053SerfsTer4)	AR (Compound Heterozygous)	frameshift deletion	**targeted lipidomics**	A targeted lipidomics analysis detecting phosphatidylinositol (PI) and its phosphorylated forms (PIP and PIP2) was performed. All of the patients showed a significantly decreased PIP/PI ratio compared to age-matched controls, indicating decreased PI4KA activity in these patients.Moreover, Western Blot with an antibody anti-PI4KA (12411-1-AP Proteintech) corroborated lower protein levels. Finally, immunofluorescence detecting decreased reaction product with an antibody anti-PI(4)P (Z-P004, Echelon Biosciences Inc.) was performed as described in Verdura et al [54].
PI4KA	IDSPG16	NP_477352.3:p.(Glu1820del)	AR (Compound Heterozygous)	frameshift deletion	**targeted lipidomics**	A targeted lipidomics analysis detecting phosphatidylinositol (PI) and its phosphorylated forms (PIP and PIP2) was performed. All of the patients showed a significantly decreased PIP/PI ratio compared to age-matched controls, indicating decreased PI4KA activity in these patients.Moreover, Western Blot with an antibody anti-PI4KA (12411-1-AP Proteintech) corroborated lower protein levels. Finally, immunofluorescence detecting decreased reaction product with an antibody anti-PI(4)P (Z-P004, Echelon Biosciences Inc.) was performed as described in Verdura et al [54].
SARS1	IDSPG64	NM_006513.4:c.969+1_969+3del	AD	non-canonical splicing	**yeast complementation studies, serylation assays, Western blot and Immunofluorescence**	The *SARS1* c.969+1_969+3del variant causes the ablation of a canonical splice site in the boundary of exon 7 and intron 7. Functional analysis showed that this change causes the inclusion of 16 intronic bp into the cDNA, which results in a loss-of-function, dominant negative effect in complementation assays in S. cerevisiae and serylation assays [46].
SEPSECS	IDLNF67, IDLNF121	NM_016955.3:c.114+3A>G	AR (Homozygous)	non-canonical splicing	**minigene and cDNA analysis**	Minigene splicing assay revealed that variant c.32+3A>G results into skipping of *SEPSECS*'s exon 1, which contains the ATG start codon, and thus results into a loss of function. This skipping was also confirmed in PBMC's cDNA from a patient carrying the same variant in homozygosis.

Gene	ID	Variant	Inheritance	Type	Functional testing	Description of functional tests performed at the laboratory
SHMT2	IDSPG26	NP_005403.2:p.(Pro499Ala)	AR (Homozygous)	nonsynonymous SNV	**targeted metabolomics, mitochondrial redox metabolism**	A targeted metabolites analysis towards key pathways catalyzed by this enzyme was performed detecting a significant decrease in glycine/serine ratio and an increase in 5-methyltetrahydrofolate/folate ratio compared to controls. Moreover, a knockdown approach for disease modeling in Drosophila melanogaster was performed as described in García-Cazorla et al [55].
SLC35B2	IDLNF68	NP_835361.1:p.Arg408SerfsTer18	AR (Homozygous)	frameshift deletion	**transfection asssay, Western blot and qRT-PCR analysis**	RT-PCR analysis performed on mRNA extracted from skin fibroblasts showed a significant decrease in the level of SLC35B2 mRNA compared to controls. The SLC35B2 p.Arg408SerfsTer18 variant affected mRNA expression and protein mislocalization in transfected HEK293F cells. It also altered the sulfation of chondroitin sulfate disaccharides in the patient's fibroblasts. This loss-of-function variant causes protein mislocalization from the Golgi apparatus, resulting in decreased sulfation of glycosaminoglycans [50].
SPG11	IDSPG20	NM_001160227.1:c.6138+4A>G	AR (Compound Heterozygous)	splicing	**cDNA analysis**	Sanger sequencing of PBMC cDNA revealed that variant c.6138+4A>G results into skipping of SPG11's exon 34 (134 pb), resulting into an out-of-frame transcript targeted by NMD.
SPG7	IDSPG21	NM_003119.3:c.286+853A>G	AR (Compound Heterozygous)	splicing	**cDNA analysis**	Sanger sequencing of fibroblast cDNA revealed that c.286+853A>G results into creation of a transcript which includes a 75 bp pseudoexon located in *SPG7* 's intron 2. This in-frame pseudoexon includes at least two codon stops. Western Blot showed reduced levels of SPG7, confirming a loss of function effect [47].
SVBP	IDSPG8, IDSPG46	NP_955374.1:p.(Leu49Pro)	AR (Homozygous)	nonsynonymous SNV	**transfection asssay, Western blot and Immunofluorescence**	Western blot and Immunofluorescence for detyrosinated tyrosin, after paclitaxel treatment to increase deTyr-tubulin pools revealed a strong decrease of deTyr-tubulin levels in patients compared to controls. We have performed cotransfection studies of VASH1 and SVBP (WT/MUT) in HeLa cells showing that p.Leu49Pro variant reduces VASH1 and SVBP soluble quantities.

Table S7. Cases with functionally validated variants

Table S8

GOMFID	Pvalue	OddsRatio	ExpCount	Count	Size	Term
GO:0016491	7.47E-15	2.41	74.08	138	430	oxidoreductase activity
GO:0004812	4.11E-13	46.25	3.62	19	21	aminoacyl-tRNA ligase activity
GO:0016875	4.11E-13	46.25	3.62	19	21	ligase activity, forming carbon-oxygen bonds
GO:0016597	1.75E-11	8.05	7.75	28	45	amino acid binding
GO:0015631	1.78E-09	2.55	36.52	72	212	tubulin binding
GO:0003954	3.37E-09	13.77	3.96	17	23	NADH dehydrogenase activity
GO:0008137	3.37E-09	13.77	3.96	17	23	NADH dehydrogenase (ubiquinone) activity
GO:0050136	3.37E-09	13.77	3.96	17	23	NADH dehydrogenase (quinone) activity
GO:0003682	9.75E-09	2.00	67.02	111	389	chromatin binding
GO:0051536	1.02E-08	6.59	6.89	23	40	iron-sulfur cluster binding
GO:0051540	1.02E-08	6.59	6.89	23	40	metal cluster binding
GO:0016874	1.37E-08	3.65	15.33	38	89	ligase activity
GO:0000049	3.70E-08	8.75	4.82	18	28	tRNA binding
GO:0051539	3.70E-08	8.75	4.82	18	28	4 iron, 4 sulfur cluster binding
GO:0031406	7.09E-08	2.76	23.95	50	139	carboxylic acid binding
GO:0030554	1.11E-07	1.55	167.28	227	971	adenyl nucleotide binding
GO:0032559	1.34E-07	1.55	165.90	225	963	adenyl ribonucleotide binding
GO:0022890	1.92E-07	1.99	54.78	91	318	inorganic cation transmembrane transporter activity
GO:0008324	2.22E-07	1.95	58.06	95	337	cation transmembrane transporter activity
GO:0022836	3.21E-07	2.34	32.04	60	186	gated channel activity
GO:0043177	3.24E-07	2.58	24.98	50	145	organic acid binding
GO:0005244	3.37E-07	2.97	18.26	40	106	voltage-gated ion channel activity
GO:0022832	3.37E-07	2.97	18.26	40	106	voltage-gated channel activity
GO:0016651	3.67E-07	4.22	9.65	26	56	oxidoreductase activity, acting on NAD(P)H
GO:0008134	5.90E-07	1.77	76.83	117	446	transcription factor binding
GO:0005524	6.99E-07	1.52	159.53	214	926	ATP binding
GO:0015075	7.13E-07	1.75	78.73	119	457	ion transmembrane transporter activity
GO:0008017	7.92E-07	2.43	27.05	52	157	microtubule binding
GO:0022824	1.75E-06	4.64	7.41	21	43	transmitter-gated ion channel activity
GO:0022835	1.75E-06	4.64	7.41	21	43	transmitter-gated channel activity
GO:0140101	1.75E-06	4.64	7.41	21	43	catalytic activity, acting on a tRNA
GO:0022843	1.94E-06	3.22	13.44	31	78	voltage-gated cation channel activity
GO:0016655	2.19E-06	5.89	5.34	17	31	oxidoreductase activity, acting on NAD(P)H, quinone or similar compound as acceptor
GO:0022857	2.42E-06	1.65	92.17	133	535	transmembrane transporter activity
GO:0015318	3.83E-06	1.71	74.60	111	433	inorganic molecular entity transmembrane transporter activity
GO:0046873	4.14E-06	2.02	39.62	67	230	metal ion transmembrane transporter activity
GO:0098960	6.81E-06	4.08	7.92	21	46	postsynaptic neurotransmitter receptor activity
GO:0019842	8.92E-06	2.91	14.30	31	83	vitamin binding
GO:0005215	9.70E-06	1.56	105.60	146	613	transporter activity
GO:0044877	9.71E-06	1.46	154.53	202	897	protein-containing complex binding
GO:0015267	1.15E-05	1.91	43.07	70	250	channel activity
GO:0022803	1.15E-05	1.91	43.07	70	250	passive transmembrane transporter activity
GO:0005216	1.35E-05	1.97	38.59	64	224	ion channel activity
GO:0015077	1.83E-05	1.96	37.38	62	217	monovalent inorganic cation transmembrane transporter activity
GO:0030594	2.13E-05	3.09	11.54	26	67	neurotransmitter receptor activity
GO:0016595	2.47E-05	19.32	1.72	8	10	glutamate binding
GO:0019904	2.97E-05	1.59	84.59	119	491	protein domain specific binding
GO:1904315	2.98E-05	4.34	6.20	17	36	transmitter-gated ion channel activity involved in regulation of postsynaptic membrane potential
GO:0001540	3.37E-05	3.15	10.51	24	61	amyloid-beta binding
GO:0008094	4.09E-05	2.86	12.58	27	73	DNA-dependent ATPase activity

Table S8. Molecular function GO terms enriched in the HSP network. Top 50 molecular function (MF) GO terms enriched in the HSP network integrating proteins by using a hypergeometric distribution function statistical analysis. GOMFID, Gene Ontology term identification number; P value, probability value for each GO term tested; OddsRatio, the strength of the association; ExpCount, the expected number of selected genes annotated at the GO term; Count, the number of genes present in the HSP network that are annotated at the GO term, Size, the number of total proteins that are annotated at the GO term; Term, the GO term name.

Table S9

GOBPID	Pvalue	OddsRatio	ExpCount	Count	Size	Term
GO:0007417	7.18E-32	2.86	122.28	245	700	central nervous system development
GO:0060322	5.91E-25	2.80	94.86	191	543	head development
GO:0030182	2.81E-24	2.29	162.29	280	929	neuron differentiation
GO:0048666	1.01E-23	2.39	137.31	246	786	neuron development
GO:0019752	2.50E-23	2.49	119.31	221	683	carboxylic acid metabolic process
GO:0007610	4.75E-23	3.01	72.85	155	417	behavior
GO:0043436	3.36E-22	2.38	128.22	230	734	oxoacid metabolic process
GO:0007420	4.79E-22	2.70	89.62	177	513	brain development
GO:0006082	6.53E-22	2.35	130.32	232	746	organic acid metabolic process
GO:0120036	8.22E-22	2.18	165.43	277	947	plasma membrane bounded cell projection organization
GO:0099536	8.30E-21	2.59	92.41	178	529	synaptic signaling
GO:0055114	1.45E-20	2.47	103.76	193	594	oxidation-reduction process
GO:0030030	1.74E-20	2.12	168.57	277	965	cell projection organization
GO:0099537	2.42E-20	2.60	89.62	173	513	trans-synaptic signaling
GO:0060284	2.42E-20	2.38	114.60	207	656	regulation of cell development
GO:0007268	4.06E-20	2.60	88.57	171	507	chemical synaptic transmission
GO:0098916	4.06E-20	2.60	88.57	171	507	anterograde trans-synaptic signaling
GO:0031175	5.46E-20	2.31	122.11	216	699	neuron projection development
GO:0051960	3.12E-19	2.35	110.05	198	630	regulation of nervous system development
GO:0050767	3.76E-19	2.44	99.05	183	567	regulation of neurogenesis
GO:0042063	1.47E-18	3.60	39.48	95	226	gliogenesis
GO:0045333	2.82E-18	5.53	20.09	61	115	cellular respiration
GO:0006520	6.11E-18	3.49	40.18	95	230	cellular amino acid metabolic process
GO:0050905	6.93E-18	8.41	12.40	45	71	neuromuscular process
GO:1901566	8.66E-18	2.02	167.35	267	958	organonitrogen compound biosynthetic process
GO:0048589	1.36E-17	2.54	79.31	152	454	developmental growth
GO:0050877	1.46E-17	2.23	115.82	201	663	nervous system process
GO:1901698	3.02E-17	2.08	141.50	233	810	response to nitrogen compound
GO:0010243	3.08E-17	2.13	132.06	221	756	response to organonitrogen compound
GO:0015980	3.60E-17	3.87	31.79	80	182	energy derivation by oxidation of organic compounds
GO:0007626	4.76E-17	4.62	23.76	66	136	locomotory behavior
GO:0030900	7.38E-17	3.12	46.64	103	267	forebrain development
GO:1901214	2.42E-16	3.22	42.10	95	241	regulation of neuron death
GO:0006811	2.99E-16	1.94	171.54	267	982	ion transport
GO:0010001	3.93E-16	3.94	28.65	73	164	glial cell differentiation
GO:0009790	5.09E-16	2.13	119.31	201	683	embryo development
GO:0070997	7.50E-16	2.99	47.34	102	271	neuron death
GO:0032990	1.07E-15	2.35	85.77	156	491	cell part morphogenesis
GO:0048812	2.16E-15	2.36	81.93	150	469	neuron projection morphogenesis
GO:0055085	2.53E-15	1.95	156.17	245	894	transmembrane transport
GO:0022904	2.65E-15	7.36	11.88	41	68	respiratory electron transport chain
GO:0120039	2.95E-15	2.34	83.68	152	479	plasma membrane bounded cell projection morphogenesis
GO:0032989	3.56E-15	2.22	96.60	169	553	cellular component morphogenesis
GO:0048667	4.04E-15	2.42	75.12	140	430	cell morphogenesis involved in neuron differentiation
GO:0048858	5.38E-15	2.32	84.20	152	482	cell projection morphogenesis
GO:0034762	1.06E-14	2.58	61.49	120	352	regulation of transmembrane transport
GO:0009628	1.11E-14	1.93	151.45	237	867	response to abiotic stimulus
GO:0061564	1.17E-14	2.50	66.56	127	381	axon development
GO:0006812	2.08E-14	2.04	120.88	198	692	cation transport
GO:0000904	2.61E-14	2.20	92.41	161	529	cell morphogenesis involved in differentiation

Table S9. Biological process GO terms enriched in the HSP network. Top 50 biological process (BP) GO terms enriched in the HSP network integrating proteins by using a hypergeometric distribution function statistical analysis. GOBPID, Gene Ontology term identification number; P value, probability value for each GO term tested; OddsRatio, the strength of the association; ExpCount, the expected number of selected genes annotated at the GO term; Count, the number of genes present in the HSP network that are annotated at the GO term, Size, the number of total proteins that are annotated at the GO term; Term, the GO term name.

Table S10

GOCCID	Pvalue	OddsRatio	ExpCount	Count	Size	Term
GO:0005739	9.45E-29	2.56	143.36	267	831	mitochondrion
GO:0043005	9.09E-23	2.25	156.82	269	909	neuron projection
GO:0005759	2.30E-22	3.97	40.89	104	237	mitochondrial matrix
GO:0030424	3.87E-22	2.86	77.46	160	449	axon
GO:0045202	6.47E-20	2.12	161.82	267	938	synapse
GO:0036477	3.52E-16	2.22	104.20	182	604	somatodendritic compartment
GO:0098796	1.40E-14	2.02	125.08	204	725	membrane protein complex
GO:0098803	3.53E-14	11.28	7.42	30	43	respiratory chain complex
GO:0098794	9.32E-14	2.27	79.19	142	459	postsynapse
GO:0044297	5.86E-13	2.26	74.87	134	434	cell body
GO:0097060	8.93E-13	2.72	45.03	92	261	synaptic membrane
GO:0070469	3.62E-12	7.58	8.80	31	51	respirasome
GO:0005746	1.06E-11	7.87	8.11	29	47	mitochondrial respirasome
GO:0005743	1.57E-11	2.86	35.37	75	205	mitochondrial inner membrane
GO:1990204	1.91E-11	5.71	11.21	35	65	oxidoreductase complex
GO:0043025	4.15E-11	2.21	66.07	117	383	neuronal cell body
GO:0005740	4.39E-11	2.24	63.14	113	366	mitochondrial envelope
GO:0045211	6.08E-11	2.96	30.88	67	179	postsynaptic membrane
GO:0019866	6.08E-11	2.60	41.75	83	242	organelle inner membrane
GO:0031966	8.59E-11	2.25	60.04	108	348	mitochondrial membrane
GO:0032838	6.99E-10	3.72	17.60	44	102	plasma membrane bounded cell projection cytoplasm
GO:0005747	7.84E-10	14.56	4.14	18	24	mitochondrial respiratory chain complex I
GO:0030964	7.84E-10	14.56	4.14	18	24	NADH dehydrogenase complex
GO:0045271	7.84E-10	14.56	4.14	18	24	respiratory chain complex I
GO:1990351	9.21E-10	2.71	32.61	67	189	transporter complex
GO:0030425	1.74E-09	2.00	73.66	122	427	dendrite
GO:0120111	1.82E-09	5.04	10.52	31	61	neuron projection cytoplasm
GO:0097447	2.38E-09	1.99	74.01	122	429	dendritic tree
GO:0031967	6.10E-09	1.77	109.72	165	636	organelle envelope
GO:0031975	6.10E-09	1.77	109.72	165	636	envelope
GO:0044304	6.41E-09	5.48	8.80	27	51	main axon
GO:1902495	8.55E-09	2.59	32.09	64	186	transmembrane transporter complex
GO:0098590	1.36E-08	1.66	139.39	199	808	plasma membrane region
GO:0099568	1.41E-08	2.96	22.94	50	133	cytoplasmic region
GO:0005773	3.60E-08	1.82	85.22	132	494	vacuole
GO:0098800	4.90E-08	4.09	11.73	31	68	inner mitochondrial membrane protein complex
GO:0034702	6.04E-08	2.55	29.33	58	170	ion channel complex
GO:1904115	6.31E-08	6.37	6.38	21	37	axon cytoplasm
GO:0098798	7.68E-08	2.99	20.01	44	116	mitochondrial protein complex
GO:0098793	8.47E-08	1.92	65.56	106	380	presynapse
GO:0000323	1.04E-07	1.84	76.94	120	446	lytic vacuole
GO:0005764	1.04E-07	1.84	76.94	120	446	lysosome
GO:0005777	1.36E-07	3.53	13.97	34	81	peroxisome
GO:0042579	1.36E-07	3.53	13.97	34	81	microbody
GO:1902494	1.51E-07	1.60	139.22	194	807	catalytic complex
GO:0005778	2.11E-07	6.07	6.21	20	36	peroxisomal membrane
GO:0031903	2.11E-07	6.07	6.21	20	36	microbody membrane
GO:0042175	2.47E-07	1.69	101.10	148	586	nuclear outer membrane-endoplasmic reticulum membrane network
GO:0043202	2.92E-07	3.55	13.11	32	76	lysosomal lumen
GO:1990391	6.21E-07	6.86	5.00	17	29	DNA repair complex

Table S10. Cellular compartment GO terms enriched in the HSP network. Top 50 cellular compartment (CC) GO terms enriched in the HSP network integrating proteins by using a hypergeometric distribution function statistical analysis. GOCCID, Gene Ontology term identification number; P value, probability value for each GO term tested; OddsRatio, the strength of the association; ExpCount, the expected number of selected genes annotated at the GO term; Count, the number of genes present in the HSP network that are annotated at the GO term, Size, the number of total proteins that are annotated at the GO term; Term, the GO term name.

Table S11

Symbol	Full name	geneID	Classif.	lof_z	mis_z	pLI	pRec	pNull
ACHE	acetylcholinesterase (Cartwright blood group)	43	candidate	4.25	2.75	9.98E-01	1.73E-03	7.25E-09
ADM	adrenomedullin	133	candidate	1.31	-0.107	4.08E-02	8.53E-01	1.06E-01
AGER	advanced glycosylation end-product specific receptor	177	candidate	0.198	8.83E-02	6.35E-16	1.48E-02	9.85E-01
ARC	activity regulated cytoskeleton associated protein	23237	candidate	2.98	2.6	5.94E-01	4.05E-01	3.70E-04
BECN1	beclin 1	8678	candidate	4.04	1.88	9.37E-01	6.28E-02	4.15E-07
CASP3	caspase 3	836	candidate	2.37	1.66	1.00E-01	8.93E-01	6.61E-03
CCR2	C-C motif chemokine receptor 2	729230	candidate	1.63	3.39E-02	2.34E-02	9.19E-01	5.72E-02
CNR1	cannabinoid receptor 1	1268	candidate	2.42	2.62	5.07E-01	4.90E-01	3.65E-03
CRK	CRK proto-oncogene, adaptor protein	1398	candidate	3.27	2.37	9.59E-01	4.06E-02	1.67E-05
CSF2	colony stimulating factor 2	1437	candidate	2.26	0.377	8.35E-01	1.62E-01	2.95E-03
CXCL10	C-X-C motif chemokine ligand 10	3627	candidate	1.59	0.335	3.69E-01	5.87E-01	4.42E-02
CYP2B6	cytochrome P450 family 2 subfamily B member 6	1555	candidate	0.737	-0.976	1.93E-10	2.32E-01	7.68E-01
DECR1	2,4-dienoyl-CoA reductase 1	1666	candidate	-0.582	3.16E-02	4.47E-14	6.88E-03	9.93E-01
DLG4	discs large MAGUK scaffold protein 4	1742	candidate	5.46	4.93	1.00E+00	4.60E-04	4.81E-13
DNAH7	dynein axonemal heavy chain 7	56171	candidate	3.5	-1.09	1.35E-71	2.50E-02	9.75E-01
EIF2S1	eukaryotic translation initiation factor 2 subunit alpha	1965	candidate	3.39	2.86	9.70E-01	2.97E-02	7.67E-06
EIF3K	eukaryotic translation initiation factor 3 subunit K	27335	candidate	2.08	0.761	2.10E-03	9.80E-01	1.76E-02
ENO2	enolase 2	2026	candidate	3.4	1.81	3.84E-01	6.16E-01	7.86E-05
FOSB	FosB proto-oncogene, AP-1 transcription factor subunit	2354	candidate	3.45	1.55	9.76E-01	2.45E-02	4.75E-06
GABPA	GA binding protein transcription factor subunit alpha	2551	candidate	4.47	3.12	9.98E-01	1.88E-03	1.81E-09
GAP43	growth associated protein 43	2596	candidate	1.58	-6.79E-02	2.15E-02	9.15E-01	6.40E-02
GRAP2	GRB2 related adaptor protein 2	9402	candidate	2.39	1.12	1.53E-04	9.93E-01	6.52E-03
GRM5	glutamate metabotropic receptor 5	2915	candidate	5.24	3.21	9.99E-01	1.28E-03	5.85E-12
GTF2H1	general transcription factor IIH subunit 1	2965	candidate	4.53	1.8	9.90E-01	1.02E-02	4.56E-09
HDAC3	histone deacetylase 3	8841	candidate	3.93	3.72	5.67E-01	4.33E-01	3.84E-06
HDAC9	histone deacetylase 9	9734	candidate	6.32	2.06	1.00E+00	2.68E-04	2.16E-16
HMGB1	high mobility group box 1	3146	candidate	2.64	2.69	8.20E-01	1.79E-01	7.76E-04
HSD17B6	hydroxysteroid 17-beta dehydrogenase 6	8630	candidate	2.59	0.492	3.48E-01	6.49E-01	2.51E-03
HSPA4	heat shock protein family A (Hsp70) member 4	3308	candidate	5.66	2.06	1.00E+00	4.63E-04	9.89E-14
HSPA5	heat shock protein family A (Hsp70) member 5	3309	candidate	3.61	4.03	7.73E-01	2.27E-01	1.24E-05
ICAM1	intercellular adhesion molecule 1	3383	candidate	2.79	1.90E-02	3.53E-02	9.63E-01	1.46E-03
IFNB1	interferon beta 1	3456	candidate	NA	-0.759	NA	NA	NA
IL18	interleukin 18	3606	candidate	1.13	1.52	3.07E-02	8.23E-01	1.46E-01
IL1A	interleukin 1 alpha	3552	candidate	0.886	0.541	1.60E-04	6.94E-01	3.06E-01
IL2	interleukin 2	3558	candidate	1.86	1.43	4.80E-01	5.00E-01	2.05E-02
IMMT	inner membrane mitochondrial protein	10989	candidate	4.11	0.763	1.33E-02	9.87E-01	2.13E-06
ISYNA1	inositol-3-phosphate synthase 1	51477	candidate	2.14	1.27	2.49E-05	9.83E-01	1.73E-02
KCNA3	potassium voltage-gated channel subfamily A member 3	3738	candidate	3.25	3.02	8.94E-01	1.06E-01	3.88E-05
KHDRBS1	KH RNA binding domain containing, signal transduction associated 1	10657	candidate	3.91	2.42	9.94E-01	5.77E-03	1.37E-07
LTA	lymphotoxin alpha	4049	candidate	1.69	1.81	1.97E-01	7.62E-01	4.15E-02
LY6E	lymphocyte antigen 6 family member E	4061	candidate	1.45	0.574	3.21E-01	6.18E-01	6.11E-02
MAPK14	mitogen-activated protein kinase 14	1432	candidate	3.39	3.31	3.75E-01	6.25E-01	8.44E-05
MAPK3	mitogen-activated protein kinase 3	5595	candidate	2.8	1.74	3.69E-02	9.62E-01	1.36E-03
MIR146A	microRNA 146a	406938	candidate	NA	NA	NA	NA	NA
MTG1	mitochondrial ribosome associated GTPase 1	92170	candidate	2.4	-0.524	1.33E-02	9.81E-01	6.17E-03
NDUFA5	NADH:ubiquinone oxidoreductase subunit A5	4698	candidate	0.876	0.2	1.87E-03	7.41E-01	2.57E-01
NDUFA7	NADH:ubiquinone oxidoreductase subunit A7	4701	candidate	-0.201	-0.241	6.60E-05	3.00E-01	7.00E-01
NDUFB1	NADH:ubiquinone oxidoreductase subunit B1	4707	candidate	-0.554	-0.207	3.59E-05	2.19E-01	7.81E-01
NDUFV3	NADH:ubiquinone oxidoreductase subunit V3	4731	candidate	-1.5	-0.31	2.80E-18	3.17E-04	1.00E+00
NPY	neuropeptide Y	4852	candidate	1.47	0.643	1.43E-01	7.86E-01	7.10E-02
NRG1	neuregulin 1	3084	candidate	4.57	0.637	9.97E-01	3.35E-03	1.49E-09
OPRM1	opioid receptor mu 1	4988	candidate	0.526	-0.619	7.89E-11	1.43E-01	8.57E-01
PARP1	poly(ADP-ribose) polymerase 1	142	candidate	4.52	0.818	3.34E-04	1.00E+00	1.48E-07
PCYT2	phosphate cytidylyltransferase 2, ethanolamine	5833	candidate	2.62	1.73	8.64E-04	9.96E-01	2.69E-03
PDC	phosducin	5132	candidate	0.676	0.603	1.01E-04	5.94E-01	4.06E-01
PI4KA	phosphatidylinositol 4-kinase alpha	5297	candidate	6.53	3.53	3.12E-12	1.00E+00	9.17E-15
PLA2G1B	phospholipase A2 group IB	5319	candidate	-2.08	-0.709	1.01E-10	6.60E-03	9.93E-01
PLB1	phospholipase B1	151056	candidate	-0.413	-0.588	1.73E-72	2.99E-12	1.00E+00
POLDIP2	DNA polymerase delta interacting protein 2	26073	candidate	3.44	1.94	4.14E-01	5.86E-01	6.24E-05
POTEF	POTE ankyrin domain family member F	728378	candidate	1.53	-2.74	5.45E-06	8.82E-01	1.18E-01
PPARA	peroxisome proliferator activated receptor alpha	5465	candidate	2.73	1.81	3.02E-02	9.68E-01	1.84E-03
PPIG	peptidylprolyl isomerase G	9360	candidate	4.93	1.72	9.63E-01	3.71E-02	9.15E-10
RAP1B	RAP1B, member of RAS oncogene family	5908	candidate	2.96	2.6	9.10E-01	8.99E-02	1.25E-04
RNF19A	ring finger protein 19A, RBR E3 ubiquitin protein ligase	25897	candidate	4	2.38	2.53E-02	9.75E-01	4.05E-06
RNR1	RNA, ribosomal 45S cluster 1	6052	candidate	NA	NA	NA	NA	NA
ROS1	ROS proto-oncogene 1, receptor tyrosine kinase	6098	candidate	0.455	-0.501	1.62E-72	1.47E-10	1.00E+00
SELP	selectin P	6403	candidate	-0.215	-0.256	5.04E-32	1.72E-05	1.00E+00
SHMT2	serine hydroxymethyltransferase 2	6472	candidate	2.16	1.52	7.82E-08	9.77E-01	2.28E-02
SOD2	superoxide dismutase 2	6648	candidate	2.11	0.887	1.55E-01	8.30E-01	1.46E-02
SVBP	small vasohibin binding protein	374969	candidate	-0.258	0.374	1.13E-03	4.04E-01	5.95E-01
TGM2	transglutaminase 2	7052	candidate	0.703	0.536	6.81E-18	2.71E-02	9.73E-01
TXNRD1	thioredoxin reductase 1	7296	candidate	2.79	1.32	1.61E-06	9.98E-01	1.55E-03
UBAP1	ubiquitin associated protein 1	51271	candidate	3.32	0.869	9.12E-01	8.83E-02	2.42E-05
UTRN	utrophin	7402	candidate	8.61	0.371	1.00E-19	1.00E+00	8.03E-25
YWHAZ	tyrosine 3-monooxygenase/tryptophan 5-monooxygenase activation protein zeta	7534	candidate	3.13	3.1	9.40E-01	6.03E-02	4.52E-05

Table S11. Candidate genes in the HSP expanded network. Seventy-five candidate genes predicted by the prioritization tool to be associated with HSP disease. Gene constraints: lof_z, the Z-score value of being LoF intolerant; mis_z, the Z-score value of being intolerant to missense variation; pLI, the probability of being LoF intolerant; pRec, the probability of being intolerant to homozygous variation.
